# Evaluating the informativeness of deep learning annotations for human complex diseases

**DOI:** 10.1038/s41467-020-18515-4

**Published:** 2020-09-17

**Authors:** Kushal K. Dey, Bryce van de Geijn, Samuel Sungil Kim, Farhad Hormozdiari, David R. Kelley, Alkes L. Price

**Affiliations:** 1 Department of Epidemiology, Harvard T. H. Chan School of Public Health, Boston, MA USA; 2 Department of Electrical Engineering and Computer Science, Massachusetts Institute of Technology, Cambridge, MA USA; 3 Calico Labs, South San Francisco, CA USA; 4 Department of Biostatistics, Harvard T.H. Chan School of Public Health, Boston, MA USA

**Keywords:** Machine learning, Mutagenesis, Autoimmune diseases

## Abstract

Deep learning models have shown great promise in predicting regulatory effects from DNA sequence, but their informativeness for human complex diseases is not fully understood. Here, we evaluate genome-wide SNP annotations from two previous deep learning models, DeepSEA and Basenji, by applying stratified LD score regression to 41 diseases and traits (average *N* = 320K), conditioning on a broad set of coding, conserved and regulatory annotations. We aggregated annotations across all (respectively blood or brain) tissues/cell-types in meta-analyses across all (respectively 11 blood or 8 brain) traits. The annotations were highly enriched for disease heritability, but produced only limited conditionally significant results: non-tissue-specific and brain-specific Basenji-H3K4me3 for all traits and brain traits respectively. We conclude that deep learning models have yet to achieve their full potential to provide considerable unique information for complex disease, and that their conditional informativeness for disease cannot be inferred from their accuracy in predicting regulatory annotations.

## Abstract (web summary)

Deep learning models have shown great promise in predicting regulatory effects from DNA sequence. Here the authors evaluate sequence-based epigenomic deep learning models and conclude that these models are not yet ready to inform our knowledge of human disease.

## Introduction

Disease risk variants identified by genome-wide association studies (GWAS) lie predominantly in non-coding regions of the genome^1–7^, motivating broad efforts to generate genome-wide maps of regulatory marks across tissues and cell types^8–11^. Recently, deep learning models trained using these genome-wide maps have shown considerable promise in predicting regulatory marks directly from DNA sequence^12–18^. In particular, these studies showed that variant-level deep learning annotations (predictive annotations based on the reference allele) attained high accuracy in predicting the underlying chromatin marks^13–16^, and that models incorporating allelic-effect deep learning annotations (absolute value of the predicted difference between reference and variant alleles) attained high accuracy in predicting disease-associated SNPs^13–16^. Additional applications of deep learning models, including analyses of signed allelic-effect annotations, are discussed in the Discussion section. However, it is unclear whether deep learning annotations at commonly varying SNPs contain unique information for complex disease that is not present in other annotations.

Here, we evaluate the informativeness for complex disease of allelic-effect annotations at commonly varying SNPs constructed using two deep learning models previously trained on tissue-specific regulatory features (DeepSEA^13,15^ and Basenji^16^). We apply stratified LD score regression^5,19^ (S-LDSC) to 41 independent diseases and complex traits (average *N* = 320K) to evaluate each annotation’s informativeness for disease heritability conditional on the underlying variant-level annotations as well as a broad set of coding, conserved, regulatory and LD-related annotations from the baseline-LD model^19^ and other sources (imputed Roadmap and ChromHMM annotations^11,20–22^). As a secondary metric, we also evaluate the accuracy of models that incorporate deep learning annotations in predicting disease-associated or fine-mapped SNPs^23,24^. We aggregate DeepSEA and Basenji annotations across all tissues in meta-analyses across all 41 traits, across blood cell types in meta-analyses across 11 blood-related traits, and across brain tissues in meta-analyses across 8 brain-related traits.

## Results

### Overview of methods

We define a genomic annotation as an assignment of a numeric value (either binary or continuous-valued) to each SNP (Methods). Our focus is on continuous-valued annotations (with values between 0 and 1) trained by deep learning models to predict biological function from DNA sequence. Annotation values are defined for each SNP with minor allele count ≥5 in a 1000 Genomes Project European reference panel^25^, as in our previous work^5^. We have publicly released all annotations analyzed in this study (see Data availability).

In our analysis of allelic-effect (Δ) deep learning annotations across 41 traits, we analyzed 16 non-tissue-specific deep learning annotations: 8 DeepSEA annotations^13,15^ previously trained to predict 4 tissue-specific chromatin marks (DNase, H3K27ac, H3K4me1, H3K4me3) known to be associated with active promoter and enhancer regions across 127 Roadmap tissues^11,26^, aggregated using the average (Avg) or maximum (Max) across tissues, and 8 analogous Basenji annotations^16^, quantile-matched with DeepSEA annotations to lie between 0 and 1 (Table 1 and Methods). To assess whether the allelic-effect annotations provided unique information for disease, we conservatively included the underlying variant-level (V) annotations (Supplementary Table 1) as well as a broad set of coding, conserved, regulatory and LD-related annotations in our analyses: 86 annotations from the baseline-LD (v2.1) model^19^, which has been shown to effectively model LD-dependent architectures^27^; 8 Roadmap annotations^11^ (for same chromatin marks as DeepSEA and Basenji annotations), imputed using ChromImpute^20^; and 40 ChromHMM annotations^21,22^ based on 20 ChromHMM states across 127 Roadmap tissues^11^ (Supplementary Table 2). When comparing pairs of annotations that differed only in their aggregation strategy (Avg/Max), chromatin mark (DNase/H3K27ac/H3K4me1/H3K4me3), model (DeepSEA/Basenji) or type (variant-level/allelic-effect), respectively, we observed large correlations across aggregation strategies (average *r* = 0.71), chromatin marks (average *r* = 0.58), models (average *r* = 0.54) and types (average *r* = 0.48) (Supplementary Fig. 1).

**Table 1:** List of non-tissue-specific allelic-effect analyzed.

Allelic-effect annotations	Size (%)
DeepSEAΔ-DNase-Avg	0.3
DeepSEAΔ-DNase-Max	2.0
DeepSEAΔ-H3K27ac-Avg	0.2
DeepSEAΔ-H3K27ac-Max	0.9
DeepSEAΔ-H3K4me1-Avg	0.3
DeepSEAΔ-H3K4me1-Max	1.7
DeepSEAΔ-H3K4me3-Avg	0.1
DeepSEAΔ-H3K4me3-Max	0.7
BasenjiΔ-DNase-Avg	0.3
BasenjiΔ-DNase-Max	2.1
BasenjiΔ-H3K27ac-Avg	0.2
BasenjiΔ-H3K27ac-Max	0.9
BasenjiΔ-H3K4me1-Avg	0.3
BasenjiΔ-H3K4me1-Max	1.7
BasenjiΔ-H3K4me3-Avg	0.1
BasenjiΔ-H3K4me3-Max	0.7

We list the 16 allelic-effect deep learning annotations (8 DeepSEAΔ, 8 BasenjiΔ) and their annotation sizes (average annotation value across SNPs). A list of non-tissue-specific variant-level annotations is provided in Supplementary Table 1.

In our analysis of 11 blood-related traits (respectively 8 brain-related traits), we analyzed 8 DeepSEA annotations and 8 Basenji annotations that were aggregated across 27 blood cell types (respectively 13 brain tissues), instead of all 127 tissues. Details of other annotations included in these analyses are provided below.

We assessed the informativeness of these annotations for disease heritability using stratified LD score regression (S-LDSC) with the baseline-LD model^5,19^. We considered two metrics, enrichment and standardized effect size (*τ*^⋆^). Enrichment is defined as the proportion of heritability explained by SNPs in an annotation divided by the proportion of SNPs in the annotation^5^, and generalizes to continuous-valued annotations with values between 0 and 1^28^. Standardized effect size (*τ*^⋆^) is defined as the proportionate change in per-SNP heritability associated with a 1 standard deviation increase in the value of the annotation, conditional on other annotations included in the model^19^; unlike enrichment, *τ*^⋆^ quantifies effects that are unique to the focal annotation. In our “marginal” analyses, we estimated *τ*^⋆^ for each focal annotation conditional on annotations from the baseline-LD model. In our “joint” analyses, we merged baseline-LD model annotations with focal annotations that were marginally significant after Bonferroni correction and performed forward stepwise elimination to iteratively remove focal annotations that had conditionally non-significant *τ*^⋆^ values after Bonferroni correction, as in ref. ^19^. All analyses of allelic-effect annotations were further conditioned on jointly significant annotations from a variant-level analysis, if any. Distinct from evaluating deep learning annotations using S-LDSC, we also evaluated the accuracy of models that incorporate deep learning annotations in predicting disease-associated or fine-mapped SNPs^23,24^ (Methods).

### Basenji all-tissues H3K4me3 is informative for disease

We evaluated the informativeness of allelic-effect deep learning annotations for disease heritability by applying S-LDSC with the baseline-LD model^5,19^ to summary association statistics for 41 independent diseases and complex traits (average *N* = 320K); for 6 traits we analyzed two different data sets, leading to a total of 47 data sets analyzed (Supplementary Table 3). We meta-analyzed results across these 47 data sets, which were chosen to be independent^28^. The 41 traits include 27 UK Biobank traits^29^ for which summary association statistics are publicly available (see Data Availability).

Although our main focus is on allelic-effect deep learning annotations, analysis of variant-level deep learning annotations was a necessary prerequisite step, for two reasons: (i) allelic-effect annotations are computed as differences between variant-level annotations for each allele, and (ii) we wished to condition analyses of allelic-effect annotations on jointly significant variant-level annotations, if any. We thus constructed 8 variant-level DeepSEAV annotations by applying previously trained DeepSEA models^15^ (see Code availability) for each of 4 tissue-specific chromatin marks (DNase, H3K27ac, H3K4me1, H3K4me3) across 127 Roadmap tissues^11^ to 1 kb of human reference sequence around each SNP; for each chromatin mark, we aggregated variant-level DeepSEAV annotations across the 127 tissues using either the average (Avg) or maximum (Max) across tissues (Table 1 and Methods). The DeepSEA model was highly predictive of the corresponding tissue-specific chromatin marks, with AUROC values reported by ref. ^15^ ranging from 0.77−0.97 (Supplementary Table 4). We also constructed 8 variant-level BasenjiV annotations by applying previously trained Basenji models^16^ (see Code availability) and aggregating across tissues in analogous fashion (Table 1 and Methods); Basenji uses a Poisson likelihood model, unlike the binary classification approach of DeepSEA, and analyzes 130 kb of human reference sequence around each SNP using dilated convolutional layers. The constituent tissue-specific BasenjiV annotations do not lie between 0 and 1; so we transformed these annotations to lie between 0 and 1 via quantile matching with corresponding DeepSEAV annotations, to ensure a fair comparison of the two approaches (Methods). Although the variant-level DeepSEAV and BasenjiV annotations were highly enriched for heritability, we determined that none of them were conditionally informative across the 41 traits (Supplementary Figs. 2–6 and Supplementary Note). This is an expected result, because the variant-level deep learning annotations simply predict measured variant-level annotations from Roadmap that are also included in the model.

Our main focus is on allelic-effect annotations (absolute value of the predicted difference between reference and variant alleles), which have been the focus of recent work^13–16^. We evaluated the informativeness of 8 non-tissue-specific DeepSEAΔ and 8 non-tissue-specific BasenjiΔ allelic-effect annotations (Table 1) for disease heritability by applying S-LDSC to the 41 traits. Analyses of allelic-effect annotations were conditioned on the baseline-LD model plus 7 annotations from Supplementary Fig. 6. For ease of comparison, allelic-effect Basenji annotations were quantile-matched with corresponding allelic-effect DeepSEA annotations, analogous to analyses of variant-level annotations.

**Fig. 1:**
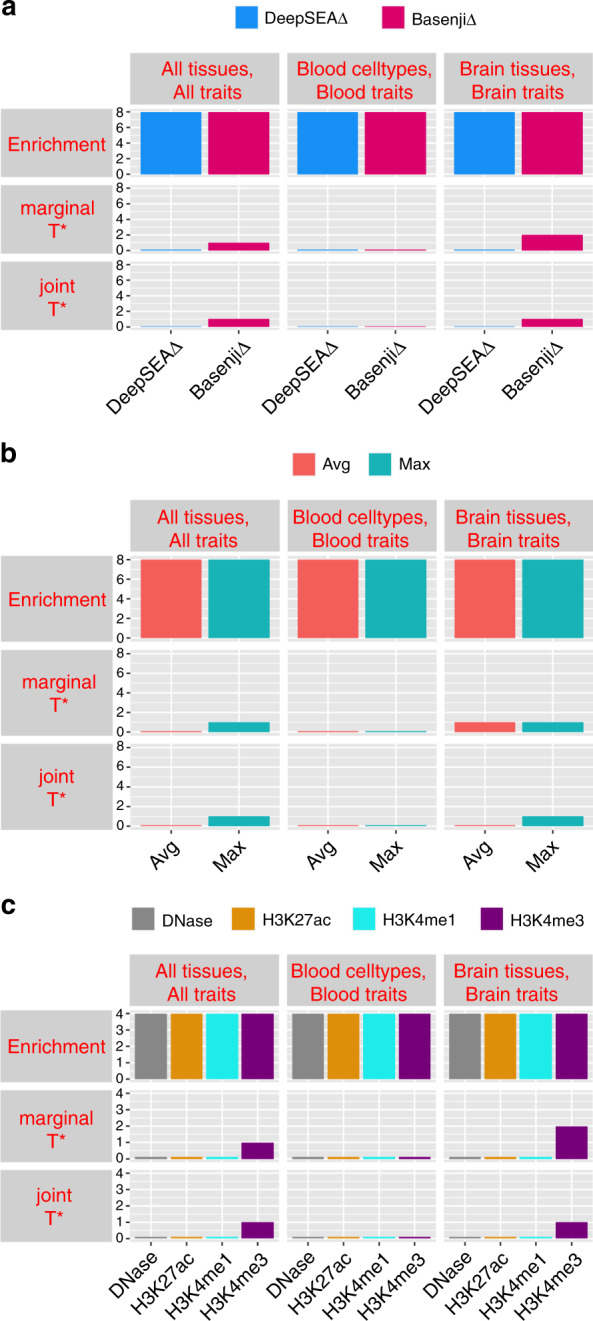
Summary of disease informativeness of allelic-effect deep learning annotations. We report the number of allelic-effect annotations with significant heritability enrichment, marginal conditional *τ*^⋆^, and joint conditional *τ*^⋆^, across **a** different deep learning models (DeepSEA/Basenji), **b** different aggregation strategies (Avg/Max) and **c** different chromatin marks (DNase/H3K27ac/H3K4me1/H3K4me3). Numerical results are reported in Supplementary Table 5 (numerical summary of results), Supplementary Table 6 (enrichment and marginal *τ*^⋆^ for all tissues, all traits analysis), Supplementary Table 15 (enrichment and marginal *τ*^⋆^ of blood cell types, blood traits analysis), Supplementary Table 21 (enrichment and marginal *τ*^⋆^ of brain tissues, brain traits analysis) and Supplementary Table 27 (joint *τ*^⋆^ of brain tissues, brain traits analysis). No Supplementary Table is needed for joint *τ*^⋆^ of all tissues, all traits (1 marginally significant annotation) or blood cell types, blood traits (0 marginally significant annotations).

**Fig. 2:**
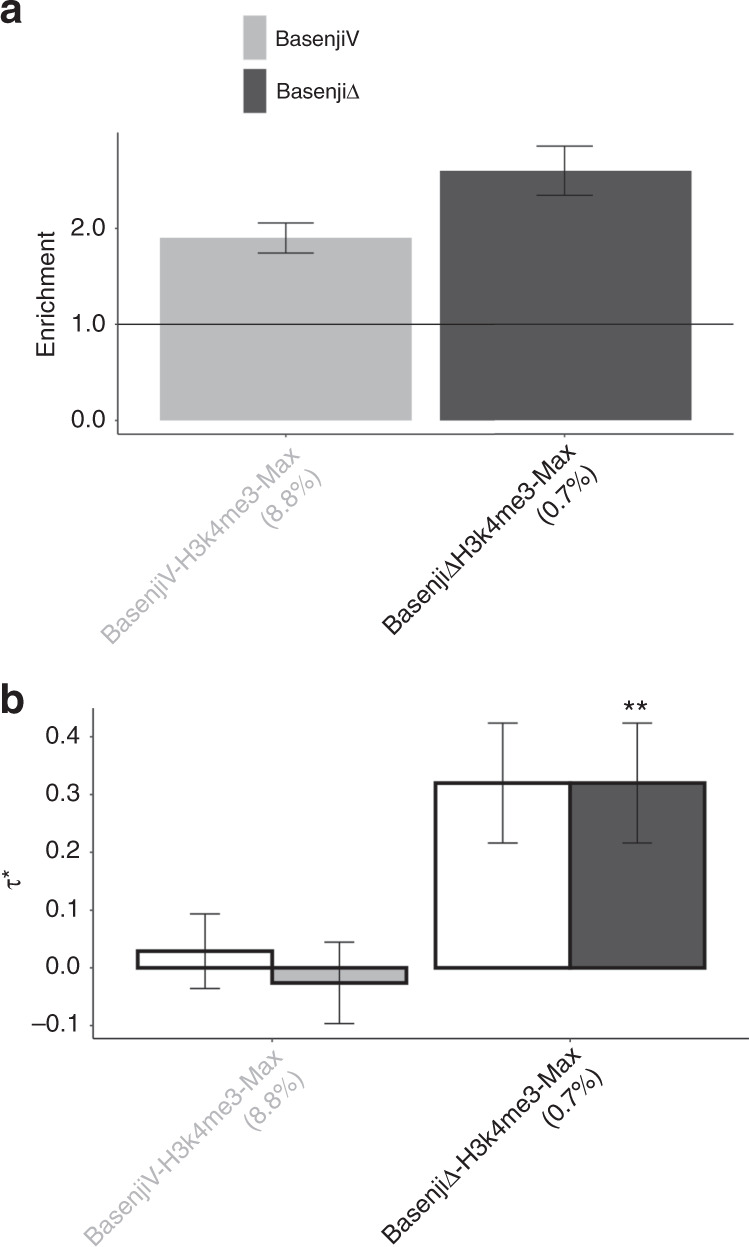
Disease informativeness of non-tissue-specific allelic-effect deep learning annotations. **a** Heritability enrichment, conditioned on the non-tissue-specific variant-level joint model. Horizontal line denotes no enrichment. **b** Standardized effect size *τ*^⋆^ conditioned on either the non-tissue-specific variant-level joint model (marginal analysis: left column, white) or the variant-level joint model plus 1 non-tissue-specific allelic-effect Basenji annotation (BasenjiΔ-H3K4me3-Max) (non-tissue-specific final joint model: right column, dark shading. Results are meta-analyzed across 41 traits. Results are displayed only for the allelic-effect annotation (BasenjiΔ-H3K4me3-Max) with significant *τ*^⋆^ in marginal analyses after correcting for 106 (variant-level + allelic-effect) non-tissue-specific annotations tested (*P* < 0.05/106), along with the corresponding variant-level annotation; the correlation between the two annotations is 0.43. For non-tissue-specific final joint model (right column), **P < 0.05/106. Error bars denote 95% confidence intervals. Numerical results are reported in Supplementary Table 6 and Supplementary Table 8.

A summary of the results is provided in Fig. 1 (All tissues, All traits column; numerical results in Supplementary Table 5), which reports the number of allelic-effect annotations of various types with significant heritability enrichment, marginal conditional signal, and joint conditional signal, respectively. In our marginal analysis of disease heritability, all allelic-effect annotations from DeepSEA and Basenji models were significantly enriched for heritability across 41 traits; the allelic-effect BasenjiΔ annotations were more enriched for disease heritability (2.40x) than allelic-effect DeepSEAΔ annotations (1.91x) (Supplementary Table 6). However, only 0 DeepSEAΔ annotations and 1 BasenjiΔ annotation, BasenjiΔ-H3K4me3-Max, attained a Bonferroni-significant standardized effect size (*τ*^⋆^) (Fig. 2 and Supplementary Table 6); results were similar when conditioned on just the baseline-LD model (Supplementary Table 7). Despite the high correlation between variant-level and allelic-effect annotations (*r* = 0.48; Supplementary Fig. 1), the corresponding variant-level annotation (BasenjiV-H3K4me3-Max) did not produce significant conditional signal (Fig. 2 and Supplementary Table 8), consistent with Supplementary Fig. 2). We note that since BasenjiΔ-H3K4me3-Max was the only marginally significant annotation in the non-tissue-specific allelic-effect analysis, it is automatically jointly significant.

To assess the impact of conditioning on conservation-related annotations, we performed a marginal analysis in which we no longer conditioned on the 11 conservation-related annotations of the baseline-LD model (e.g. GERP++^19,30^, PhastCons^31^, conservation across 29 mammals^32^, Background selection statistic^33^; Supplementary Table 9). In this analysis, 6 DeepSEAΔ and 4 BasenjiΔ produced Bonferroni-significant conditional signals (Supplementary Table 10). This implies that conditioning on conservation-related annotations had a major impact on our primary analysis. Consistent with this finding, we observed substantial correlations (up to *r* = 0.24) between allelic-effect annotations and conservation-related annotations (Supplementary Fig. 7). These results can be viewed as a proof-of-concept that allelic-effect annotations can uncover biological signals.

We investigated the *k*-mer composition of regions proximal to the BasenjiΔ-H3K4me3-Max annotation. For each of all 682 possible *k*-mers with 1 ≤ *k* ≤ 5 (merged with their reverse complements), we assessed the weighted *k*-mer enrichment in 1kb regions around each SNP in the annotation (Methods). Many CpG-related *k*-mers (*k* ≥ 3) attained Bonferroni-significant enrichments, with the largest and most significant enrichments attained by CGCGC (4.1x and *P* = 3.5−e10) and CGGCG (4.1x and *P* = 3.6e–10) (Supplementary Table 11); these were far larger and more statistically significant than enrichments for simple GC-rich motifs such as the 2-mer CpG (1.2x and *P* = 0.3), ruling out a systematic GC artifact as an explanation for our findings. We note that the CGCG motif is known to correlate with nucleosome occupancy^34,35^, which may potentially be expected since active promoters tend to have well-positioned nucleosomes marked by H3K4me3. Although the 5-mers CGCGC and CGGCG are too small to associate to known transcription factor binding motifs, we determined that the 9-mer GCGGTGGCT, which was enriched for heritability of blood-related traits in a previous study^36^ and is associated with the ZNF33A transcription factor binding motif, was enriched in the BasenjiΔ-H3K4me3-Max annotation (Supplementary Table 12).

As an alternative to conditional analysis using S-LDSC, we analyzed various sets of annotations by training a gradient boosting model to classify 12,296 SNPs from the NIH GWAS catalog^23^ and assessing the AUROC, as in ref. ^13,16^ (Methods); although this is not a formal conditional analysis, comparing the AUROC achieved by different sets of annotations can provide an indication of which annotations provide unique information for disease. Results are reported in Supplementary Table 13. We reached three main conclusions. First, the aggregated DeepSEAΔ and BasenjiΔ annotations were informative for disease (AUROC = 0.584 and 0.592, respectively, consistent with enrichments of these annotations (DeepSEAΔ: 1.50x, BasenjiΔ: 1.75x) for NIH GWAS SNPs; Supplementary Table 14). Second, including tissue-specific DeepSEAΔ and BasenjiΔ annotations for all 127 tissues slightly improved the results (AUROC = 0.602 and 0.611, respectively; lower than AUROC = 0.657 and 0.666 reported in ref. ^16^ because our analysis was restricted to chromatin marks and did not consider transcription factor binding site (TFBS) or cap analysis of gene expression (CAGE) data). Third, the disease informativeness of the baseline-LD model plus 7 non-tissue-specific annotations from Supplementary Fig. 6) (AUROC = 0.762) was not substantially impacted by adding the aggregated DeepSEAΔ and BasenjiΔ annotations (AUROC = 0.766 and 0.769, respectively). These findings were consistent with our S-LDSC analyses; in particular, the slightly higher AUROC for Basenji and DeepSEA allelic-effect annotations (across all analyses) was consistent with our S-LDSC results showing higher enrichments and a conditionally significant signal for Basenji annotations. Although a key limitation of the NIH GWAS catalog is that it consists predominantly of marginally associated variants that have not been fine-mapped, which thus form a noisy SNP set, these analyses show that it does contain useful signal.

We conclude that allelic-effect DeepSEA and Basenji annotations that were aggregated across tissues were enriched for heritability across the 41 traits (with higher enrichments for Basenji), and that one Basenji allelic-effect annotation was conditionally informative.

### Basenji brain-specific H3K4me3 is informative for disease

We evaluated the informativeness of blood-specific allelic-effect annotations across 11 blood-related traits (Supplementary Table 3), and the informativeness of brain-specific allelic-effect annotations across 8 brain-related traits (Supplementary Table 3).

As in the all-tissues analysis, we first evaluated tissue-specific variant-level annotations. The blood-specific variant-level DeepSEAV and BasenjiV annotations were highly enriched for heritability across 11 blood-related traits, but we determined that none of them were conditionally informative (Supplementary Figs. 8–11 and Supplementary Note). The brain-specific variant-level DeepSEAV and BasenjiV annotations were also highly enriched for heritability across 8 brain-related traits; surprisingly, two of these annotations (DeepSEAV-H3K4me3-brain-Max and BasenjiV-H3K27ac-brain-Max) were conditionally informative (Supplementary Figs. 12–15 and Supplementary Note). This is a surprising result, because the brain-specific variant-level deep learning annotations simply predict measured brain-specific variant-level annotations from Roadmap that were also included in the model and suggests unique information can be retrieved for brain tissues from de-noising of epigenomic signal using deep learning models. A possible reason for this may be poorer representation of brain tissues in the Roadmap data compared to the blood cell types.

We evaluated the informativeness of 8 blood-specific DeepSEAΔ and 8 blood-specific BasenjiΔ annotations (Table 1) for disease heritability by applying S-LDSC to the 11 blood-related traits. These analyses were conditioned on the the the baseline model plus 7 non-tissue-specific annotations from Supplementary Fig. 6, 6 blood-specific Roadmap and ChromHMM annotations from Supplementary Fig. 11 and BasenjiΔ-H3K4me3-Max (the 1 significant non-tissue-specific allelic-effect annotation; Fig. 2 and Supplementary Table 6).

A summary of the results is provided in Fig. 1 (Blood cell types, Blood traits column); numerical results in Supplementary Table 5. In our marginal analysis of disease heritability, all blood-specific allelic-effect annotations were enriched for disease heritability. Furthermore, blood-specific BasenjiΔ annotations were much more enriched for disease heritability (4.57x) than blood-specific DeepSEAΔ annotations (2.20x), despite similar annotation sizes (Supplementary Table 15). However, none of the blood-specific allelic-effect annotations attained a Bonferroni-significant standardized effect size (*τ*^⋆^) (Supplementary Table 15). (When we did not condition on the 11 conservation-related annotations of the baseline-LD model (Supplementary Table 9), this remained the case (Supplementary Table 16). In contrast, when we did not condition on BasenjiΔ-H3K4me3-Max, 0 blood-specific DeepSEAΔ annotations and 1 BasenjiΔ annotation attained a Bonferroni-significant *τ*^⋆^ (Supplementary Table 17); when we did not condition on BasenjiΔ-H3K4me3-Max or the 6 blood-specific annotations from Supplementary Fig. 11, 0 blood-specific DeepSEAΔ annotations and 6 blood-specific BasenjiΔ annotations attained a Bonferroni-significant *τ*^⋆^ (Supplementary Table 18).

We also analyzed various sets of blood-specific allelic-effect annotations by training a gradient boosting model to classify 8,741 fine-mapped autoimmune disease SNPs^24^ (relevant to blood-specific annotations only) and assessing the AUROC (analogous to Supplementary Table 13). Results are reported in Supplementary Table 19. We reached three main conclusions. First, the aggregated blood-specific DeepSEAΔ and BasenjiΔ annotations were informative for disease, with Basenji being more informative (AUROC = 0.613 and 0.672, respectively, consistent with moderate enrichments (DeepSEAΔ: 1.71x, BasenjiΔ: 2.37x) of these annotations for the fine-mapped SNPs; Supplementary Table 20). Second, including cell-type-specific allelic-effect DeepSEAΔ and BasenjiΔ annotations for all 27 blood cell types slightly improved the results (AUROC = 0.633 and 0.684, respectively). Third, the disease informativeness of the blood-specific variant-level joint model plus BasenjiΔ-H3K4me3-Max (AUROC = 0.848) was not substantially impacted by adding the aggregated blood-specific DeepSEAΔ and BasenjiΔ annotations (AUROC = 0.847 and 0.851, respectively). These findings were consistent with our S-LDSC analysis.

We evaluated the informativeness of 8 brain-specific DeepSEAΔ and 8 brain-specific BasenjiΔ annotations (Table 1) for disease heritability by applying S-LDSC to the 8 brain-related traits. These analyses were conditioned on the baseline-LD model plus 7 non-tissue-specific annotations from Supplementary Fig. 6, DeepSEAV-H3K4me3-brain-Max and BasenjiV-H3K27ac-brain-Max (the 2 significant brain-specific variant-level annotations; Supplementary Fig. 12) plus 4 additional brain-specific annotations from Supplementary Fig. 15 plus BasenjiΔ-H3K4me3-Max (the 1 significant non-tissue-specific allelic-effect annotation; Fig. 2 and Supplementary Table 6).

**Fig. 3:**
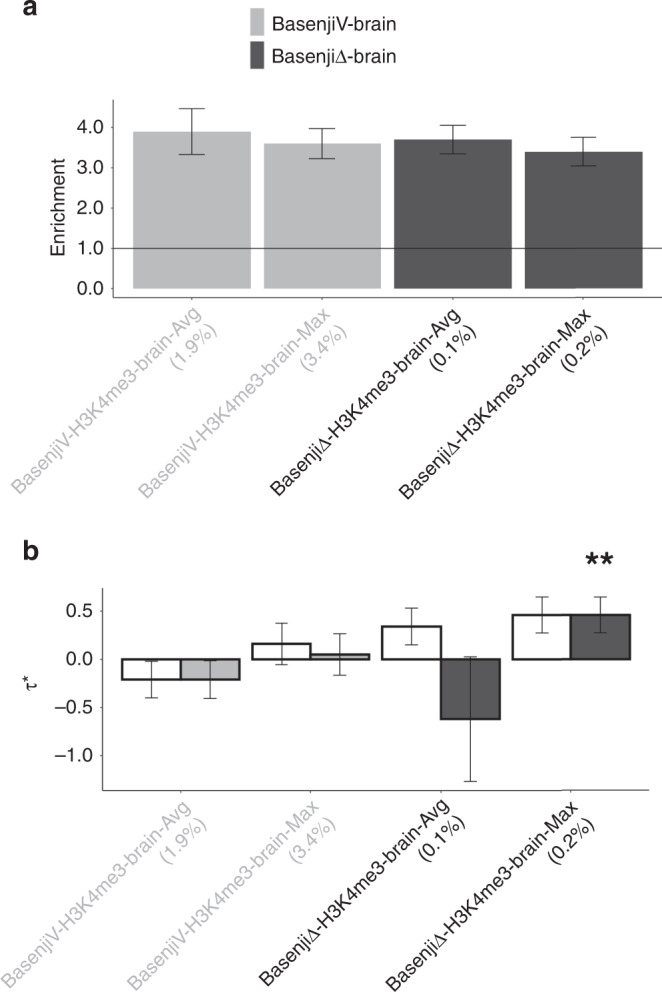
Disease informativeness of brain-specific allelic-effect deep learning annotations. **a** Heritability enrichment, conditioned on the brain-specific variant-level joint model and the 1 significant non-tissue-specific allelic-effect annotation (BasenjiΔ-H3K4me3-Max). Horizontal line denotes no enrichment. **b** Standardized effect size *τ*^⋆^ conditioned on either the brain-specific variant-level joint model and BasenjiΔ-H3K4me3-Max (marginal analysis: left column, white) or the same model plus 1 brain-specific allelic-effect annotation (BasenjiΔ-H3K4me3-brain-Max) (brain-specific final joint model: right column, dark shading). Results are meta-analyzed across 8 brain-related traits. Results are displayed only for the 2 allelic-effect annotations with significant τ^*^ in marginal analyses after correcting for 80 (variant-level + allelic-effect) brain-specific annotations tested (*P* < 0.05/80), along with the corresponding variant-level annotations; the correlation between the two allelic-effect annotations is 0.78, and the average correlation between the two pairs of variant-level (Basenji) and allelic-effect (BasenjiΔ) annotations is 0.44. For brain-specific final joint model (right column), **P < 0.05/80. Error bars denote 95% confidence intervals. Numerical results are reported in Supplementary Table 21 and Supplementary Table 27.

A summary of the results is provided in Fig. 1 (Brain tissues, Brain traits column); numerical results in Supplementary Table 5. In our marginal S-LDSC analysis, brain-specific BasenjiΔ annotations were more enriched for disease heritability (2.53x) than brain-specific DeepSEAΔ annotations (1.94x), despite similar annotation sizes. Two brain-specific BasenjiΔ annotations (BasenjiΔ-H3K4me3-brain-Max and BasenjiΔ-H3K4me3-brain-Avg) attained a Bonferroni-significant standardized effect size (*τ*^⋆^) (Fig. 3 and Supplementary Table 21). (When we did not condition on the 11 conservation-related annotations of the baseline-LD model (Supplementary Table 9), 8 brain-specific DeepSEAΔ and 6 brain-specific BasenjiΔ annotations attained a Bonferroni-significant *τ*^⋆^ (Supplementary Table 22). In addition, when we did not condition on BasenjiΔ-H3K4me3-Max, 0 brain-specific DeepSEAΔ annotations and 3 brain-specific BasenjiΔ annotations attained a Bonferroni-significant *τ*^⋆^ (Supplementary Table 23); when we did not condition on BasenjiΔ-H3K4me3-Max or the 6 brain-specific annotations from Supplementary Fig. 12 and Supplementary Fig. 15, 7 brain-specific DeepSEAΔ annotations and 7 brain-specific BasenjiΔ annotations attained a Bonferroni-significant *τ*^⋆^ (Supplementary Table 24).

Despite the high correlation between variant-level and allelic-effect annotations (*r* = 0.48; Supplementary Fig. 1), the corresponding variant-level annotations (BasenjiV-H3K4me3-brain-Max and BasenjiV-H3K4me3-brain-Avg) did not produce significant signal (Fig. 3 and Supplementary Table 25), consistent with our variant-level analysis (Supplementary Fig. 12). However, when we did not condition on these two variant-level annotations, 4 brain-specific DeepSEAΔ annotations and 6 brain-specific BasenjiΔ annotations attained a Bonferroni-significant *τ*^⋆^ (Supplementary Table 26).

We jointly analyzed the two annotations, BasenjiΔ-H3K4me3-brain-Max and BasenjiΔ-H3K4me3-brain-Avg, that were Bonferroni-significant in marginal analyses (Fig. 3) by performing forward stepwise elimination to iteratively remove annotations that had conditionally non-significant *τ*^⋆^ values after Bonferroni correction (based on the 80 variant-level and allelic-effect brain-specific annotations tested in marginal analyses). Of these, only BasenjiΔ-H3K4me3-brain-Max was jointly significant in the resulting brain-specific final joint model, with *τ*^⋆^ very close to 0.5 (Fig. 3, Supplementary Table 21 and Supplementary Table 27); annotations with *τ*^⋆^ ≥ 0.5 are unusual, and considered to be important^36^. A *k*-mer enrichment analysis (analogous to above) indicated that BasenjiΔ-H3K4me3-brain-Max was enriched for the *k*-mers CGCGC (6.2x and *P* = 1.1e-25) and CGGCG (6.1x and *P* = 4.9e-25) (far larger and more statistically significant than enrichments for simple GC-rich motifs such as the 2-mer CpG (1.4x and *P* = 0.32)), analogous to BasenjiΔ-H3K4me3-Max (Supplementary Table 11). The 9-mer GCGGTGGCT (which was enriched for heritability of blood-related traits in a previous study^36^, is associated with the ZNF33A transcription factor binding motif, and was enriched in the BasenjiΔ-H3K4me3-Max annotation; see above) was not enriched in the BasenjiΔ-H3K4me3-brain-Max annotation (Supplementary Table 12).

We did not consider secondary analyses of fine-mapped SNPs for brain-related traits, due to the lack of a suitable resource analogous to ref. ^24^.

We conclude that blood-specific allelic-effect annotations were very highly enriched for heritability but not uniquely informative for blood-related traits, whereas one brain-specific allelic-effect annotation was uniquely informative for brain-related traits. Blood-specific and brain-specific allelic-effect Basenji annotations generally outperformed DeepSEA annotations, yielding higher enrichments and the sole conditionally significant annotation, similar to our non-tissue-specific allelic-effect analyses.

## Discussion

We have evaluated the informativeness for disease of (variant-level and) allelic-effect annotations constructed using two previously trained deep learning models, DeepSEA^13,15^ and Basenji^16^. We evaluated each annotation’s informativeness using S-LDSC^5,19^; as a secondary metric, we also evaluated the accuracy of gradient boosting models incorporating deep learning annotations in predicting disease-associated or fine-mapped SNPs^23,24^, as in previous work^13,16^. In non-tissue-specific analyses, we identified one allelic-effect Basenji annotation that was uniquely informative for 41 diseases and complex traits. In blood-specific analyses, we identified no deep learning annotations that were uniquely informative for 11 blood-related traits. In brain-specific analyses, we identified brain-specific variant-level DeepSEA and Basenji annotations and a brain-specific allelic-effect Basenji annotation that were uniquely informative for 8 brain-related traits. We caution that-because we conditioned on a broad set of known functional annotations, in contrast to previous studies-the improvements provided by deep learning annotations were very small in magnitude, implying that further work is required to achieve the full potential of deep learning models for complex disease.

Our results imply that the informativeness of deep learning annotations for disease cannot be inferred from metrics such as AUROC that evaluate their accuracy in predicting underlying regulatory annotations derived from experimental assays. Instead, deep learning annotations must be evaluated using methods that specifically assess their informativeness for disease, conditional on a broad set of other functional annotations. The S-LDSC method that we applied here is one such method, and the accuracy of gradient boosting models incorporating both deep learning annotations and other functional annotations can also be a useful metric. We emphasize the importance of conditioning on a broad set of functional annotations, in order to assess whether deep learning models leveraging DNA sequence provide unique (as opposed to redundant) information. Previous work has robustly linked deep learning annotations to disease^12–16^, but those analyses did not condition on a broad set of other functional annotations.

Our work has several limitations, representing important directions for future research. First, our analyses of deep learning annotations using S-LDSC are inherently focused on common variants, but deep learning models have also shown promise in prioritizing rare pathogenic variants^15,37,38^. The value of deep learning models for prioritizing rare pathogenic variants has been questioned in a recent analysis focusing on Human Gene Mutation Database (HGMD) variants^39^, meriting further investigation. Second, our analyses of allelic-effect annotations are restricted to unsigned analyses, but signed analyses have also proven valuable in linking deep learning annotations to molecular traits and complex disease^16,40,41^. However, genome-wide signed relationships are unlikely to hold for the regulatory marks (DNase and histone marks) that we focus on here, which do not correspond to specific genes or pathways. Third, we focused here on deep learning models trained to predict specific regulatory marks, but deep learning models have also been used to predict a broader set of regulatory features, including gene expression levels and cryptic splicing^15,16,38^, that may be informative for complex disease. We have also not considered the application of deep learning models to TFBS, CAGE and ATAC-seq data^16,41^, which is a promising future research direction. Fourth, we focused here on deep learning models trained using human data, but models trained using data from other species may also be informative for human disease^41,42^. Fifth, the forward stepwise elimination procedure that we use to identify jointly significant annotations^19^ is a heuristic procedure whose choice of prioritized annotations may be close to arbitrary in the case of highly correlated annotations. Nonetheless, our framework does impose rigorous criteria for conditional informativeness. Finally, beyond deep learning models, it is of high interest to evaluate other machine learning methods for predicting regulatory effects^43–47^.

## Methods

### Genomic annotations and the baseline-LD model

We define a functional annotation as an assignment of a numeric value to each SNP; annotations can be either binary or continuous-valued (Methods). Our focus is on continuous-valued annotations (with values between 0 and 1) trained by deep learning models to predict biological function from DNA sequence. We define a genomic annotation as an assignment of a numeric value to each SNP in a predefined reference panel (e.g., 1000 Genomes Project^25^; see Data availability). Continuous-valued annotations can have any real value; our focus is on continuous-valued annotations with values between 0 and 1. Annotations that correspond to known or predicted function are referred to as functional annotations. The baseline-LD model (v.2.1) contains 86 functional annotations (see Data Availability). These annotations include binary coding, conserved, and regulatory annotations (e.g., promoter, enhancer, histone marks, TFBS) and continuous-valued linkage disequilibrium (LD)-related annotations.

### DeepSEA and Basenji annotations

Tissue-specific deep learning annotations were derived using two pre-trained Convolutional Neural Net (CNN) models: DeepSEA^13,15^ (architecture from ref. ^15^) and Basenji^16^ (see Code Availability). DeepSEA is a classification based model trained on binary peak call data from 2, 002 cell-type specific TFBS, histone mark and chromatin accessibility annotations from the ENCODE^21^ and Roadmap Epigenomics^11^ projects. Basenji is a Poisson likelihood model trained on original count data from 4, 229 cell-type specific histone mark, chromatin accessibility and FANTOM5 CAGE^48,49^ annotations. Additionally, Basenji uses dilated convolutional layers that allow scanning much larger contiguous sequence around a variant (≈130 kb) compared to DeepSEA (1 kb). We restricted our analyses to DNase-I Hypersensitivity Sites (DHS) and 3 histone marks (H3K27ac, H3K4me1 and H3K4me3) that are known to be associated with active enhancers and promoters^50^.

For each SNP with minor allele count ≥5 in 1000 Genomes, we applied the pre-trained DeepSEA and Basenji models to the surrounding DNA sequence (based on the reference allele) to compute the predicted probability of a tissue-specific chromatin mark (DNase, H3K27ac, H3K4me1, H3K4me3) to generate the corresponding variant-level annotation. To generate the corresponding allelic-effect annotation, we compute the predicted difference in probability between the reference and the alternate alleles. The Basenji annotations were quantile-matched to corresponding DeepSEA annotations to ensure a fair comparison of the two approaches. We aggregated these probabilistic annotations across all 127 Roadmap tissues by taking either the average (Avg) or maximum (Max) to generate non-tissue specific annotations, yielding 8 DeepSEA annotations and 8 Basenji annotations. Similarly, we aggregated over 27 blood cell types (respectively 13 brain tissues) to generate blood (respectively brain) specific annotations for each chromatin mark.

### BiClassCNN annotations

We trained a deep learning model, BiClassCNN, to prioritize SNPs within non-tissue-specific annotations; analyses of BiClassCNN annotations are described in the Supplementary Note. BiClassCNN analyzes 1kb of human reference sequence around each SNP (analogous to DeepSEA). The positive training set for BiClassCNN consists of 1kb of reference sequence around SNPs that are known to have the functionality of interest (e.g., coding); we included all such sequences in the positive training set. The negative training set consists of 1kb of reference sequence around SNPs that are 1kb away from all SNPs with the functionality of interest; we included a subset of such sequences in the negative training set, so as to match the overall size, GC content and repeat element content of the positive set (as in ref. ^43,51^). We used a shallow Convolutional Neural Net architecture for training (see Supplementary Fig. 16).

We ran two training models, one for the even chromosomes and one for odd chromosomes, and used the trained model on even (respectively odd) chromosomes to assign a predicted probability of functionality (e.g. coding), based on sequence context, to each SNP on odd (respectively even) chromosomes. Unlike DeepSEA and Basenji, BiClassCNN annotations were restricted to regions of known functionality (e.g., coding) by setting annotation values to 0 outside those regions; thus, BiClassCNN prioritizes SNPs within regions of known functionality (e.g., coding). (BiClassCNN annotations that were not restricted in this fashion were far less informative for disease.)

We restricted S-LDSC analyses of BiClassCNN annotations to annotations for which the BiClassCNN AUROC value was at least 0.6 (Table 1 and Supplementary Table 4). This eliminated three annotations (Intron, H3K27ac and UTR-3’), leaving a total of 12 BiClassCNN annotations.

### Other annotations

We also considered:

- (Supplementary Table 32) 8 Roadmap annotations^11^ (analogous to DeepSEA and Basenji annotations) imputed using ChromImpute^20^.
- (Supplementary Table 32) 40 ChromHMM annotations^21,22^ based on 20 ChromHMM states across 127 Roadmap tissues^11^, again aggregated using the average (Avg) or maximum (Max) across tissues.
- (Supplementary Table 33) 12 annotations consisting of CpG-island, local CpG-content and local GC-content annotations, as well as these annotations restricted to coding, repressed and TSS regions (for which BiClassCNN produced conditionally significant signals). The CpG-island annotation was retrieved from the UCSC genome browser^52^. Local CpG-content and local GC-content denote the proportion of *CpG* and *G* + *C* dinuclotides in  ±1 kb regions around each variant of the genome, computed using the hg19 reference genome fasta file. By definition, the LocalGCcontent annotation is of larger size than the LocalCpGcontent annotation.
- (Supplementary Table 33) 3 annotations consisting of a pLI annotation, as well as this annotation restricted to coding and TSS regions. The pLI annotation was defined by annotating each SNP in a 5 kb window around a gene with the pLI score of that gene^53^. We did not consider the pLI annotation restricted to repressed regions because unlike TSS and coding, repressed regions are not directly linked to a gene.
- (Supplementary Table 33) 2 coding annotations, SIFT^54^ and Polyphen^55,56^, which have been analyzed in previous work^57,58^.

### Stratified LD score regression

Stratified LD score regression (S-LDSC) is a method that assesses the contribution of a genomic annotation to disease and complex trait heritability^5,19^. Let *a*_*cj*_ be the value of annotation *c* for SNP *j*, where *a*_*cj*_ may be binary (0/1), continuous or probabilistic. S-LDSC assumes a linear model for **Y** on the normalized genotype matrix **X**:

1 $${{\bf{Y}}}_{{\bf{N}}\times {\bf{1}}}={{\bf{X}}}_{{\bf{N}}\times {\bf{M}}}{\beta }_{{\bf{M}}\times {\bf{1}}}+{\epsilon }_{{\bf{N}}\times {\bf{1}}},$$

where ${\boldsymbol{\beta }}=\left({\beta }_{1},{\beta }_{2},\cdots \ ,{\beta }_{M}\right)$ is the genotype effect size and *ϵ* denotes environmental noise. S-LDSC assumes that the per-SNP heritability for each SNP *j* can be decomposed as

2 $$var\left({\beta }_{j}\right):=\sum _{c}{a}_{cj}{\tau }_{c},$$

where *τ*_*c*_ is the per-SNP contribution of one unit of annotation *a*_*c*_ to heritability. Under this model assumption, the GWAS summary *χ*^2^ statistics can be linked to *τ*_*c*_ as follows:

3 $$E\left[{\chi }_{j}^{2}\right]=N\sum _{c}l(j,c){\tau }_{c}+1,$$

where $l(j,c)={\sum }_{k}{a}_{ck}{r}_{jk}^{2}$ is the *stratified LD score* of SNP *j* with respect to annotation *c* and *r*_*jk*_ is the genotypic correlation between SNPs *j* and *k*.

We assess the informativeness of an annotation *c* using two metrics. The first metric is enrichment (E), defined as follows (for binary and probabilistic annotations only):

4 $${{\rm{E}}}_{c}=\frac{\frac{{h}_{g}^{2}(c)}{{h}_{g}^{2}}}{\frac{\sum _{j}{a}_{cj}}{M}},$$

where ${h}_{g}^{2}(c)$ is the heritability explained by the SNPs in annotation *c*, weighted by the annotation values.

The second metric is standardized effect size (*τ*^⋆^) defined as follows (for binary, probabilistic, and continuous-valued annotations):

5 $${\tau }_{c}^{\star }=\frac{{\tau }_{c}s{d}_{c}}{\frac{{h}_{g}^{2}}{M}},$$

where *sd*_*c*_ is the standard error of annotation *c*, ${h}_{g}^{2}$ the total SNP heritability and *M* is the total number of SNPs on which this heritability is computed (equal to 5, 961, 159 in our analyses). ${\tau }_{c}^{\star }$ represents the proportionate change in per-SNP heritability associated to a 1 standard deviation increase in the value of the annotation. The main difference between enrichment and *τ*^⋆^ is that ${\tau }_{c}^{\star }$ quantifies effects that are unique to the focal annotation *c* (after conditioning on all other annotations), whereas enrichment quantifies effects that are unique and/or non-unique to the focal annotation. We computed the statistical significance (p-values) of the enrichment and *τ*^⋆^ of each annotation via block-jackknife over 200 blocks^5^; for *τ*^⋆^, we assumed that $\frac{{\tau }^{\star }}{se({\tau }^{\star })} \sim N(0,1)$.

### Weighted *k*-mer enrichment analysis

We performed weighted *k*-mer enrichment analyses of the deep learning annotations that were conditionally informative for disease heritability, for all 682 possible *k*-mers with 1 ≤ *k* ≤ 5 (merged with their reverse complements). Results of these analyses are reported in Supplementary Table 11 and Supplementary Table 50.

For each *k*-mer *i*, we computed *k*-mer counts ${\kappa }_{{\rm{s}}}^{({\rm{i}})}$ in the 1kb regions around each SNP *s* in the genome.

For each deep learning annotation **D**, for each *k*-mer *i*, we computed the weighted average ${{\rm{W}}}_{{\rm{D}}}^{({\rm{i}})}$ of *k*-mer counts *κ*^(i)^, weighted by values of the probabilistic annotation:

6 $${{\rm{W}}}_{{\rm{D}}}^{({\rm{i}})}:=\sum _{s}{D}_{{\rm{s}}}{\kappa }_{{\rm{s}}}^{({\rm{i}})}.$$

We compared ${{\rm{W}}}_{{\rm{D}}}^{({\rm{i}})}$ with ${{\rm{W}}}_{{{\rm{D}}}^{\text{null}}}^{({\rm{i}})}$, where **D**^null^ is defined as the probabilistic annotation with all values uniformly equal to $\bar{D}$, the average value (annotation size) of annotation **D**.

We computed the *weighted k*-*mer enrichment* of annotation *D* with respect to *k*-mer *i* as

7 $${{\rm{WKE}}}_{{\rm{D}}}^{({\rm{i}})}:={{\rm{W}}}_{{\rm{D}}}^{({\rm{i}})}/{{\rm{W}}}_{{{\rm{D}}}^{\text{null}}}^{({\rm{i}})}$$

We assessed the statistical significance of the weighted *k*-mer enrichment via a permutation test in which we randomly permuted the values of the deep learning annotation *D* across SNPs and compared ${{\rm{WKE}}}_{{\rm{D}}}^{({\rm{i}})}$ to values of ${{\rm{WKE}}}_{{{\rm{D}}}^{\text{perm}}}^{({\rm{i}})}$ for each permuted annotation D^perm^. We computed p-values by fitting a Gaussian distribution to the values of ${{\rm{WKE}}}_{{{\rm{D}}}^{\text{perm}}}^{({\rm{i}})}$ across 10,000 such permutations.

### Classification of disease-associated or fine-mapped SNPs

As an alternative to conditional analysis using S-LDSC, we evaluated the efficacy of various sets of annotations for classifying 12,296 disease-associated SNPs from the NIH GWAS catalog^23^ (as in refs. ^13,16^) or 8,741 fine-mapped autoimmune disease SNPs^24^ against the same number of control SNPs, matched for minor allele frequency. We used XGBoost, a machine learning technique based on gradient tree boosting^59,60^. To optimize classification performance, we selected XGBoost parameter settings to minimize overfitting, as in refs. ^61^^62^,^63^.

### Reporting summary

Further information on research design is available in the Nature Research Reporting Summary linked to this article.

## Supplementary information

Supplementary Information

Peer Review File

Reporting Summary

## Data Availability

All deep learning annotations and other annotations used in this paper as well as relevant codes are available online at https://data.broadinstitute.org/alkesgroup/LDSCORE/DeepLearning/. This work used summary statistics from the UK Biobank study (http://www.ukbiobank.ac.uk/). The summary statistics for UK Biobank used in this paper are available at https://data.broadinstitute.org/alkesgroup/UKBB/. The 1000 Genomes Project Phase 3 data are available at ftp://ftp.1000genomes.ebi.ac.uk/vol1/ftp/release/20130502. The baseline-LD annotations are available at https://data.broadinstitute.org/alkesgroup/LDSCORE/.

## Code availability

This work primarily uses the S-LDSC software (https://github.com/bulik/ldsc). We used publicly available software for DeepSEA (https://github.com/FunctionLab/ExPecto) and Basenji (https://github.com/calico/basenji) to generate annotations for these respective models. Codes for training and evaluating the BiClassCNN model are provided here: https://data.broadinstitute.org/alkesgroup/LDSCORE/DeepLearning/.
