# Supplementary material for: Evaluating the informativeness of deep learning annotations for human complex diseases
Source: Nat Commun. 2020 Sep 17;11:4703. doi: 10.1038/s41467-020-18515-4 (PMC7499261; doi:10.1038/s41467-020-18515-4)
Supplement: Supplementary file 1 — Supplementary Information [file 41467_2020_18515_MOESM1_ESM.pdf]

Supplementary Information for Dey et al.: Evaluating the informativeness of deep learning annotations for human complex diseases

Supplementary Note

Overview of analysis of variant-level annotations

In this supplementary note, we perform disease heritability analysis of 25 variant-level deep learning annotations from three models (8 DeepSEAV, 8 BasenjiV and 9 BiClassCNN), as well as other variant-level epigenomic annotations such as 8 Roadmap annotations[1] (analogous to DeepSEAV and BasenjiV annotations), imputed using ChromImpute[2]; and 40 ChromHMM annotations[3,4] based on 20 ChromHMM states across 127 Roadmap tissues[1] (Supplementary Table 2). The variant-level deep learning annotations can be treated as a de-noised representation of Roadmap data learned from sequence architecture. All these annotations contributed to the conditional model in the analysis of the allelic-effect deep learning annotations in the main text. All analyses of variant-level annotations were conditional on a broad set of coding, conserved, regulatory and LD-related annotations in our analyses: 86 annotations from the baseline-LD (v2.1) model[5]. In our analysis of 11 blood-related traits (respectively 8 brain-related traits), for variant-level annotations, we analyzed 16 variant-level annotations (8 DeepSEAV and 8 BasenjiV) that were aggregated across 27 blood cell types (respectively 13 brain tissues), instead of all 127 tissues.

We calculated the variant-level DeepSEAV annotations by applying previously trained DeepSEA models[6] (see Code Availability) for each of 4 tissue-specific chromatin marks (DNase, H3K27ac, H3K4me1, H3K4me3) across 127 Roadmap tissues[1] to 1kb of human reference sequence around each SNP ad calculating the predicted occurrence of the mark based on the sequence (with respect to the reference allele); for each chromatin mark, we aggregated allelic-effect DeepSEA annotations across the 127 tissues using either the average (Avg) or maximum (Max) across tissues (Table 1 and Methods). Similarly, we calculated the variant-level BasenjiV annotations by applying previously trained Basenji models[7] (see Code Availability) and aggregating across tissues in analogous fashion (Table 1 and Methods). The constituent tissue-specific BasenjiV annotations do not lie between 0 and 1, and ref.[7] did not report AUROC values; we transformed these annotations to lie between 0 and 1 via quantile matching with corresponding DeepSEAV annotations, to ensure a fair comparison of the two approaches (Methods). When comparing pairs of annotations that differed only in their aggregation strategy (Avg/Max), chromatin mark (DNase/H3K27ac/H3K4me1/H3K4me3), model (DeepSEA/Basenji) or type (variant-level/allelic-effect), respectively, we observed large correlations across aggregation strategies (average $r = 0.71$), chromatin marks (average $r = 0.58$), models (average $r = 0.54$) and types (average $r = 0.48$) (Supplementary Figure 1).

Both DeepSEA and Basenji models were trained on tissue-specific features and these features were then aggregated across tissues; as an alternative, we performed a new Convolutional Neural Net training of sequences that separates out non-tissue-specific functional features from putatively non-functional features (Supplementary Figure 16). We constructed 9 annotations from a new CNN model called BiClassCNN that separates non-tissue-specific functional regions from potentially non-functional regions for different functional categories (Table 1 and Methods); unlike variant-level DeepSEAV and BasenjiV annotations, the BiClassCNN model was only applied to non-tissue-specific annotations (e.g. coding, promoter, or TSS), and was used to prioritize SNPs within these annotations by restricting BiClassCNN predictions to the underlying annotations; prior to this restriction, the BiClassCNN model was highly predictive of the underlying annotations, with AUROC values ranging from $0.67 - 0.84$

(Supplementary Table 4).

No all-tissues variant-level annotation is conditionally informative for disease

A summary of the results is provided in Supplementary Figure 17 (All tissues, All traits column; numerical results in Supplementary Table 28), which reports the number of variant-level annotations of various types with significant heritability enrichment, marginal conditional signal, and joint conditional signal, respectively. In our marginal analysis of disease heritability using S-LDSC, all DeepSEAV annotations were highly enriched for disease heritability (average enrichment 2.2x), but only one DeepSEAV annotation (DeepSEAV-H3K4me3-Max) attained a Bonferroni-significant standardized effect size ($\tau^{\star}$) conditional on the baseline-LD model (Supplementary Figure 2 and Supplementary Table 29). This implies that high AUROC values for variant-level annotations do not necessarily translate into conditional informativeness for human disease. In our marginal analysis of disease heritability using S-LDSC, all BasenjiV annotations were highly enriched for disease heritability (average enrichment 2.2x), but no BasenjiV annotation attained a Bonferroni-significant $\tau^{\star}$ conditional on the baseline-LD model (Supplementary Table 29). In our marginal analysis of disease heritability using S-LDSC, all BiClassCNN annotations were highly enriched for disease heritability (average enrichment 2.9x) except for BiClassCNN-Repressed (which was depleted, 0.68x), and 4 BiClassCNN annotations attained a Bonferroni-significant $\tau^{\star}$ conditional on the baseline-LD model (Supplementary Figure 2 and Supplementary Table 30). 3 of these 4 annotations (BiClassCNN-Coding, BiClassCNN-Repressed, and BiClassCNN-TSS) produced independent signals (Supplementary Table 31).

To ensure a conservative assessment of which annotations provide unique information for disease, we included a broader set of annotations in our analyses. First, we included 8 Roadmap annotations[1] (analogous to DeepSEAV and BasenjiV annotations) imputed using ChromImpute[2], and 40 ChromHMM annotations[3,4] based on 20 ChromHMM states across 127 Roadmap tissues[1], again aggregated using the average (Avg) or maximum (Max) across tissues (Supplementary Table 2 and Methods). Of these, 4 Roadmap annotations and 17 ChromHMM annotations attained a Bonferroni-significant $\tau^{\star}$ conditional on the baseline-LD model (Supplementary Figure 3, Supplementary Figure 4 and Supplementary Table 32). Second, motivated by the BiClassCNN results (see above), we investigated additional annotations related to coding, repressed and TSS regions. We determined that BiClassCNN-TSS was highly correlated with CpG-island, local CpG-content (±1kb) and local GC-content (±1kb) annotations (Supplementary Figure 5). We thus incorporated CpG-island, local CpG-content and local GC-content annotations, as well as these annotations restricted to coding, repressed and TSS regions. We also considered a gene-level annotation (±5kb) based on probability of loss-of-function intolerance (pLI)[8], the pLI annotation restricted to coding and TSS regions, and PolyPhen[9,10] and SIFT[11] coding annotations ($12 + 5 = 17$ additional annotations; Supplementary Table 33 and Methods). 11 of these 17 annotations attained a Bonferroni-significant $\tau^{\star}$ conditional on the baseline-LD model (Supplementary Table 33).

We jointly analyzed all 38 annotations that were Bonferroni-significant in our marginal analyses (Supplementary Figure 2, Supplementary Figure 3, Supplementary Figure 4, Supplementary Table 29, Supplementary Table 30, Supplementary Table 32, Supplementary Table 33) by performing forward stepwise elimination to iteratively re-

move annotations that had conditionally non-significant τ^* values after Bonferroni correction (based on a total of 106 (variant-level + allelic-effect) non-tissue-specific annotations tested in marginal analyses). Of these, 0 DeepSEAV, 0 BiClassCNN, 1 Roadmap, 1 ChromHMM, 3 local GC-content and 2 pLI annotations were jointly significant in the resulting joint model (Supplementary Figure 2, Supplementary Figure 6 and Supplementary Table 34); these annotations were included in all of the conditional analyses below. We determined that the surviving Roadmap-H3K4me1-Avg annotation was sufficient to eliminate the DeepSEAV-H3K4me3-Max annotation that was significant in our marginal analysis (Supplementary Table 35), and that the local GC-content annotations were sufficient to eliminate the 4 BiClassCNN annotations that were significant in our marginal analysis (Supplementary Table 36).

We also analyzed various sets of allelic-effect annotations by training a gradient boosting model to classify 12,296 SNPs from the NIH GWAS catalog[12] and assessing the AUROC (analogous to Supplementary Table 13), as in ref.[13,7] (Methods). Results are reported in Supplementary Table 37. We reached three main conclusions. First, the aggregated variant-level DeepSEAV and BasenjiV annotations were only moderately informative for disease (AUROC = 0.582 and 0.612, respectively, consistent with moderate enrichments (DeepSEAV: 1.49x, BasenjiV: 1.49x) of these annotations for NIH GWAS SNPs; Supplementary Table 38). Second, including tissue-specific variant-level DeepSEAV and BasenjiV annotations for all 127 tissues had little impact on the results (AUROC = 0.591 and 0.620, respectively). Third, the disease informativeness of the baseline-LD model (AUROC = 0.758) was not substantially impacted by adding the aggregated variant-level DeepSEAV and BasenjiV annotations (AUROC = 0.759 and 0.766, respectively); results were similar for the non-tissue-specific joint model (baseline-LD model + 7 annotations). These findings were consistent with our S-LDSC analyses.

We conclude that variant-level DeepSEAV and BasenjiV annotations that were aggregated across tissues were highly enriched for heritability but not conditionally informative across the 41 traits, and that marginally significantly informative BiClassCNN annotations were explained away by local GC-content.

DeepSEA and Basenji brain-specific variant-level annotations are conditionally informative for disease

We next evaluated the informativeness of blood-specific variant-level annotations for blood-related diseases and traits. We analyzed 11 independent blood-related traits (6 autoimmune diseases and 5 blood cell traits; ref.[14] and Supplementary Table 3) by running S-LDSC conditioned on the non-tissue-specific variant-level joint model (baseline-LD model + 7 annotations; Supplementary Figure 6).

We analyzed 8 blood-specific variant-level DeepSEAV and 8 blood-specific variant-level BasenjiV annotations (Supplementary Table 39), representing the blood-specific analogues of the non-tissue-specific variant-level DeepSEAV and BasenjiV annotations from Table 1; in each case we computed the Average (Avg) or Maximum (Max) of cell-type-specific DeepSEAV and BasenjiV variant-level annotations across blood cell types. A summary of the results is provided in Supplementary Figure 17 (Blood cell types, Blood traits column); numerical results in Supplementary Table 28. In our marginal S-LDSC analysis, both blood-specific BasenjiV annotations and blood-specific DeepSEAV annotations were very highly enriched (average enrichment 4.15x

and 3.75x, respectively). However, only 0 blood-specific DeepSEAV annotations and 4 blood-specific BasenjiV annotations attained a Bonferroni-significant standardized effect size ($\tau^{\star}$) conditional on the non-tissue-specific variant-level joint model (Supplementary Figure 8 and Supplementary Table 39).

We also analyzed 8 blood-specific Roadmap and 40 blood-specific ChromHMM annotations (Supplementary Table 40), representing the blood-specific analogues of the non-tissue-specific Roadmap and ChromHMM annotations from Supplementary Table 2. In our marginal S-LDSC analysis, all 8 blood-specific Roadmap annotations and 23 blood-specific ChromHMM annotations attained a Bonferroni-significant $\tau^{\star}$ (Supplementary Figure 9, Supplementary Figure 10 and Supplementary Table 40).

We jointly analyzed the 35 blood-specific annotations that were Bonferroni-significant in marginal analyses (4 BasenjiV, 8 Roadmap, 23 ChromHMM; Supplementary Figure 8, Supplementary Figure 9 and Supplementary Figure 10) by performing forward stepwise elimination to iteratively remove annotations that had conditionally non-significant $\tau^{\star}$ values after Bonferroni correction (based on the 80 (variant-level + allelic effect) blood-specific annotations tested in marginal analyses). Of these, 0 BasenjiV, 2 Roadmap and 4 ChromHMM annotations were jointly significant in the resulting joint model (Supplementary Figure 8, Supplementary Figure 11 and Supplementary Table 41). We determined that the 2 surviving blood-specific Roadmap annotations were sufficient to eliminate 3 of the 4 blood-specific BasenjiV annotations that were significant in marginal analyses (Supplementary Table 42). We note that several of the blood-specific Roadmap and ChromHMM annotations had $\tau^{\star} > 0.5$ in the blood-specific joint model (Supplementary Figure 11) (annotations with $\tau^{\star} > 0.5$ are unusual, and considered to be important[15]), consistent with the well-documented importance of tissue-specific annotations, particularly for blood-related traits[16,1].

We also analyzed various sets of blood-specific annotations by training a gradient boosting model to classify 8,741 fine-mapped autoimmune disease SNPs[17] (relevant to blood-specific annotations only) and assessing the AUROC (analogous to Supplementary Table 37). Results are reported in Supplementary Table 43. We reached three main conclusions. First, the aggregated blood-specific variant-level DeepSEAV and BasenjiV annotations were informative for disease, with BasenjiV being more informative (AUROC = 0.621 and 0.664, respectively, consistent with moderate enrichments (DeepSEAV: 1.75x, BasenjiV: 1.97x) of these annotations for the fine-mapped SNPs; Supplementary Table 44). Second, including cell-type-specific variant-level DeepSEAV and BasenjiV annotations for all 27 blood cell types had little impact on the results (AUROC = 0.631 and 0.671, respectively). Third, the disease informativeness of the non-tissue-specific joint model (AUROC = 0.845) was not substantially impacted by adding aggregated blood-specific DeepSEAV and BasenjiV variant-level annotations (AUROC = 0.849 and 0.853, respectively); results were similar for the the blood-specific joint model (non-tissue-specific joint model + 6 blood annotations). These findings were consistent with our S-LDSC analyses.

We next evaluated the informativeness of brain-specific variant-level annotations for brain-related diseases and traits, analogous to blood. We analyzed 8 independent brain-related traits (ref.[14] and Supplementary Table 3) by running S-LDSC conditioned on the non-tissue-specific variant-level joint model (baseline-LD model + 7 annotations; Supplementary Figure 6).

We analyzed 8 brain-specific variant-level DeepSEAV and 8 brain-specific variant-level BasenjiV annotations (Supplementary Table 45), representing the brain-specific ana-

logues of the non-tissue-specific variant-level DeepSEAV and BasenjiV annotations from Table 1; in each case we computed the Average (Avg) or Maximum (Max) of tissue-type-specific DeepSEAV and BasenjiV variant-level annotations across brain tissues. A summary of the results is provided in Supplementary Figure 17 (Brain tissues, Brain traits column); numerical results in Supplementary Table 28. In our marginal S-LDSC analysis, both brain-specific DeepSEAV and BasenjiV annotations were highly enriched (average enrichment 2.71x and 2.73x, respectively; Supplementary Table 45). Furthermore, all 8 brain-specific DeepSEAV annotations and all 8 brain-specific BasenjiV annotations attained a Bonferroni-significant standardized effect size ($\tau^\star$) conditional on the non-tissue-specific variant-level joint model (Supplementary Figure 12 and Supplementary Table 45).

We also analyzed 8 brain-specific Roadmap and 40 brain-specific ChromHMM annotations (Supplementary Table 46), representing the brain-specific analogues of the non-tissue-specific Roadmap and ChromHMM annotations from Supplementary Table 2. In our marginal S-LDSC analysis, all 8 brain-specific Roadmap annotations and 13 brain-specific ChromHMM annotations attained a Bonferroni-significant τ^* (Supplementary Figure 13, Supplementary Figure 14 and Supplementary Table 46).

We jointly analyzed the 37 brain-specific annotations that were Bonferroni-significant in marginal analyses (8 DeepSEAV, 8 BasenjiV, 8 Roadmap, 13 ChromHMM; Supplementary Figure 12, Supplementary Figure 13 and Supplementary Figure 14) by performing forward stepwise elimination to iteratively remove annotations that had conditionally non-significant $\tau^\star$ values after Bonferroni correction (based on the 80 (variant-level + allelic-effect) brain-specific annotations tested in marginal analyses). Of these, 1 DeepSEAV, 1 BasenjiV, 1 Roadmap and 3 ChromHMM annotations were jointly significant in the resulting joint model (Supplementary Figure 12, Supplementary Figure 15 and Supplementary Table 47). We determined that neither the 1 surviving brain-specific Roadmap annotation nor the 3 surviving brain-specific ChromHMM annotations were sufficient to eliminate any of the 8 DeepSEAV and 8 BasenjiV brain annotations that were significant in marginal analyses (Supplementary Table 48 and Supplementary Table 49). We note that none of the brain-specific DeepSEAV, BasenjiV, Roadmap and ChromHMM annotations had $\tau^\star > 0.5$ in the brain-specific joint model (Supplementary Figure 12 and Supplementary Figure 15). We did not consider secondary analyses of fine-mapped SNPs for brain-related traits, due to the lack of a suitable resource analogous to ref.[17].

We conclude that blood-specific variant-level DeepSEAV and BasenjiV annotations that were aggregated across blood cell types were very highly enriched for heritability but not uniquely informative for blood-related traits. On the other hand, brain-specific variant-level DeepSEAV and BasenjiV annotations that were aggregated across brain tissues were uniquely informative for brain-related traits. This may be because brain tissues are not as well-represented in Roadmap data as blood cell types, leaving more room for new information to be retrieved by deep learning models. This also justifies that though allelic-effect annotations should definitely be the annotations of primary interest, variant-level annotations may also be of some value, and should be conditioned on.

Supplementary Tables

Supplementary Table 1. **List of non-tissue-specific variant-level deep learning annotations analyzed.** We list the 25 variant-level deep learning annotations (8 DeepSEAV, 8 BasenjiV, 9 BiClassCNN) and their annotation sizes.

Variant-level annotations	Size (%)
DeepSEAV-DNase-Avg	1.6
DeepSEAV-DNase-Max	13.2
DeepSEAV-H3K27ac-Avg	3.2
DeepSEAV-H3K27ac-Max	13.7
DeepSEAV-H3K4me1-Avg	5.5
DeepSEAV-H3K4me1-Max	23.2
DeepSEAV-H3K4me3-Avg	1.7
DeepSEAV-H3K4me3-Max	8.8
BasenjiV-DNase-Avg	1.6
BasenjiV-DNase-Max	13.1
BasenjiV-H3K27ac-Avg	3.2
BasenjiV-H3K27ac-Max	13.7
BasenjiV-H3K4me1-Avg	5.5
BasenjiV-H3K4me1-Max	23.2
BasenjiV-H3K4me3-Avg	1.7
BasenjiV-H3K4me3-Max	8.8
BiClassCNN-Coding	1.0
BiClassCNN-Enhancer	2.6
BiClassCNN-Promoter	2.7
BiClassCNN-Repressed	36.0
BiClassCNN-SuperEnhancer	6.7
BiClassCNN-TFBS	7.9
BiClassCNN-TSS	0.8
BiClassCNN-UTR-5'	0.4
BiClassCNN-WeakEnhancer	1.1

Supplementary Table 2. List of non-tissue-specific Roadmap and ChromHMM annotations analyzed. We list the 8 Roadmap and 40 ChromHMM annotations that we analyzed, together with the size of the annotation. These annotations are probabilistic, thus the size of the annotation is defined as the average annotation across all reference SNPs.

Annotation	Size (in %)
Roadmap-DNase-Avg	2.2%
Roadmap-H3K27ac-Avg	3.1%
Roadmap-H3K4me1-Avg	4.4%
Roadmap-H3K4me3-Avg	1.7%
Roadmap-DNase-Max	13.4%
Roadmap-H3K27ac-Max	21.4%
Roadmap-H3K4me1-Max	25.4%
Roadmap-H3K4me3-Max	6.6%
ChromHMM-DNase-Avg	0.7%
ChromHMM-Active_Enhancer_1-Avg	0.3%
ChromHMM-Active_Enhancer_2-Avg	0.4%
ChromHMM-Enhancer_acetylation-Avg	0.3%
ChromHMM-Active_Enhancer_Flanking-Avg	0.5%
ChromHMM-Weak_Enhancer_1-Avg	0.3%
ChromHMM-Weak_Enhancer_2-Avg	1.2%
ChromHMM-Heterochromatin-Avg	1.4%
ChromHMM-Promoter_Bivalent-Avg	0.3%
ChromHMM-Promoter_Downstream_1-Avg	0.4%
ChromHMM-Promoter_Downstream_2-Avg	0.2%
ChromHMM-Promoter_Poised-Avg	0.2%
ChromHMM-Promoter_Upstream-Avg	0.4%
ChromHMM-Quiescent-Avg	78.4%
ChromHMM-Repressed_Polycomb-Avg	1.7%
ChromHMM-TSS-Avg	0.2%
ChromHMM-Transcription_Enhancer_3'-Avg	0.2%
ChromHMM-Transcription_Enhancer_5'-Avg	0.4%
ChromHMM-Transcription_Weak_Enhancer-Avg	0.5%
ChromHMM-Transcriptional_Regulator-Avg	0.3%
ChromHMM-DNase-Max	9.2%
ChromHMM-Active_Enhancer_1-Max	7.1%
ChromHMM-Active_Enhancer_2-Max	9.9%
ChromHMM-Enhancer_acetylation-Max	11.3%
ChromHMM-Active_Enhancer_Flanking-Max	11.1%
ChromHMM-Weak_Enhancer_1-Max	7.7%
ChromHMM-Weak_Enhancer_2-Max	19.9%
ChromHMM-Heterochromatin-Max	7.1%
ChromHMM-Promoter_Bivalent-Max	1.9%
ChromHMM-Promoter_Downstream_1-Max	1.4%
ChromHMM-Promoter_Downstream_2-Max	1.5%
ChromHMM-Promoter_Poised-Max	6.7%
ChromHMM-Promoter_Upstream-Max	2.5%
ChromHMM-Quiescent-Max	91.0%
ChromHMM-Repressed_Polycomb-Max	9.9%
ChromHMM-TSS-Max	0.9%
ChromHMM-Transcription_Enhancer_3'-Max	3.1%
ChromHMM-Transcription_Enhancer_5'-Max	4.8%
ChromHMM-Transcription_Weak_Enhancer-Max	4.7%
ChromHMM-Transcriptional_Regulator-Max	3.3%

Supplementary Table 3. List of 41 diseases and complex traits analyzed. We first list the 11 blood-related traits (6 autoimmune diseases and 5 blood cell traits), followed by 10 brain traits (with 2 traits analyzed by two different datasets). Overall, for 6 traits we analyzed two different data sets, leading to a total of 47 data sets.

Trait	Source	N
Platelet Count	UKBiobank[18]	444382
Red Blood Cell Count	UKBiobank[18]	445174
Red Blood Cell Distribution Width	UKBiobank[18]	442700
Eosinophil Count	UKBiobank[18]	439938
White Blood Cell Count	UKBiobank[18]	444502
Auto Immune Traits (Sure)	UKBiobank[18]	459324
Crohn's Disease	Jostins et al., 2012 Nature[19]	20883
Rheumatoid Arthritis	Okada et al., 2014 Nature[20]	37681
Ulcerative Colitis	Jostins et al., 2012 Nature[19]	27432
Lupus	Bentham et al., 2015[21]	14267
Celiac	Dubois et al., 2010[22]	15283
Age at Menarche	UKBiobank[18]	242278
BMI	Speliotes et al., 2010 Nat Genet[23]	122033
BMI	UKBiobank[18]	457824
Depressive symptoms	Okbay et al., 2016 Nat Genet[24]	161460
Neuroticism	UKBiobank[18]	372066
Schizophrenia	SCZ Working Group of the PGC, 2014 Nature[25]	70100
Years of Education	Okbay et al., 2016 Nature[24]	328917
Ever Smoked	TAG Consortium, 2010 Nat Genet[26]	74035
Smoking Status	UKBiobank[18]	457683
Bipolar Disorder	PGC Bipolar Disorder Group[27]	16731
Age at Menopause	UKBiobank[18]	143025
Age first birth	Barban et al., 2016 Nat Genet[28]	222037
Anorexia	Boraska et al., 2014 Mol Psych[29]	32143
Autism Spectrum	PGC Cross-Disorder Group, 2013 Lancet[30]	10263
College Education	UKBiobank[18]	454813
Coronary Artery Disease	Schunkert et al., 2011 Nat Genet[31]	77210
Dermatologic Diseases	UKBiobank[18]	459324
Eczema	UKBiobank[18]	458699
FEV1-FVC Ratio	UKBiobank[18]	371949
Forced Vital Capacity (FVC)	UKBiobank[18]	371949
Hair Color	UKBiobank[18]	452720
HDL	Teslovich et al., 2010 Nature[32]	97749
Heel T Score	UKBiobank[18]	445921
Height	Lango Allen et al., 2010 Nature[33]	131547
Height	UKBiobank[18]	458303
High Cholesterol	UKBiobank[18]	459324
Hypothyroidism	UKBiobank[18]	459324
LDL	Teslovich et al., 2010 Nature[32]	93354
Morning Person	UKBiobank[18]	410520
Number children ever born	Barban et al., 2016 Nat Genet[28]	318863
Respiratory and Ear-nose-throat Diseases	UKBiobank[18]	459324
Skin Color	UKBiobank[18]	453609
Sunburn Occasion	UKBiobank[18]	344229
Tanning	UKBiobank[18]	449984
Type 2 Diabetes	Morris et al., 2012 Nat Genet[34]	60786
Type 2 Diabetes	UKBiobank[18]	459324

Supplementary Table 4. **AUROC values of deep learning annotations**. AUROC (Area under the ROC curve) for DeepSEAV (reported by ref.[6]) and BiClassCNN annotations. For the DeepSEA model, the Minimum-Maximum (Average) AUROC across all tissues is reported. The constituent tissue-specific Basenji annotations do not lie on a probabilistic scale, and ref.[7] did not report AUROC values

Annotation	Area under ROC curve (AUROC)
DeepSEAV-DNase	All: 0.83-0.95 (0.91)<br>brain: 0.84-0.92 (0.88)<br>blood: 0.86-0.94 (0.89)
DeepSEAV-H3K27ac	All: 0.78-0.88 (0.84)<br>brain: 0.80-0.85 (0.82)<br>blood: 0.78-0.87 (0.83)
DeepSEAV-H3K4me1	All: 0.77-0.86 (0.81)<br>brain: 0.79-0.83 (0.80)<br>blood: 0.78-0.83 (0.81)
DeepSEAV-H3K4me3	All: 0.88-0.97 (0.92)<br>brain: 0.89-0.95 (0.91)<br>blood: 0.88-0.95 (0.92)
BiClassCNN-Coding	0.79
BiClassCNN-DNase	0.69
BiClassCNN-Enhancer	0.72
BiClassCNN-H3K4me1	0.70
BiClassCNN-H3K4me3	0.73
BiClassCNN-Promoter	0.76
BiClassCNN-Repressed	0.70
BiClassCNN-SuperEnhancer	0.67
BiClassCNN-TFBS	0.75
BiClassCNN-TSS	0.84
BiClassCNN-UTR-5'	0.75
BiClassCNN-WeakEnhancer	0.74

Supplementary Table 5. **Number of significantly disease informative non-tissue-specific allelic-effect deep learning annotations**. Number of significantly disease informative non-tissue-specific allelic-effect annotations across different aggregation strategies (A) different deep learning models (DeepSEA and Basenji), (B) different aggregation strategies (Average and Maximum) and (C) different types of epigenomic marks, in terms of marginal enrichment in heritability, conditional $\tau^{\star}$ and joint $\tau^{\star}$ for all traits, blood traits and brain traits respectively. See Figure 1 for visual illustration. N.annot in brackets represents the number of significant annotations.

PANEL A

Enrichment

Model	Aggr. Strategy (N.annot)
DeepSEAΔ	All tissues, All traits (8), Brain tissues, Brain traits (8), Blood cell types, Blood traits (8)
BasenjiΔ	All tissues, All traits (8), Brain tissues, Brain traits (8), Blood cell types, Blood traits (8)

Marginal $\tau^{\star}$

Model	Aggr. Strategy (N.annot)
DeepSEAΔ	All tissues, All traits (0), Brain tissues, Brain traits (0), Blood cell types, Blood traits (0)
BasenjiΔ	All tissues, All traits (1), Brain tissues, Brain traits (2), Blood cell types, Blood traits (0)

Joint $\tau^{\star}$

Model	Aggr. Strategy (N.annot)
DeepSEAΔ	All tissues, All traits (0), Brain tissues, Brain traits (0), Blood cell types, Blood traits (0)
BasenjiΔ	All tissues, All traits (1), Brain tissues, Brain traits (1), Blood cell types, Blood traits (0)

PANEL B

Enrichment

Model	Aggr. Strategy (N.annot)
Avg	All tissues, All traits (8), Brain tissues, Brain traits (8), Blood cell types, Blood traits (8)
Max	All tissues, All traits (8), Brain tissues, Brain traits (8), Blood cell types, Blood traits (8)

Marginal $\tau^{\star}$

Model	Aggr. Strategy (N.annot)
Avg	All tissues, All traits (0), Brain tissues, Brain traits (1), Blood cell types, Blood traits (0)
Max	All tissues, All traits (1), Brain tissues, Brain traits (1), Blood cell types, Blood traits (0)

Joint $\tau^{\star}$

Model	Aggr. Strategy (N.annot)
Avg	All tissues, All traits (0), Brain tissues, Brain traits (0), Blood cell types, Blood traits (0)
Max	All tissues, All traits (1), Brain tissues, Brain traits (1), Blood cell types, Blood traits (0)

PANEL C

Enrichment

Model	Aggr. Strategy (N.annot)
DNase	All tissues, All traits (4), Brain tissues, Brain traits (4), Blood cell types, Blood traits (4)
H3K27ac	All tissues, All traits (4), Brain tissues, Brain traits (4), Blood cell types, Blood traits (4)
H3K4me1	All tissues, All traits (4), Brain tissues, Brain traits (4), Blood cell types, Blood traits (4)
H3K4me3	All tissues, All traits (4), Brain tissues, Brain traits (4), Blood cell types, Blood traits (4)

Marginal $\tau^{\star}$

Model	Aggr. Strategy (N.annot)
DNase	All tissues, All traits (0), Brain tissues, Brain traits (0), Blood cell types, Blood traits (0)
H3K27ac	All tissues, All traits (0), Brain tissues, Brain traits (0), Blood cell types, Blood traits (0)
H3K4me1	All tissues, All traits (0), Brain tissues, Brain traits (0), Blood cell types, Blood traits (0)
H3K4me3	All tissues, All traits (1), Brain tissues, Brain traits (2), Blood cell types, Blood traits (0)

Joint $\tau^{\star}$

Model	Aggr. Strategy (N.annot)
DNase	All tissues, All traits (0), Brain tissues, Brain traits (0), Blood cell types, Blood traits (0)
H3K27ac	All tissues, All traits (0), Brain tissues, Brain traits (0), Blood cell types, Blood traits (0)
H3K4me1	All tissues, All traits (0), Brain tissues, Brain traits (0), Blood cell types, Blood traits (0)
H3K4me3	All tissues, All traits (1), Brain tissues, Brain traits (1), Blood cell types, Blood traits (0)

Supplementary Table 6. S-LDSC results for marginal analysis of non-tissue-specific allelic-effect DeepSEAΔ and BasenjiΔ annotations conditioned on non-tissue-specific variant-level joint model. Standardized Effect sizes ($\tau^\star$) and Enrichment (E) of 8 non-tissue-specific DeepSEAΔ and 8 non-tissue-specific BasenjiΔ annotations, when conditioned on the non-tissue-specific variant-level joint model (baseline-LD model + 7 annotations from Supplementary Figure 6). Results are meta-analyzed across 41 traits.

Annotation	$\tau^\star$	se($\tau^\star$)	p($\tau^\star$)	E	se (E)	p(E)
DeepSEAΔ-DNase-Avg (0.3%)	0.07	0.07	0.32	2.3	0.21	3.3e-07
DeepSEAΔ-DNase-Max (2.0%)	-0.034	0.066	0.61	1.6	0.1	1.5e-05
DeepSEAΔ-H3K27ac-Avg (0.2%)	0.056	0.075	0.45	2	0.12	9.7e-10
DeepSEAΔ-H3K27ac-Max (0.9%)	0.02	0.064	0.75	1.7	0.095	3.3e-10
DeepSEAΔ-H3K4me1-Avg (0.3%)	0.014	0.057	0.81	1.7	0.066	1.9e-12
DeepSEAΔ-H3K4me1-Max (1.7%)	-0.051	0.06	0.4	1.4	0.061	2.7e-07
DeepSEAΔ-H3K4me3-Avg (0.1%)	0.078	0.07	0.27	2.6	0.16	0.00042
DeepSEAΔ-H3K4me3-Max (0.7%)	0.069	0.071	0.33	2	0.087	2.6e-13
BasenjiΔ-DNase-Avg (0.3%)	-0.001	0.13	0.99	2.7	0.36	0.00038
BasenjiΔ-DNase-Max (2.0%)	0.14	0.075	0.071	2.3	0.17	1.1e-10
BasenjiΔ-H3K27ac-Avg (0.3%)	0.19	0.11	0.075	2.4	0.18	6.3e-11
BasenjiΔ-H3K27ac-Max (0.9%)	0.078	0.036	0.032	2.1	0.11	1.3e-18
BasenjiΔ-H3K4me1-Avg (0.3%)	0.14	0.063	0.027	2	0.1	5.6e-16
BasenjiΔ-H3K4me1-Max (1.7%)	0.13	0.05	0.0093	1.9	0.091	4.2e-16
BasenjiΔ-H3K4me3-Avg (0.1%)	0.16	0.071	0.023	3.2	0.19	6.8e-14
BasenjiΔ-H3K4me3-Max (0.7%)	0.32	0.053	7.7e-10	2.6	0.13	3.3e-18

Supplementary Table 7. S-LDSC results for marginal analysis of non-tissue-specific allelic-effect DeepSEAΔ and BasenjiΔ annotations conditioned on baseline-LD model. Standardized Effect sizes ($\tau^{\star}$) and Enrichment (E) of 8 non-tissue-specific DeepSEAΔ and 8 non-tissue-specific BasenjiΔ annotations, when conditioned on the baseline-LD model. Results are meta-analyzed across 41 independent traits.

Annotation	$\tau^{\star}$	se($\tau^{\star}$)	p($\tau^{\star}$)	E	se (E)	p(E)
DeepSEAΔ-DNase-Avg (0.3%)	-0.059	0.08	0.46	2	0.22	2.9e-05
DeepSEAΔ-DNase-Max (2.0%)	-0.24	0.079	0.0019	1.3	0.11	7.7e-05
DeepSEAΔ-H3K27ac-Avg (0.2%)	0.046	0.073	0.52	2	0.12	6e-13
DeepSEAΔ-H3K27ac-Max (0.9%)	-0.05	0.068	0.47	1.7	0.093	2.7e-11
DeepSEAΔ-H3K4me1-Avg (0.3%)	-0.054	0.061	0.38	1.6	0.062	3.6e-14
DeepSEAΔ-H3K4me1-Max (1.7%)	-0.21	0.068	0.002	1.3	0.059	4.2e-06
DeepSEAΔ-H3K4me3-Avg (0.1%)	0.19	0.069	0.006	2.9	0.18	1.7e-11
DeepSEAΔ-H3K4me3-Max (0.7%)	0.074	0.076	0.34	2.1	0.095	2.1e-16
BasenjiΔ-DNase-Avg (0.3%)	-0.037	0.12	0.77	2.6	0.36	3.9e-05
BasenjiΔ-DNase-Max (2.0%)	0.032	0.07	0.65	2.2	0.17	1.3e-06
BasenjiΔ-H3K27ac-Avg (0.2%)	0.21	0.1	0.042	2.4	0.18	7e-08
BasenjiΔ-H3K27ac-Max (0.9%)	0.011	0.036	0.76	2.1	0.11	5.2e-14
BasenjiΔ-H3K4me1-Avg (0.3%)	0.089	0.056	0.11	1.9	0.097	1.6e-
BasenjiΔ-H3K4me1-Max (1.7%)	0.034	0.046	0.45	1.9	0.089	1.6e-12
BasenjiΔ-H3K4me3-Avg (0.1%)	0.22	0.075	0.0028	3.2	0.21	4.6e-13
BasenjiΔ-H3K4me3-Max (0.7%)	0.33	0.058	1.1e-08	2.6	0.14	6.3e-16

Supplementary Table 8. S-LDSC results for marginal analysis of non-tissue-specific variant-level BasenjiV annotations conditioned on the non-tissue-specific variant-level joint model and the non-tissue-specific final joint model. Standardized Effect sizes ($\tau^{\star}$) and Enrichment (E) of 8 non-tissue-specific Basenji variant level annotations, when conditioned on (Top) non-tissue-specific variant-level joint model (baseline-LD model + 7 annotations from Supplementary Figure 6) and (Bottom) non-tissue-specific final joint model comprising of the non-tissue-specific variant-level joint model plus 1 significant allelic-effect annotation (BasenjiΔ-H34me3-Max) from Figure 2. Results are meta-analyzed across 41 independent traits.

Conditional on non-tissue-specific variant-level joint model

Annotation	$\tau^{\star}$	se($\tau^{\star}$)	p($\tau^{\star}$)	E	se (E)	p(E)
BasenjiV-DNase-Avg (1.6%)	-0.085	0.064	0.18	2.5	0.19	1.4e-13
BasenjiV-DNase-Max (13.2%)	-0.052	0.06	0.38	1.8	0.075	2.3e-19
BasenjiV-H3K27ac-Avg (3.2%)	0.047	0.055	0.39	2.5	0.14	1.2e-17
BasenjiV-H3K27ac-Max (13.7%)	-0.018	0.03	0.55	1.8	0.073	1e-17
BasenjiV-H3K4me1-Avg (5.5%)	0.041	0.04	0.31	2	0.075	1.6e-18
BasenjiV-H3K4me1-Max (23.2%)	-0.046	0.025	0.067	1.6	0.046	1.6e-19
BasenjiV-H3K4me3-Avg (1.7%)	0.017	0.058	0.77	3.7	0.24	5.1e-17
BasenjiV-H3K4me3-Max (8.8%)	0.029	0.033	0.38	2	0.083	1.6e-17

Conditional on non-tissue-specific final joint model

Annotation	$\tau^{\star}$	se($\tau^{\star}$)	p($\tau^{\star}$)	E	se (E)	p(E)
BasenjiV-DNase-Avg (1.6%)	-0.11	0.065	0.08	1.8	0.074	1.4e-38
BasenjiV-DNase-Max (13.2%)	-0.11	0.065	0.099	2.5	0.19	8.6e-32
BasenjiV-H3K27ac-Avg (3.2%)	0.0051	0.055	0.93	2.5	0.13	1.3e-33
BasenjiV-H3K27ac-Max (13.7%)	-0.075	0.03	0.012	1.8	0.072	1.5e-31
BasenjiV-H3K4me1-Avg (5.5%)	-0.011	0.04	0.79	1.9	0.073	7.9e-32
BasenjiV-H3K4me1-Max (23.2%)	-0.11	0.036	0.76	1.6	0.046	3.5e-33
BasenjiV-H3K4me3-Avg (1.7%)	-0.031	0.061	0.61	3.5	0.23	5.3e-34
BasenjiV-H3K4me3-Max (8.8%)	-0.026	0.036	0.47	1.9	0.081	3.4e-32

Supplementary Table 9. The 11 conservation related terms in the baseline-LD model. The names of the 11 annotations related to conservation that were removed in the analysis with no conservation in Tables 10, 16 and 22. See Data Availability for the full list of baseline-LD annotations.

Conservation related Annotations
Conserved_LindbladToh[35]
Conserved_LindbladToh.extend.500[35]
Conserved_Vertebrate_phastCons46way[36]
Conserved_Vertebrate_phastCons46way.extend.500[36]
Conserved_Mammal_phastCons46way[36]
Conserved_Mammal_phastCons46way.extend.500[36]
Conserved_Primate_phastCons46way[36]
Conserved_Primate_phastCons46way.extend.500[36]
GERP.NS[37,5]
GERP.RSsup4[37,5]
Backgrd_Selection_Stat[38]

Supplementary Table 10. **S-LDSC results for marginal analysis of non-tissue-specific allelic-effect DeepSEAΔ and BasenjiΔ annotations conditioned on the non-tissue-specific variant-level model without any conservation related annotations**. Standardized Effect sizes ($\tau^{\star}$) and Enrichment (E) of 8 non-tissue-specific DeepSEAΔ and 8 non-tissue-specific BasenjiΔ annotations, when conditioned on the non-tissue-specific variant-level model (baseline-LD model + 7 annotations from Supplementary Figure 6) without any conservation related annotations. Results are meta-analyzed across 41 independent traits.

Annotation	$\tau^{\star}$	se($\tau^{\star}$)	p($\tau^{\star}$)	E	se (E)	p(E)
DeepSEAΔ-DNase-Avg (0.3%)	0.34	0.083	3.4e-05	3	0.19	4.7e-13
DeepSEAΔ-DNase-Max (2.0%)	0.32	0.093	0.00057	2.1	0.12	7.6e-13
DeepSEAΔ-H3K27ac-Avg (0.2%)	0.44	0.088	6.3e-07	2.6	0.12	1.1e-24
DeepSEAΔ-H3K27ac-Max (0.9%)	0.4	0.077	2.4e-07	2.2	0.094	4.6e-22
DeepSEAΔ-H3K4me1-Avg (0.3%)	0.38	0.074	2e-07	2.1	0.067	2.4e-24
DeepSEAΔ-H3K4me1-Max (1.7%)	0.35	0.082	2.1e-05	1.9	0.068	7.1e-18
DeepSEAΔ-H3K4me3-Avg (0.1%)	0.34	0.087	7.3e-05	3.5	0.19	2.7e-18
DeepSEAΔ-H3K4me3-Max (0.7%)	0.3	0.085	0.00043	2.4	0.098	3.7e-19
BasenjiΔ-DNase-Avg (0.3%)	0.21	0.13	0.12	3.1	0.36	1.6e-14
BasenjiΔ-DNase-Max (2.0%)	0.33	0.07	3e-06	2.4	0.16	8.7e-22
BasenjiΔ-H3K27ac-Avg (0.2%)	0.23	0.12	0.049	2.4	0.19	9e-26
BasenjiΔ-H3K27ac-Max (0.9%)	0.11	0.039	0.0071	2	0.11	2.2e-24
BasenjiΔ-H3K4me1-Avg (0.3%)	0.28	0.066	1.5e-05	2	0.1	8.9e-27
BasenjiΔ-H3K4me1-Max (1.7%)	0.22	0.04	7.1e-08	1.9	0.088	2.3e-24
BasenjiΔ-H3K4me3-Avg (0.1%)	0.18	0.076	0.015	3.4	0.21	7.6e-25
BasenjiΔ-H3K4me3-Max (0.7%)	0.3	0.058	1.7e-07	2.6	0.15	2.1e-28

Supplementary Table 11. **Weighted k-mer enrichments for significant allelic-effect Basenji annotations**. We report the weighted k-mer enrichment and enrichment p-value for the top significantly enriched k-mers ($1 \leq k \leq 5$) for the two allelic effect annotations found significant: BasenjiΔ-H3K4me3-Max and BasenjiΔ-H3K4me3-brain-Max annotations. The Bonferonni corrcection threshold is $0.05/(4*682)$ where 682 is the number of k-mers analyzed for the 2 significant variant-level annotations analyzed in Table 50 and the 2 significant allelic-effect annotations analyzed here. We also report results for simple GC-rich motifs (below the horizontal line) for comparison purposes.

BasenjiΔ-H3K4me3-Max		
kmer	enrichment	pvalue
CGGCG	4.1	3.6e-10
CGCGC	4.1	3.5e-10
CGCCG	4.0	7.4e-10
CC	1.14	0.39
CG	1.22	0.33
C	1.07	0.44
BasenjiΔ-H3K4me3-brain-Max		
kmer	enrichment	pvalue
CGCGC	6.2	1.1e-25
CGGCG	6.1	4.9e-25
CGCCG	6	2.8e-24
CGCG	4.3	1.8e-11
CGCGG	4.2	4.9e-11
CCGCG	4.2	9.8e-11
CGCGA	3.7	5.4e-08
AGCGC	3.5	2.8e-07
CCCGC	3.4	8.9e-07
CGCCC	3.3	2.2e-06
ACGCG	3.2	7.7e-06
CC	1.23	0.32
CG	1.45	0.32
C	1.12	0.41

Supplementary Table 12. **Enrichment of two 9-mers of interest for significant allelic-effect BasenjiΔ annotations**. We report the weighted k-mer enrichment and enrichment p-value of two 9-mers, GCGGTGGCT and GTGGTGGCT, with previously reported evidence of being connected to trait architecture[15], with respect to the two allelic effect annotations found significant: BasenjiΔ-H3K4me3-Max and BasenjiΔ-H3K4me3-brain-Max annotations. We additionally also compare against the same annotation for blood (BasenjiΔ-H3K4me3-blood-Max) for a better comparison.

BasenjiΔ-H3K4me3-Max		
kmer	enrichment	pvalue
GCGGTGGCT	1.12	9.7e-08
GTGGTGGCT	0.96	1.00
BasenjiΔ-H3K4me3-brain-Max		
kmer	enrichment	pvalue
GCGGTGGCT	1.03	0.003
GTGGTGGCT	0.85	1.00
BasenjiΔ-H3K4me3-blood-Max		
kmer	enrichment	pvalue
GCGGTGGCT	1.19	3.3e-19
GTGGTGGCT	0.93	1.00

Supplementary Table 13. **AUROC of various sets of annotations incorporating non-tissue-specific allelic-effect deep learning annotations in predicting** $12,296$ **NIH GWAS SNPs**. We report the AUROC for a gradient boosting model trained using each respective set of annotations, either without including baseline-LD model annotations; including baseline-LD model annotations; or including annotations from the non-tissue-specific variant-level joint model (baseline-LD model + 7 annotations; baseline-LD+7).

Feature	GWAS SNPs
DeepSEAΔ-Avg/Max	0.584
BasenjiΔ-Avg/Max	0.592
DeepSEAΔ-All	0.602
BasenjiΔ-All	0.611
baseline-LD	0.758
baseline-LD + DeepSEAΔ-Avg/Max	0.767
baseline-LD + BasenjiΔ-Avg/Max	0.767
baseline-LD + DeepSEAΔ-All	0.770
baseline-LD + BasenjiΔ-All	0.772
baseline-LD+7	0.762
baseline-LD+7 + DeepSEAΔ-Avg/Max	0.766
baseline-LD+7 + BasenjiΔ-Avg/Max	0.769
baseline-LD+7 + DeepSEAΔ-All	0.771
baseline-LD+7 + BasenjiΔ-All	0.773

Supplementary Table 14. **Enrichment of NIH GWAS SNPs for non-tissue-specific allelic-effect annotations**. We report the enrichment in deep learning annotations (along with Jackknife standard error) at top 12, 296 known disease-associated SNPs from NIH GWAS catalog[12].

Feature	GWAS SNPs
BasenjiΔ-DNase-Avg	1.93 (0.020)
BasenjiΔ-DNase-Max	1.70 (0.012)
BasenjiΔ-H3K27ac-Avg	1.76 (0.01)
BasenjiΔ-H3K27ac-Max	1.63 (0.009)
BasenjiΔ-H3K4me1-Avg	1.54 (0.008)
BasenjiΔ-H3K4me1-Max	1.53 (0.008)
BasenjiΔ-H3K4me3-Avg	2.08 (0.022)
BasenjiΔ-H3K4me3-Max	1.79 (0.011)
DeepSEAΔ-DNase-Avg	1.64 (0.01)
DeepSEAΔ-DNase-Max	1.41 (0.007)
DeepSEAΔ-H3K27ac-Avg	1.57 (0.009)
DeepSEAΔ-H3K27ac-Max	1.46 (0.008)
DeepSEAΔ-H3K4me1-Avg	1.43 (0.007)
DeepSEAΔ-H3K4me1-Max	1.33 (0.006)
DeepSEAΔ-H3K4me3-Avg	1.67 (0.013)
DeepSEAΔ-H3K4me3-Max	1.50 (0.007)

Supplementary Table 15. **S-LDSC results for marginal analysis of blood-specific allelic-effect deep learning annotations against blood-specific variant-level joint model and 1 significant non-tissue-specific BasenjiΔ annotation**. Standardized Effect sizes ($\tau^\star$) and Enrichment (E) of 8 blood-specific DeepSEAΔ and 8 blood-specific BasenjiΔ annotations, when conditioned on the blood-specific joint model annotations (baseline-LD model + 7 non-tissue-specific annotations from Supplementary Figure 6 + 6 blood-specific Roadmap and ChromHMM annotations from Supplementary Figure 11) and 1 significant allelic-effect annotation (BasenjiΔ-H3K4me3-Max) from Table 6. Results are meta-analyzed across 11 blood-related traits.

Annotation	$\tau^\star$	se($\tau^\star$)	p($\tau^\star$)	E	se (E)	p(E)
DeepSEAΔ-DNase-blood-Avg (0.2%)	-0.4	0.12	0.00074	2.3	0.38	1.8e-05
DeepSEAΔ-DNase-blood-Max (0.6%)	-0.35	0.11	0.0011	2	0.26	5.1e-11
DeepSEAΔ-H3K27ac-blood-Avg (0.2%)	-0.23	0.11	0.028	2.6	0.23	1.6e-08
DeepSEAΔ-H3K27ac-blood-Max (0.5%)	-0.34	0.11	0.002	2.1	0.16	1.8e-08
DeepSEAΔ-H3K4me1-blood-Avg (0.3%)	-0.35	0.13	0.0035	1.9	0.16	1.3e-08
DeepSEAΔ-H3K4me1-blood-Max (0.8%)	-0.34	0.12	0.0023	1.7	0.14	6.4e-07
DeepSEAΔ-H3K4me3-blood-Avg (0.1%)	-0.3	0.11	0.0063	2.7	0.35	4.3e-08
DeepSEAΔ-H3K4me3-blood-Max (0.4%)	-0.38	0.14	0.0056	2.2	0.24	7.9e-10
BasenjiΔ-DNase-blood-Avg (0.2%)	-0.31	0.2	0.12	5	0.41	9.5e-11
BasenjiΔ-DNase-blood-Max (0.6%)	-0.012	0.22	0.96	4.7	0.28	4e-08
BasenjiΔ-H3K27ac-blood-Avg (0.2%)	0.34	0.17	0.046	4.8	0.29	1.5e-08
BasenjiΔ-H3K27ac-blood-Max (0.5%)	0.23	0.17	0.17	4	0.19	2.9e-08
BasenjiΔ-H3K4me1-blood-Avg (0.3%)	0.033	0.1	0.75	3.6	0.2	4.9e-09
BasenjiΔ-H3K4me1-blood-Max (0.8%)	-0.006	0.15	0.97	3.3	0.17	1.3e-08
BasenjiΔ-H3K4me3-blood-Avg (0.1%)	0.57	0.33	0.083	6.5	0.55	2.6e-09
BasenjiΔ-H3K4me3-blood-Max (0.4%)	0.39	0.3	0.21	4.7	0.3	7.8e-09

Supplementary Table 16. **S-LDSC results for marginal analysis of blood-specific allelic-effect deep learning annotations against blood-specific variant-level joint model and 1 significant non-tissue-specific BasenjiΔ annotation but without any conservation related annotation**. Standardized Effect sizes ($\tau^\star$) and Enrichment (E) of 8 blood-specific DeepSEAΔ and 8 blood-specific BasenjiΔ annotations, when conditioned on the blood-specific variant-level joint model (baseline-LD model + 7 non-tissue-specific annotations from Supplementary Figure 6 + 6 blood-specific Roadmap and ChromHMM annotations from Supplementary Figure 11) and BasenjiΔ-H3K4me3-Max but without the conservation related annotation. Results are meta-analyzed across 11 blood-related traits.

Annotation	$\tau^\star$	se($\tau^\star$)	p($\tau^\star$)	E	se (E)	p(E)
DeepSEAΔ-DNase-blood-Avg (0.2%)	-0.17	0.13	0.18	2.6	0.38	0.0089
DeepSEAΔ-DNase-blood-Max (0.6%)	-0.067	0.1	0.51	2.3	0.26	0.0035
DeepSEAΔ-H3K27ac-blood-Avg (0.2%)	0.078	0.1	0.45	3.1	0.23	1.9e-05
DeepSEAΔ-H3K27ac-blood-Max (0.5%)	-0.035	0.1	0.74	2.4	0.16	0.00016
DeepSEAΔ-H3K4me1-blood-Avg (0.3%)	-0.026	0.087	0.76	2.2	0.16	0.00011
DeepSEAΔ-H3K4me1-blood-Max (0.8%)	-0.007	0.085	0.93	2	0.14	0.00054
DeepSEAΔ-H3K4me3-blood-Avg (0.1%)	-0.031	0.1	0.77	3.3	0.35	0.00039
DeepSEAΔ-H3K4me3-blood-Max (0.4%)	-0.11	0.14	0.43	2.6	0.23	0.00079
BasenjiΔ-DNase-blood-Avg (0.2%)	-0.042	0.17	0.8	5	0.41	2.2e-05
BasenjiΔ-DNase-blood-Max (0.6%)	0.13	0.18	0.47	4.6	0.29	6.2e-07
BasenjiΔ-H3K27ac-blood-Avg (0.2%)	0.42	0.12	0.00039	4.8	0.29	5.1e-09
BasenjiΔ-H3K27ac-blood-Max (0.5%)	0.32	0.11	0.0047	4	0.2	3.5e-09
BasenjiΔ-H3K4me1-blood-Avg (0.3%)	0.19	0.094	0.044	3.5	0.2	9.8e-09
BasenjiΔ-H3K4me1-blood-Max (0.8%)	0.14	0.11	0.17	3.2	0.17	3.8e-08
BasenjiΔ-H3K4me3-blood-Avg (0.1%)	0.6	0.2	0.0021	6.5	0.49	1.9e-07
BasenjiΔ-H3K4me3-blood-Max (0.4%)	0.4	0.14	0.0049	4.7	0.3	4.9e-09

Supplementary Table 17. **S-LDSC results for marginal analysis of blood-specific allelic-effect deep learning annotations against blood-specific variant-level joint model**. Standardized Effect sizes ($\tau^\star$) and Enrichment (E) of 8 blood-specific DeepSEAΔ and 8 blood-specific BasenjiΔ annotations, when conditioned on the blood-specific variant-level joint model annotations (baseline-LD model + 7 non-tissue-specific annotations from Supplementary Figure 6 + 6 blood-specific Roadmap and ChromHMM annotations from Supplementary Figure 11). Results are meta-analyzed across 11 blood-related traits.

Annotation	$\tau^\star$	se($\tau^\star$)	p($\tau^\star$)	E	se (E)	p(E)
DeepSEAΔ-DNase-blood-Avg (0.2%)	-0.24	0.14	0.089	2.4	0.38	0.00027
DeepSEAΔ-DNase-blood-Max (0.6%)	-0.18	0.1	0.075	2	0.26	5e-06
DeepSEAΔ-H3K27ac-blood-Avg (0.2%)	-0.073	0.1	0.48	2.7	0.23	9.2e-09
DeepSEAΔ-H3K27ac-blood-Max (0.5%)	-0.22	0.12	0.066	2.1	0.16	1.1e-07
DeepSEAΔ-H3K4me1-blood-Avg (0.3%)	-0.21	0.089	0.021	1.9	0.16	2.6e-08
DeepSEAΔ-H3K4me1-blood-Max (0.8%)	-0.22	0.1	0.036	1.7	0.14	7.7e-06
DeepSEAΔ-H3K4me3-blood-Avg (0.1%)	-0.12	0.11	0.27	2.9	0.36	1.7e-06
DeepSEAΔ-H3K4me3-blood-Max (0.4%)	-0.23	0.15	0.11	2.3	0.25	1.8e-07
BasenjiΔ-DNase-blood-Avg (0.2%)	0.039	0.17	0.81	5.1	0.41	3.7e-11
BasenjiΔ-DNase-blood-Max (0.6%)	0.22	0.18	0.22	4.7	0.29	7.1e-08
BasenjiΔ-H3K27ac-blood-Avg (0.2%)	0.45	0.12	0.00025	4.8	0.31	2.3e-07
BasenjiΔ-H3K27ac-blood-Max (0.5%)	0.36	0.11	0.0017	4	0.21	2.1e-07
BasenjiΔ-H3K4me1-blood-Avg (0.3%)	0.22	0.096	0.021	3.6	0.21	4.5e-08
BasenjiΔ-H3K4me1-blood-Max (0.8%)	0.18	0.11	0.11	3.3	0.18	1.1e-07
BasenjiΔ-H3K4me3-blood-Avg (0.1%)	0.67	0.2	0.00067	6.5	0.52	5.6e-09
BasenjiΔ-H3K4me3-blood-Max (0.4%)	0.47	0.15	0.0018	4.8	0.32	1.2e-07

Supplementary Table 18. **S-LDSC results for marginal analysis of blood-specific allelic-effect deep learning annotations conditioned on the non-tissue-specific final joint model**. Standardized Effect sizes ($\tau^{\star}$) and Enrichment (E) of 8 blood-specific DeepSEAΔ and 8 blood-specific BasenjiΔ annotations, when conditioned on the non-tissue-specific final joint model. Results are meta-analyzed across 11 blood-related traits.

Annotation	$\tau^{\star}$	se($\tau^{\star}$)	p($\tau^{\star}$)	E	se (E)	p(E)
DeepSEAΔ-DNase-blood-Avg (0.2%)	-0.37	0.14	0.0064	2.9	0.52	1.8e-06
DeepSEAΔ-DNase-blood-Max (0.6%)	-0.49	0.12	2.4e-05	2	0.28	1.6e-06
DeepSEAΔ-H3K27ac-blood-Avg (0.2%)	0.024	0.11	0.83	3.3	0.29	1.2e-18
DeepSEAΔ-H3K27ac-blood-Max (0.5%)	-0.23	0.11	0.038	2.4	0.2	1.2e-16
DeepSEAΔ-H3K4me1-blood-Avg (0.3%)	-0.33	0.099	0.0011	2	0.18	2e-10
DeepSEAΔ-H3K4me1-blood-Max (0.8%)	-0.47	0.098	1.4e-06	1.7	0.15	4.8e-08
DeepSEAΔ-H3K4me3-blood-Avg (0.1%)	-0.071	0.12	0.56	3.8	0.41	1.4e-08
DeepSEAΔ-H3K4me3-blood-Max (0.4%)	-0.23	0.13	0.078	2.7	0.28	5.7e-09
BasenjiΔ-DNase-blood-Avg (0.2%)	0.56	0.2	0.0049	7.2	0.57	1.6e-09
BasenjiΔ-DNase-blood-Max (0.6%)	0.69	0.21	0.00081	5.8	0.37	5.1e-09
BasenjiΔ-H3K27ac-blood-Avg (0.2%)	1.8	0.26	1.5e-12	6.2	0.38	6.6e-09
BasenjiΔ-H3K27ac-blood-Max (0.5%)	1.5	0.22	1.1e-11	4.8	0.25	1.5e-08
BasenjiΔ-H3K4me1-blood-Avg (0.3%)	1.2	0.2	7.7e-09	4.6	0.28	2.1e-09
BasenjiΔ-H3K4me1-blood-Max (0.8%)	1	0.19	8.7e-08	4	0.22	3.4e-09
BasenjiΔ-H3K4me3-blood-Avg (0.1%)	1.9	0.36	1.3e-07	8.6	0.68	3e-09
BasenjiΔ-H3K4me3-blood-Max (0.4%)	2.1	0.36	7.4e-09	5.7	0.34	1.3e-08

Supplementary Table 19. **AUROC of various sets of annotations incorporating blood-specific allelic-effect deep learning annotations in predicting 8,741 fine-mapped autoimmune disease SNPs**. We report the AUROC for a gradient boosting model trained using each respective set of annotations, either without including baseline-LD model annotations; including baseline-LD model annotations; or including annotations from the blood-specific variant-level joint model plus BasenjiΔ-H3K4me3-Max (baseline-LD+7+6blood+1).

Feature	Farh et al
DeepSEAΔ-blood-Avg/Max	0.613
BasenjiΔ-blood-Avg/Max	0.672
DeepSEAΔ-blood-All	0.633
BasenjiΔ-blood-All	0.684
baseline-LD	0.841
baseline-LD + DeepSEAΔ-blood-Avg/Max	0.847
baseline-LD + BasenjiΔ-blood-Avg/Max	0.852
baseline-LD + DeepSEAΔ-blood-All	0.848
baseline-LD + BasenjiΔ-blood-All	0.851
baseline-LD+7+6blood+1	0.848
baseline-LD+7+6blood+1 + DeepSEAΔ-blood-Avg/Max	0.847
baseline-LD+7+6blood+1 + BasenjiΔ-blood-Avg/Max	0.851
baseline-LD+7+6blood+1 + DeepSEAΔ-blood-All	0.849
baseline-LD+7+6blood+1 + BasenjiΔ-blood-All	0.851

Supplementary Table 20. **Enrichment of fine-mapped autoimmune disease SNPs for blood-specific allelic-effect annotations**. We report the enrichment in blood-specific deep learning allelic-effect annotations (along with Jackknife standard error) for 8741 fine-mapped SNPs in immune-related traits[17].

Feature	Farh et al
BasenjiΔ-DNase-blood-Avg	2.59 (0.05)
BasenjiΔ-DNase-blood-Max	2.41 (0.04)
BasenjiΔ-H3K27ac-blood-Avg	2.44 (0.03)
BasenjiΔ-H3K27ac-blood-Max	2.29 (0.03)
BasenjiΔ-H3K4me1-blood-Avg	2.21 (0.03)
BasenjiΔ-H3K4me1-blood-Max	2.09 (0.02)
BasenjiΔ-H3K4me3-blood-Avg	2.52 (0.03)
BasenjiΔ-H3K4me3-blood-Max	2.40 (0.03)
DeepSEAΔ-DNase-blood-Avg	1.87 (0.03)
DeepSEAΔ-DNase-blood-Max	1.62 (0.02)
DeepSEAΔ-H3K27ac-blood-Avg	1.83 (0.03)
DeepSEAΔ-H3K27ac-blood-Max	1.60 (0.01)
DeepSEAΔ-H3K4me1-blood-Avg	1.58 (0.01)
DeepSEAΔ-H3K4me1-blood-Max	1.45 (0.01)
DeepSEAΔ-H3K4me3-blood-Avg	2.01 (0.04)
DeepSEAΔ-H3K4me3-blood-Max	1.66 (0.02)

Supplementary Table 21. S-LDSC results for marginal analysis of brain-specific allelic-effect deep learning annotations against brain-specific variant-level joint model and 1 significant non-tissue-specific BasenjiΔ annotation. Standardized Effect sizes ($\tau^\star$) and Enrichment (E) of 8 brain-specific DeepSEAΔ and 8 brain-specific BasenjiΔ annotations, when conditioned on brain-specific variant-level joint model (baseline-LD model + 7 non-tissue-specific annotations from Supplementary Figure 6 + DeepSEAV-H3K4me3-brain-Max and BasenjiV-H3K27ac-brain-Max (the 2 significant brain-specific variant-level annotations; Supplementary Figure 12) + 4 additional brain-specific annotations from Supplementary Figure 15) and 1 significant non-tissue-specific annotation from Table 6). Results are meta-analyzed across 8 brain-related traits.

Annotation	$\tau^\star$	se($\tau^\star$)	p($\tau^\star$)	E	se (E)	p(E)
DeepSEAΔ-DNase-brain-Avg (0.4%)	-0.027	0.075	0.72	1.9	0.25	6.1e-05
DeepSEAΔ-DNase-brain-Max (0.7%)	-0.011	0.074	0.88	1.8	0.26	2.8e-05
DeepSEAΔ-H3K27ac-brain-Avg (0.2%)	0.029	0.071	0.68	1.8	0.1	6e-07
DeepSEAΔ-H3K27ac-brain-Max (0.4%)	-0.021	0.072	0.77	1.7	0.11	5.6e-07
DeepSEAΔ-H3K4me1-brain-Avg (0.3%)	0.0093	0.065	0.89	1.7	0.086	3.4e-07
DeepSEAΔ-H3K4me1-brain-Max (0.6%)	-0.013	0.069	0.85	1.6	0.1	1.6e-07
DeepSEAΔ-H3K4me3-brain-Avg (0.1%)	-0.001	0.08	0.99	2.5	0.22	3.6e-05
DeepSEAΔ-H3K4me3-brain-Max (0.2%)	-0.055	0.088	0.54	2.5	0.19	6.5e-06
BasenjiΔ-DNase-brain-Avg (0.4%)	0.25	0.083	0.0024	2.9	0.16	1.5e-07
BasenjiΔ-DNase-brain-Max (0.7%)	0.12	0.077	0.12	2.6	0.18	5.9e-06
BasenjiΔ-H3K27ac-brain-Avg (0.2%)	0.11	0.05	0.023	2.1	0.12	4.4e-07
BasenjiΔ-H3K27ac-brain-Max (0.4%)	0.1	0.055	0.071	2	0.15	2.2e-07
BasenjiΔ-H3K4me1-brain-Avg (0.3%)	0.11	0.058	0.058	1.9	0.077	1e-06
BasenjiΔ-H3K4me1-brain-Max (0.6%)	0.083	0.06	0.17	1.9	0.085	1.1e-07
BasenjiΔ-H3K4me3-brain-Avg (0.1%)	0.34	0.097	0.00047	3.5	0.18	2.5e-08
BasenjiΔ-H3K4me3-brain-Max (0.2%)	0.46	0.095	1.4e-06	3.4	0.19	3.2e-08

Supplementary Table 22. S-LDSC results for marginal analysis of brain-specific allelic-effect deep learning annotations against brain-specific variant-level joint model and 1 significant non-tissue-specific BasenjiΔ annotation but without any conservation related annotations. Standardized Effect sizes ($\tau^\star$) and Enrichment (E) of 8 brain-specific DeepSEAΔ and 8 brain-specific BasenjiΔ annotations, when conditioned on the brain-specific variant-level joint model (baseline-LD model + 7 non-tissue-specific annotations from Supplementary Figure 6 + DeepSEAV-H3K4me3-brain-Max and BasenjiV-H3K27ac-brain-Max (the 2 significant brain-specific variant-level annotations; Supplementary Figure 12) + 4 additional brain-specific annotations from Supplementary Figure 15) and BasenjiΔ-H3K4me3-Max but without the conservation related annotations. Results are meta-analyzed across 8 brain-related traits.

Annotation	$\tau^\star$	se($\tau^\star$)	p($\tau^\star$)	E	se (E)	p(E)
DeepSEAΔ-DNase-brain-Avg (0.4%)	0.48	0.078	4.5e-10	2.9	0.19	9.1e-11
DeepSEAΔ-DNase-brain-Max (0.7%)	0.42	0.077	4e-08	2.7	0.2	1.5e-09
DeepSEAΔ-H3K27ac-brain-Avg (0.2%)	0.48	0.074	6.3e-11	2.4	0.1	1.4e-10
DeepSEAΔ-H3K27ac-brain-Max (0.4%)	0.39	0.075	2.5e-07	2.3	0.11	1.1e-09
DeepSEAΔ-H3K4me1-brain-Avg (0.3%)	0.42	0.066	1.8e-10	2.2	0.088	2.4e-11
DeepSEAΔ-H3K4me1-brain-Max (0.6%)	0.43	0.071	1.2e-09	2.2	0.11	5.2e-10
DeepSEAΔ-H3K4me3-brain-Avg (0.1%)	0.44	0.083	9.1e-08	3.8	0.23	1.1e-10
DeepSEAΔ-H3K4me3-brain-Max (0.2%)	0.42	0.092	4.4e-06	3.6	0.2	2.4e-11
BasenjiΔ-DNase-brain-Avg (0.4%)	0.73	0.095	1.5e-14	3.6	0.17	1.7e-13
BasenjiΔ-DNase-brain-Max (0.7%)	0.53	0.079	1.7e-11	3.2	0.18	3.9e-12
BasenjiΔ-H3K27ac-brain-Avg (0.2%)	0.14	0.051	0.0068	2	0.12	1.7e-08
BasenjiΔ-H3K27ac-brain-Max (0.4%)	0.14	0.051	0.0059	2	0.16	2e-07
BasenjiΔ-H3K4me1-brain-Avg (0.3%)	0.3	0.058	1.9e-07	2	0.077	7.9e-10
BasenjiΔ-H3K4me1-brain-Max (0.6%)	0.34	0.06	1.2e-08	2.1	0.086	5.9e-11
BasenjiΔ-H3K4me3-brain-Avg (0.1%)	0.4	0.085	3e-06	4	0.19	1e-10
BasenjiΔ-H3K4me3-brain-Max (0.2%)	0.5	0.079	2.4e-10	3.8	0.19	2.9e-11

Supplementary Table 23. S-LDSC results for marginal analysis of brain-specific allelic-effect deep learning annotations conditioned on the brain-specific variant-level joint model. Standardized Effect sizes ($\tau^\star$) and Enrichment (E) of 8 brain-specific DeepSEAΔ and 8 brain-specific BasenjiΔ annotations, when conditioned on the brain-specific variant-level joint model (baseline-LD model + 7 non-tissue-specific annotations from Supplementary Figure 6 + DeepSEAV-H3K4me3-brain-Max and BasenjiV-H3K27ac-brain-Max (the 2 significant brain-specific variant-level annotations; Supplementary Figure 12) + 4 additional brain-specific annotations from Supplementary Figure 15). Results are meta-analyzed across 8 brain-related traits.

Annotation	$\tau^\star$	se($\tau^\star$)	p($\tau^\star$)	E	se (E)	p(E)
DeepSEAΔ-DNase-brain-Avg (0.4%)	0.0038	0.076	0.96	1.9	0.25	1.7e-06
DeepSEAΔ-DNase-brain-Max (0.7%)	0.022	0.075	0.77	1.8	0.26	1.9e-06
DeepSEAΔ-H3K27ac-brain-Avg (0.2%)	0.065	0.072	0.36	1.8	0.1	1.8e-09
DeepSEAΔ-H3K27ac-brain-Max (0.4%)	0.022	0.073	0.76	1.7	0.11	1.2e-09
DeepSEAΔ-H3K4me1-brain-Avg (0.3%)	0.039	0.065	0.55	1.7	0.087	1.5e-09
DeepSEAΔ-H3K4me1-brain-Max (0.6%)	0.022	0.069	0.76	1.6	0.1	7.6e-11
DeepSEAΔ-H3K4me3-brain-Avg (0.1%)	0.025	0.08	0.75	2.6	0.22	8.5e-07
DeepSEAΔ-H3K4me3-brain-Max (0.2%)	-0.021	0.089	0.81	2.5	0.19	1.3e-08
BasenjiΔ-DNase-brain-Avg (0.4%)	0.29	0.081	0.00041	2.8	0.16	1e-08
BasenjiΔ-DNase-brain-Max (0.7%)	0.16	0.076	0.035	2.5	0.18	3.7e-07
BasenjiΔ-H3K27ac-brain-Avg (0.2%)	0.15	0.048	0.0026	2	0.12	1.1e-08
BasenjiΔ-H3K27ac-brain-Max (0.4%)	0.13	0.048	0.0054	2	0.16	2e-0
BasenjiΔ-H3K4me1-brain-Avg (0.3%)	0.14	0.056	0.014	1.9	0.076	3.8e-08
BasenjiΔ-H3K4me1-brain-Max (0.6%)	0.12	0.058	0.041	1.9	0.083	2.6e-09
BasenjiΔ-H3K4me3-brain-Avg (0.1%)	0.36	0.08	7.7e-06	3.6	0.18	5.5e-10
BasenjiΔ-H3K4me3-brain-Max (0.2%)	0.41	0.074	4.1e-08	3.4	0.19	6.3e-10

Supplementary Table 24. **S-LDSC results for marginal analysis of brain-specific allelic-effect deep learning annotations conditioned on the non-tissue-specific final joint model**. Standardized Effect sizes ($\tau^\star$) and Enrichment (E) of 8 brain-specific DeepSEAΔ and 8 brain-specific BasenjiΔ annotations, when conditioned on the non-tissue-specific final joint model. Results are meta-analyzed across 8 brain-related traits.

Annotation	$\tau^\star$	se($\tau^\star$)	p($\tau^\star$)	E	se (E)	p(E)
DeepSEAΔ-DNase-brain-Avg (0.4%)	0.25	0.073	0.00053	2.4	0.17	1.4e-05
DeepSEAΔ-DNase-brain-Max (0.7%)	0.21	0.072	0.0034	2.2	0.19	3.9e-08
DeepSEAΔ-H3K27ac-brain-Avg (0.2%)	0.3	0.061	9.4e-07	2	0.094	4.8e-09
DeepSEAΔ-H3K27ac-brain-Max (0.4%)	0.28	0.064	1.1e-05	2	0.1	5.2e-07
DeepSEAΔ-H3K4me1-brain-Avg (0.3%)	0.27	0.056	1e-06	1.9	0.08	7e-10
DeepSEAΔ-H3K4me1-brain-Max (0.6%)	0.27	0.062	2e-05	1.9	0.097	1.7e-08
DeepSEAΔ-H3K4me3-brain-Avg (0.1%)	0.44	0.071	4.5e-10	3.4	0.22	7.8e-05
DeepSEAΔ-H3K4me3-brain-Max (0.2%)	0.5	0.072	2.6e-12	3.3	0.19	3.3e-06
BasenjiΔ-DNase-brain-Avg (0.4%)	0.56	0.097	9.1e-09	3.1	0.16	3.5e-09
BasenjiΔ-DNase-brain-Max (0.7%)	0.46	0.073	3.5e-10	2.9	0.16	9.2e-08
BasenjiΔ-H3K27ac-brain-Avg (0.2%)	0.32	0.046	5.4e-12	2.1	0.096	7.8e-08
BasenjiΔ-H3K27ac-brain-Max (0.4%)	0.25	0.048	1.6e-07	2.1	0.13	4.5e-08
BasenjiΔ-H3K4me1-brain-Avg (0.3%)	0.34	0.069	6.3e-07	2.1	0.072	4.8e-09
BasenjiΔ-H3K4me1-brain-Max (0.6%)	0.23	0.084	0.0072	2	0.08	7.6e-11
BasenjiΔ-H3K4me3-brain-Avg (0.1%)	0.79	0.11	2.6e-13	3.7	0.18	1.1e-08
BasenjiΔ-H3K4me3-brain-Max (0.2%)	0.91	0.079	4e-30	3.4	0.18	2.3e-08

Supplementary Table 25. **S-LDSC results for marginal analysis of brain-specific Basenji variant-level annotations and allelic-effect annotations conditioned on either the brain-specific variant-level joint model and BasenjiΔ-H3K4me3-Max or the brain-specific final joint model**. We report the standardized effect sizes (τ^*) and enrichment (E) of Basenji-H3K4me3-brain-Avg, Basenji-H3K4me3-brain-Max and BasenjiΔ-H3K4me3-brain-Avg, when conditioned on either the brain-specific variant-level joint model (baseline-LD model + 7 non-tissue-specific annotations from Supplementary Figure 6 + DeepSEAV-H3K4me3-brain-Max and BasenjiV-H3K27ac-brain-Max (the 2 significant brain-specific variant-level annotations; Supplementary Figure 12) + 4 additional brain-specific annotations from Supplementary Figure 15) and BasenjiΔ-H3K4me3-Max (top panel) or the brain-specific final joint model (bottom panel). Results are meta-analyzed across 8 brain-related traits.

Conditional on brain specific variant-level joint model and BasenjiΔ-H3K4me3-Max

Annotation	$\tau^\star$	se($\tau^\star$)	p($\tau^\star$)	E	se (E)	p(E)
Basenji-H3K4me3-brain-Avg (1.9%)	-0.21	0.097	0.02	3.9	0.29	4.3e-08
Basenji-H3K4me3-brain-Max (3.4%)	0.16	0.11	0.16	3.6	0.19	2.4e-08
BasenjiΔ-H3K4me3-brain-Avg (0.1%)	0.34	0.097	0.00047	3.5	0.18	2.5e-08
BasenjiΔ-H3K4me3-brain-Max (0.2%)	0.46	0.095	1.4e-06	3.4	0.19	3.2e-08

Conditional on brain-specific final joint model

Annotation	$\tau^\star$	se($\tau^\star$)	p($\tau^\star$)	E	se (E)	p(E)
Basenji-H3K4me3-brain-Avg (1.9%)	-0.21	0.098	0.02	3.6	0.3	3e-10
Basenji-H3K4me3-brain-Max (3.4%)	0.049	0.11	0.65	3.4	0.2	1.6e-10
BasenjiΔ-H3K4me3-brain-Avg (0.1%)	-0.62	0.33	0.061	3.3	0.18	1.3e-08
BasenjiΔ-H3K4me3-brain-Max (0.2%)	0.45	0.13	5e-04	3.3	0.18	2.7e-08

Supplementary Table 26. S-LDSC results for marginal analysis of brain-specific allelic-effect deep learning annotations conditioned on the joint model of brain-specific Roadmap and ChromHMM annotations but not the variant-level deep learning annotations. Standardized Effect sizes ($\tau^{\star}$) and Enrichment (E) of 8 brain-specific DeepSEAΔ and 8 brain-specific BasenjiΔ annotations, when conditioned on the joint model in Table 47) but without the variant-level deep learning terms. Results are meta-analyzed across 8 brain-related traits.

Annotation	$\tau^{\star}$	se($\tau^{\star}$)	p($\tau^{\star}$)	E	se (E)	p(E)
DeepSEAΔ-DNase-brain-Avg (0.4%)	0.22	0.074	0.0024	2.3	0.2	1.8e-05
DeepSEAΔ-DNase-brain-Max (0.7%)	0.21	0.073	0.0032	2.2	0.22	0.00014
DeepSEAΔ-H3K27ac-brain-Avg (0.2%)	0.26	0.066	8.5e-05	2	0.1	1.5e-07
DeepSEAΔ-H3K27ac-brain-Max (0.4%)	0.24	0.067	0.0003	2	0.11	1.7e-06
DeepSEAΔ-H3K4me1-brain-Avg (0.3%)	0.21	0.061	0.00068	1.8	0.085	2.6e-08
DeepSEAΔ-H3K4me1-brain-Max (0.6%)	0.21	0.065	0.0011	1.8	0.1	3.3e-06
DeepSEAΔ-H3K4me3-brain-Avg (0.1%)	0.28	0.07	4.6e-05	3.1	0.22	4.8e-08
DeepSEAΔ-H3K4me3-brain-Max (0.2%)	0.31	0.072	1.4e-05	3	0.18	9.9e-09
BasenjiΔ-DNase-brain-Avg (0.4%)	0.43	0.077	2.9e-08	3	0.16	1.5e-10
BasenjiΔ-DNase-brain-Max (0.7%)	0.32	0.074	1.8e-05	2.7	0.17	3.6e-08
BasenjiΔ-H3K27ac-brain-Avg (0.2%)	0.17	0.048	0.00031	2	0.12	1.2e-08
BasenjiΔ-H3K27ac-brain-Max (0.4%)	0.14	0.048	0.0031	2	0.16	1.4e-07
BasenjiΔ-H3K4me1-brain-Avg (0.3%)	0.23	0.056	3.6e-05	1.9	0.076	7.1e-09
BasenjiΔ-H3K4me1-brain-Max (0.6%)	0.17	0.058	0.0038	1.9	0.083	1.4e-09
BasenjiΔ-H3K4me3-brain-Avg (0.1%)	0.44	0.077	1.3e-08	3.5	0.19	4.7e-10
BasenjiΔ-H3K4me3-brain-Max (0.2%)	0.48	0.072	4.6e-11	3.3	0.2	1.8e-10

Supplementary Table 27. **S-LDSC results for brain-specific final joint model**. The results are conditional on the brain-specific variant-level joint model and 1 significant non-tissue-specific allelic-effect annotation. Results are meta-analyzed across 8 brain-related traits

Annotation	$\tau^{\star}$	se($\tau^{\star}$)	p($\tau^{\star}$)	E	se (E)	p(E)
BasenjiΔ-H3K4me3-brain-Avg (0.1%)	-0.62	0.33	0.06	3.3	0.18	1.3e-08
BasenjiΔ-H3K4me3-brain-Max (0.2%)	0.45	0.13	5e-04	3.3	0.18	2.7e-08

Supplementary Table 28. **Number of significantly disease informative non-tissue-specific variant-level DeepSEAV and BasenjiV annotations**. Number of significantly disease informative non-tissue-specific variant-level annotations across different aggregation strategies (A) different deep learning models (DeepSEA and Basenji), (B) different aggregation strategies (Average and Maximum) and (C) different types of epigenomic marks, in terms of marginal enrichment in heritability, conditional $\tau^{\star}$ and joint $\tau^{\star}$ for all traits, blood traits and brain traits respectively. See Supplementary Figure 17 for visual illustration. N.annot in brackets represents the number of significant annotations.

PANEL A

Enrichment

Model	Aggr. Strategy (N.annot)
DeepSEAV	All tissues, All traits (8), Brain tissues, Brain traits (8), Blood cell types, Blood traits (8)
BasenjiV	All tissues, All traits (8), Brain tissues, Brain traits (8), Blood cell types, Blood traits (8)

Marginal $\tau^{\star}$

Model	Aggr. Strategy (N.annot)
DeepSEAV	All tissues, All traits (1), Brain tissues, Brain traits (8), Blood cell types, Blood traits (0)
BasenjiV	All tissues, All traits (0), Brain tissues, Brain traits (8), Blood cell types, Blood traits (4)

Joint $\tau^{\star}$

Model	Aggr. Strategy (N.annot)
DeepSEAV	All tissues, All traits (0), Brain tissues, Brain traits (1), Blood cell types, Blood traits (0)
BasenjiV	All tissues, All traits (0), Brain tissues, Brain traits (1), Blood cell types, Blood traits (0)

PANEL B

Enrichment

Model	Aggr. Strategy (N.annot)
Avg	All tissues, All traits (8), Brain tissues, Brain traits (8), Blood cell types, Blood traits (8)
Max	All tissues, All traits (8), Brain tissues, Brain traits (8), Blood cell types, Blood traits (8)

Marginal $\tau^{\star}$

Model	Aggr. Strategy (N.annot)
Avg	All tissues, All traits (0), Brain tissues, Brain traits (8), Blood cell types, Blood traits (2)
Max	All tissues, All traits (1), Brain tissues, Brain traits (8), Blood cell types, Blood traits (2)

Joint $\tau^{\star}$

Model	Aggr. Strategy (N.annot)
Avg	All tissues, All traits (0), Brain tissues, Brain traits (0), Blood cell types, Blood traits (0)
Max	All tissues, All traits (0), Brain tissues, Brain traits (2), Blood cell types, Blood traits (0)

PANEL C

Enrichment

Model	Aggr. Strategy (N.annot)
DNase	All tissues, All traits (4), Brain tissues, Brain traits (4), Blood cell types, Blood traits (4)
H3K27ac	All tissues, All traits (4), Brain tissues, Brain traits (4), Blood cell types, Blood traits (4)
H3K4me1	All tissues, All traits (4), Brain tissues, Brain traits (4), Blood cell types, Blood traits (4)
H3K4me3	All tissues, All traits (4), Brain tissues, Brain traits (4), Blood cell types, Blood traits (4)

Marginal $\tau^{\star}$

Model	Aggr. Strategy (N.annot)
DNase	All tissues, All traits (0), Brain tissues, Brain traits (4), Blood cell types, Blood traits (0)
H3K27ac	All tissues, All traits (0), Brain tissues, Brain traits (4), Blood cell types, Blood traits (1)
H3K4me1	All tissues, All traits (0), Brain tissues, Brain traits (4), Blood cell types, Blood traits (2)
H3K4me3	All tissues, All traits (1), Brain tissues, Brain traits (4), Blood cell types, Blood traits (1)

Joint $\tau^{\star}$

Model	Aggr. Strategy (N.annot)
DNase	All tissues, All traits (0), Brain tissues, Brain traits (0), Blood cell types, Blood traits (0)
H3K27ac	All tissues, All traits (0), Brain tissues, Brain traits (1), Blood cell types, Blood traits (0)
H3K4me1	All tissues, All traits (0), Brain tissues, Brain traits (0), Blood cell types, Blood traits (0)
H3K4me3	All tissues, All traits (0), Brain tissues, Brain traits (1), Blood cell types, Blood traits (0)

Supplementary Table 29. S-LDSC results for marginal analysis of non-tissue-specific variant-level DeepSEAV and BasenjiV annotations. Standardized Effect sizes ($\tau^{\star}$) and Enrichment (E) of 8 non-tissue-specific variant-level DeepSEAV and 8 non-tissue-specific variant-level BasenjiV annotations, when conditioned on 86 baseline-LD annotations. Results are meta-analyzed across 41 traits.

Annotation	$\tau^{\star}$	se($\tau^{\star}$)	p($\tau^{\star}$)	E	se (E)	p(E)
BasenjiV-DNase-Avg (1.7%)	-0.078	0.07	0.26	2.4	0.2	4.9e-09
BasenjiV-DNase-Max (13.6%)	-0.095	0.065	0.14	1.8	0.079	1.7e-16
BasenjiV-H3K27ac-Avg (3.4%)	0.14	0.059	0.016	2.5	0.14	2.7e-16
BasenjiV-H3K27ac-Max (14.2%)	-0.026	0.031	0.41	1.8	0.074	9.4e-16
BasenjiV-H3K4me1-Avg (5.6%)	0.08	0.042	0.055	2	0.076	2.7e-17
BasenjiV-H3K4me1-Max (23.7%)	-0.057	0.027	0.032	1.6	0.046	1.4e-17
BasenjiV-H3K4me3-Avg (2.0%)	0.17	0.07	0.014	3.7	0.26	1.4e-16
BasenjiV-H3K4me3-Max (9.4%)	0.079	0.036	0.029	2	0.088	5.3e-15
DeepSEAV-DNase-Avg (1.7%)	-0.12	0.11	0.27	2.3	0.2	3e-07
DeepSEAV-DNase-Max (13.6%)	-0.17	0.078	0.028	1.8	0.075	1.1e-15
DeepSEAV-H3K27ac-Avg (3.4%)	0.19	0.071	0.0089	2.4	0.12	1.1e-17
DeepSEAV-H3K27ac-Max (14.2%)	0.0092	0.06	0.88	1.8	0.066	2.6e-17
DeepSEAV-H3K4me1-Avg (5.6%)	0.015	0.059	0.8	1.9	0.059	2.2e-17
DeepSEAV-H3K4me1-Max (23.7%)	-0.13	0.06	0.027	1.6	0.041	8.8e-18
DeepSEAV-H3K4me3-Avg (2.0%)	0.27	0.093	0.0033	4	0.25	7.1e-19
DeepSEAV-H3K4me3-Max (9.4%)	0.25	0.06	4.5e-05	2.2	0.087	2.2e-17

Supplementary Table 30. **S-LDSC results for marginal analysis of non-tissue-specific BiClassCNN annotations**. Standardized Effect sizes ($\tau^{\star}$) and Enrichment (E) of 12 non-tissue-specific BiClassCNN annotations. Results are meta-analyzed across 41 traits.

Annotation	$\tau^{\star}$	se($\tau^{\star}$)	p($\tau^{\star}$)	E	se (E)	p(E)
BiClassCNN-Coding (1.3%)	0.93	0.16	2.5e-09	4.9	0.34	1e-12
BiClassCNN-DNase (9.1%)	-0.02	0.04	0.59	2.1	0.18	7.9e-06
BiClassCNN-Enhancer (2.8%)	0.31	0.18	0.09	3.4	0.25	2.3e-17
BiClassCNN-H3K4me1 (29.6%)	-0.05	0.07	0.50	1.8	0.073	4.3e-18
BiClassCNN-H3K4me3 (7.4%)	0.42	0.13	0.0019	2.9	0.18	8.8e-16
BiClassCNN-Promoter (2.7%)	0.19	0.05	0.00012	2.5	0.16	8.4e-17
BiClassCNN-Repressed (31.8%)	0.24	0.05	2.6e-06	0.68	0.048	1.2e-05
BiClassCNN-SuperEnhancer (8.0%)	-0.03	0.04	0.42	2.2	0.11	1.8e-15
BiClassCNN-TFBS (8.2%)	0.36	0.13	0.0041	2.9	0.21	2.3e-14
BiClassCNN-TSS (1.0%)	0.8	0.13	3.1e-10	6.9	0.61	1e-19
BiClassCNN-UTR-5' (0.5%)	0.017	0.12	0.89	2.9	0.49	1.7e-06
BiClassCNN-WeakEnhancer (1.3%)	-0.15	0.13	0.26	2.3	0.24	1.9e-14

Supplementary Table 31. . **BiClassCNN-Coding, BiClassCNN-Repressed and BiClassCNN-TSS produced independent signals.**. We report standardized effect sizes ($\tau^\star$) and enrichment (E) of the 4 significant BiClassCNN annotations from Figure 1, when modeled jointly and passed through the forward stepwise elimination, all conditioned on the baseline-LD model. The three annotations that remain are BiClassCNN-Coding, BiClassCNN-Repressed and BiClassCNN-TSS. Results are meta-analyzed across 41 traits.

Annotation	$\tau^\star$	se($\tau^\star$)	p($\tau^\star$)	E	se (E)	p(E)
BiClassCNN-Coding (1.3%)	0.73	0.17	2.2e-05	4.9	0.34	1.4e-15
BiClassCNN-Repressed (31.8%)	0.19	0.051	0.00018	0.68	0.048	2.9e-07
BiClassCNN-TSS (1.0%)	0.68	0.13	9.6e-08	6.8	0.6	0.00023

Supplementary Table 32. S-LDSC results for marginal analysis of non-tissue-specific Roadmap and ChromHMM annotations. Standardized Effect sizes ($\tau^\star$) and Enrichment (E) of 8 non-tissue-specific Roadmap and 40 non-tissue-specific ChromHMM annotations. Results are meta-analyzed across 41 traits.

Annotation	$\tau^\star$	se($\tau^\star$)	p($\tau^\star$)	E	se (E)	p(E)
Roadmap-DNase-Avg (2.2%)	0.39	0.11	0.00034	5.3	0.46	9.7e-13
Roadmap-DNase-Max (13.4%)	0.18	0.075	0.02	3	0.23	3.9e-12
Roadmap-H3K27ac-Avg (3.1%)	0.37	0.084	1.1e-05	4.2	0.27	9.4e-17
Roadmap-H3K27ac-Max (21.4%)	0.16	0.079	0.046	2.5	0.13	6.4e-17
Roadmap-H3K4me1-Avg (4.4%)	0.47	0.077	9.1e-10	3.6	0.19	1.9e-17
Roadmap-H3K4me1-Max (25.4%)	0.094	0.099	0.34	2.2	0.1	8.1e-19
Roadmap-H3K4me3-Avg (1.7%)	0.43	0.088	7.2e-07	5.8	0.41	2.7e-18
Roadmap-H3K4me3-Max (6.6%)	-0.12	0.065	0.075	3.3	0.23	5.7e-16
ChromHMM-DNase-Avg (0.7%)	-0.12	0.049	0.012	1.4	0.39	0.4
ChromHMM-DNase-Max (9.2%)	-0.19	0.061	0.0021	1.5	0.24	0.71
ChromHMM-Active-Enhancer-1-Avg (0.3%)	-0.15	0.03	3.2e-07	2.7	0.3	1.2e-08
ChromHMM-Active-Enhancer-1-Max (7.1%)	-0.17	0.044	8.1e-05	2.3	0.22	0.0025
ChromHMM-Active_Enhancer_2-Avg (0.4%)	-0.073	0.052	0.16	2.9	0.34	0.0011
ChromHMM-Active_Enhancer_2-Max (9.9%)	-0.13	0.057	0.027	2.2	0.2	0.011
ChromHMM-Enhancer_acetylation-Avg (0.3%)	-0.19	0.039	8.5e-07	1.4	0.25	0.79
ChromHMM-Enhancer_acetylation-Max (11.3%)	-0.19	0.029	1.8e-10	1.5	0.13	0.0066
ChromHMM-Active_Enhancer_Flanking-Avg (0.5%)	-0.12	0.035	0.00085	2.3	0.22	0.0092
ChromHMM-Active_Enhancer_Flanking-Max (11.1%)	-0.15	0.046	0.00078	1.9	0.15	0.11
ChromHMM-Weak_Enhancer_1-Avg (0.3%)	-0.14	0.035	7.1e-05	1.8	0.31	9.9e-08
ChromHMM-Weak_Enhancer_1-Max (7.7%)	-0.21	0.034	8.8e-10	1.8	0.19	5.1e-06
ChromHMM-Weak_Enhancer_2-Avg (1.2%)	-0.052	0.036	0.15	2.1	0.14	1.5e-10
ChromHMM-Weak_Enhancer_2-Max (19.9%)	-0.12	0.052	0.02	1.7	0.093	5.8e-11
ChromHMM-Heterochromatin-Avg (1.4%)	-0.074	0.02	0.00027	0.3	0.058	2e-18
ChromHMM-Heterochromatin-Max (7.1%)	-0.055	0.018	0.0022	0.39	0.038	3.5e-21
ChromHMM-Promoter_Bivalent-Avg (0.3%)	0.1	0.042	0.017	6.1	0.48	8.2e-14
ChromHMM-Promoter_Bivalent-Max (1.9%)	0.055	0.041	0.18	4	0.27	2.1e-14
ChromHMM-Promoter_Downstream_1-Avg (0.4%)	0.18	0.062	0.0029	7	0.89	7.8e-12
ChromHMM-Promoter_Downstream_1-Max (1.4%)	0.24	0.063	0.0002	6.5	0.6	8.9e-16
ChromHMM-Promoter_Downstream_2-Avg (0.2%)	-0.014	0.045	0.76	2.5	0.67	0.1
ChromHMM-Promoter_Downstream_2-Max (1.5%)	0.095	0.033	0.004	3.6	0.34	2.2e-14
ChromHMM-Promoter_Poised-Avg (0.2%)	-0.14	0.046	0.0018	1.3	0.25	0.074
ChromHMM-Promoter_Poised-Max (6.8%)	-0.23	0.037	1.1e-09	1.2	0.098	0.0011
ChromHMM-Promoter_Upstream-Avg (0.4%)	0.04	0.051	0.43	5.7	0.45	5.3e-16
ChromHMM-Promoter_Upstream-Max (2.5%)	-0.13	0.045	0.0052	3.8	0.34	7.5e-14
ChromHMM-Quiescent-Avg (78.4%)	-0.061	0.018	0.00074	0.73	0.02	3e-18
ChromHMM-Quiescent-Max (91.0%)	-0.086	0.014	1.4e-09	0.86	0.01	6.6e-20
ChromHMM-Repressed_Polycomb-Avg (1.7%)	-0.052	0.016	0.0011	1.5	0.065	1.5e-16
ChromHMM-Repressed_Polycomb-Max (9.9%)	-0.072	0.016	6.5e-06	1.3	0.037	2.9e-20
ChromHMM-TSS-Avg (0.2%)	-0.0011	0.075	0.99	5.1	1	5.1e-05
ChromHMM-TSS-Max (0.9%)	-0.058	0.073	0.42	4.5	0.49	1.7e-10
ChromHMM-Transcription_Enhancer_3'-Avg (0.2%)	0.078	0.027	0.0038	3.4	0.29	1.7e-16
ChromHMM-Transcription_Enhancer_3'-Max (3.1%)	0.088	0.035	0.013	2.8	0.21	6.7e-18
ChromHMM-Transcription Enhancer-5'-Avg (0.4%)	0.12	0.024	9.7e-07	3.6	0.23	5.4e-18
ChromHMM-Transcription_Enhancer_5'-Max (4.8%)	0.16	0.022	2.2e-13	2.9	0.15	6.5e-19
ChromHMM-Transcription_Weak_Enhancer-Avg (0.5%)	0.12	0.024	1.3e-06	2.8	0.16	7.3e-19
ChromHMM-Transcription_Weak_Enhancer-Max (4.7%)	0.16	0.022	3e-12	2.6	0.12	1.7e-21
ChromHMM-Transcriptional_Regulator-Avg (0.3%)	0.27	0.039	5.2e-12	6.8	0.51	4.3e-17
ChromHMM-Transcriptional_Regulator-Max (3.3%)	0.22	0.037	4.2e-09	4	0.26	1.8e-18

Supplementary Table 33. **S-LDSC results for marginal analysis of CpG-island, local CpG-content, local GC-content, pLI, SIFT and Polyphen annotations**. Standardized Effect sizes ($\tau^\star$) and Enrichment (E) of the weighted Coding, TSS, Repressed and Gene $\pm 5KB$ annotations, where the weights may be driven by the underlying sequence context such as CpG-island, LocalCpG-content ($\pm 1kb$), LocalGC-content ($\pm 1kb$) etc as well as pLI, pLI-Coding, pLI-TSS, PolyPhen and SIFT. All analyses are conditioned on baseline-LD annotations. Results are meta-analyzed across 41 traits.

Annotation	$\tau^\star$	se($\tau^\star$)	p($\tau^\star$)	E	se (E)	p(E)
CpGisland (0.8%)	0.2	0.051	6.1e-05	6.7	0.61	5e-14
CpGisland-Coding (0.2%)	0.094	0.049	0.056	7.7	1.2	1.6e-11
CpGisland-Repressed (0.02%)	-0.074	0.023	0.001	3.9	1.4	0.19
CpGisland-TSS (0.4%)	0.23	0.074	0.0019	8.8	0.98	2.2e-11
LocalCpGcontent (5.5%)	-0.19	0.052	0.00025	1.1	0.013	1.5e-15
LocalCpGcontent-Coding (0.2%)	0.44	0.075	4.9e-09	6	0.45	7.6e-16
LocalCpGcontent-Repressed (2.2%)	-0.34	0.058	4.9e-09	0.59	0.055	3.7e-10
LocalCpGcontent-TSS (0.2%)	0.66	0.12	3.8e-08	7.1	0.59	1.1e-17
LocalGCcontent (41.0%)	-0.15	0.03	5.7e-07	1.1	0.0062	1.1e-11
LocalGCcontent-Coding (0.8%)	1.1	0.17	1.9e-10	5	0.35	2e-16
LocalGCcontent-Repressed (18.0%)	-0.68	0.11	5.3e-10	0.63	0.05	1.1e-08
LocalGCcontent-TSS (1.0%)	1.8	0.28	1.7e-10	6.4	0.56	5.7e-19
pLI (14.0%)	0.095	0.011	3.9e-18	1.5	0.034	9.5e-17
pLI-Coding (0.4%)	0.62	0.057	2.5e-27	13	0.78	5.6e-17
pLI-TSS (0.3%)	0.52	0.039	3.5e-41	14	1.1	6.4e-20
Polyphen (0.1%)	0.23	0.081	0.0046	13	2.2	1.8e-10
SIFT (0.1%)	-0.067	0.065	0.3	4.6	1.5	4e-06

Supplementary Table 34. **S-LDSC results for non-tissue-specific variant-level joint model**. Joint S-LDSC model fit of all non-tissue-specific annotations as well as LocalGC-content, LocalCpG-content and CpG-island weighted annotations of TSS, Repressed and Coding regions, and pLI, pLI-TSS, pLI-Coding, SIFT and Polyphen. Only 7 annotations were found to be significant conditioned on all other annotations and the baseline-LD model. Results are meta-analyzed across 41 traits. We also highlight annotations from Figure 1 (below the horizontal line) that were not significant under the joint model

Annotation	$\tau^\star$	se($\tau^\star$)	p($\tau^\star$)	E	se (E)	p(E)
ChromHMM-Active-Enhancer-1-Avg (0.3%)	-0.16	0.03	6.8e-08	2.6	0.29	1.8e-10
Roadmap-H3K4me1-Avg (4.4%)	0.46	0.079	5e-09	3.6	0.19	6.5e-21
pLI-Coding (0.4%)	0.45	0.054	4e-17	11	0.71	3e-16
pLI-TSS (0.3%)	0.35	0.039	1.4e-19	12	1	8.2e-21
LocalGCcontent (41.0%)	-0.16	0.03	1.1e-07	1.1	0.0066	4.2e-17
LocalGCcontent-TSS (1.0%)	1.2	0.28	1.6e-05	5.8	0.52	5.8e-26
LocalGCcontent-Coding (0.8%)	0.91	0.18	7.8e-07	5.2	0.34	6.7e-20
DeepSEAV-H3K4me3-Max (9.4%)	0.13	0.06	0.029	2.2	0.077	6.8e-19
BiClassCNN-Coding (1.3%)	0.43	0.28	0.13	5.1	0.34	6.5e-19
BiClassCNN-Promoter (2.0%)	0.14	0.048	0.0036	2.7	0.17	2.2e-20
BiClassCNN-Repressed (31.8%)	0.1	0.043	0.02	0.66	0.046	1.1e-08
BiClassCNN-TSS (1.0%)	0.19	0.25	0.44	6.2	0.55	3.9e-18

Supplementary Table 35. The Roadmap-H3K4me1-Avg annotation was sufficient to eliminate the DeepSEAV-H3K4me3-Max annotation that was significant in marginal analysis. Standardized Effect sizes ($\tau^\star$) and Enrichment (E) of the significant non-tissue-specific variant-level DeepSEAV and Roadmap annotations from marginal analysis, when modeled conditioned on each other and the 86 baseline-LD annotations, and passed through Forward Stepwise Elimination. The results for the penultimate step of the elimination process is shown. DeepSEAV-H3K4me3-Max is not significant after correcting for all tested hypothesis (0.05/106) and gets eliminated next. Results are meta-analyzed across 41 traits.

Annotation	$\tau^\star$	se($\tau^\star$)	p($\tau^\star$)	E	se (E)	p(E)
Roadmap-H3K4me1-Avg (4.4%)	0.455	0.08	1e-08	3.63	0.19	3e-19
DeepSEAV-H3K4me3-Max (9.4%)	0.182	0.06	0.004	2.14	0.08	1e-16

Supplementary Table 36. The Local GC-content annotations were sufficient to eliminate the 4 BiClassCNN annotations that were significant in our marginal analysis. Meta-analyzed $\tau^\star$ and Enrichment E of the Joint S-LDSC model fit of the 4 BiClassCNN annotations from Figure 1 conditioned on the 86 baseline-LD annotations and 3 LocalGCcontent annotations from Table 34. Results are meta-analyzed across 41 traits.

Annotation	$\tau^\star$	se($\tau^\star$)	p($\tau^\star$)	E	se (E)	p(E)
BiClassCNN-Coding (1.3%)	0.69	0.3	0.023	5	0.35	1e-16
BiClassCNN-Promoter (2.7%)	0.15	0.052	0.0039	2.7	0.17	3.1e-20
BiClassCNN-Repressed (31.8%)	0.1	0.045	0.022	0.69	0.047	1.9e-07
BiClassCNN-TSS (1.0%)	0.13	0.28	0.65	6.6	0.58	3e-19

Supplementary Table 37. **AUROC of various sets of annotations incorporating non-tissue-specific variant-level deep learning annotations in predicting** $12,296$ **NIH GWAS SNPs**. We report the AUROC for a gradient boosting model trained using each respective set of annotations, either without including baseline-LD model annotations; including baseline-LD model annotations; or including annotations from the non-tissue-specific variant-level joint model (baseline-LD model + 7 annotations; baseline-LD+7).

Feature	GWAS SNPs
DeepSEAV-Avg/Max	0.582
BasenjiV-Avg/Max	0.612
DeepSEAV-All	0.591
BasenjiV-All	0.620
baseline-LD	0.758
baseline-LD + DeepSEAV-Avg/Max	0.759
baseline-LD + BasenjiV-Avg/Max	0.766
baseline-LD + DeepSEAV-All	0.765
baseline-LD + BasenjiV-All	0.770
baseline-LD+7	0.762
baseline-LD+7 + DeepSEAV-Avg/Max	0.764
baseline-LD+7 + BasenjiV-Avg/Max	0.765
baseline-LD+7 + DeepSEAV-All	0.769
baseline-LD+7 + BasenjiV-All	0.772

Supplementary Table 38. **Enrichment of NIH GWAS SNPs for non-tissue-specific variant-level annotations**. We report the enrichment in deep learning annotations (along with Jackknife standard error) at top 12, 296 known disease-associated SNPs from NIH GWAS catalog[12].

Feature	GWAS SNPs
BasenjiV-DNase-Avg	1.61 (0.010)
BasenjiV-DNase-Max	1.35 (0.005)
BasenjiV-H3K27ac-Avg	1.62 (0.009)
BasenjiV-H3K27ac-Max	1.42 (0.005)
BasenjiV-H3K4me1-Avg	1.48 (0.005)
BasenjiV-H3K4me1-Max	1.31 (0.003)
BasenjiV-H3K4me3-Avg	1.81 (0.016)
BasenjiV-H3K4me3-Max	1.30 (0.006)
DeepSEAV-DNase-Avg	1.58 (0.009)
DeepSEAV-DNase-Max	1.39 (0.004)
DeepSEAV-H3K27ac-Avg	1.58 (0.008)
DeepSEAV-H3K27ac-Max	1.39 (0.004)
DeepSEAV-H3K4me1-Avg	1.46 (0.001)
DeepSEAV-H3K4me1-Max	1.31 (0.003)
DeepSEAV-H3K4me3-Avg	1.81 (0.013)
DeepSEAV-H3K4me3-Max	1.43 (0.005)

Supplementary Table 39. **S-LDSC results for marginal analysis of blood-specific variant-level DeepSEAV and BasenjiV annotations**. Standardized Effect sizes ($\tau^\star$) and Enrichment (E) of 8 blood-specific variant-level DeepSEAV and 8 blood-specific variant-level BasenjiV annotations, when conditioned on non-tissue-specific variant-level joint model (baseline-LD and annotations from Supplementary Figure 6). Results are meta-analyzed across 11 blood-related traits.

Annotation	$\tau^\star$	se($\tau^\star$)	p($\tau^\star$)	E	se (E)	p(E)
BasenjiV-DNase-blood-Avg (1.9%)	0.06	0.11	0.6	5	0.37	1.2e-12
BasenjiV-DNase-blood-Max (5.4%)	0.22	0.1	0.028	3.5	0.2	8.8e-06
BasenjiV-H3K27ac-blood-Avg (2.5%)	0.59	0.13	4.2e-06	4.6	0.25	3.2e-05
BasenjiV-H3K27ac-blood-Max (8.5%)	0.38	0.13	0.0045	3.2	0.13	4.5e-05
BasenjiV-H3K4me1-blood-Avg (6.2%)	0.77	0.13	4.4e-09	3.2	0.15	3.7e-05
BasenjiV-H3K4me1-blood-Max (13.7%)	0.58	0.13	9.8e-06	2.6	0.095	6.4e-05
BasenjiV-H3K4me3-blood-Avg (2.0%)	0.34	0.13	0.011	6.9	0.41	5.8e-05
BasenjiV-H3K4me3-blood-Max (5.5%)	0.34	0.098	0.00065	4.2	0.18	6.7e-05
DeepSEAV-DNase-blood-Avg (1.9%)	-0.12	0.13	0.35	4.9	0.36	6e-05
DeepSEAV-DNAse-blood-Max (5.4%)	0.055	0.11	0.6	3.4	0.18	6.9e-05
DeepSEAV-H3K27ac-blood-Avg (2.5%)	0.12	0.11	0.29	4	0.21	9.1e-05
DeepSEAV-H3K27ac-blood-Max (8.5%)	0.14	0.11	0.2	2.8	0.1	9e-05
DeepSEAV-H3K4me1-blood-Avg (6.2%)	0.14	0.086	0.093	2.8	0.11	0.0001
DeepSEAV-H3K4me1-blood-Max (13.7%)	0.14	0.073	0.048	2.4	0.074	0.0001
DeepSEAV-H3K4me3-blood-Avg (2.0%)	-0.25	0.13	0.052	5.8	0.42	4.1e-05
DeepSEAV-H3K4me3-blood-Max (5.5%)	0.064	0.096	0.5	3.9	0.19	8.3e-05

Supplementary Table 40. S-LDSC results for marginal analysis of blood-specific Roadmap and ChromHMM annotations. Standardized Effect sizes ($\tau^\star$) and Enrichment (E) of 8 blood-specific Roadmap and 40 blood-specific ChromHMM annotations, when conditioned on non-tissue-specific variant-level joint model (baseline-LD and annotations from Supplementary Figure 6). Results are meta-analyzed across 11 blood-related traits.

Annotation	$\tau^\star$	se($\tau^\star$)	p($\tau^\star$)	E	se (E)	p(E)
Roadmap-DNase-blood-Avg (2.1%)	1.8	0.29	1.6e-09	14	1.2	7.1e-06
Roadmap-DNase-blood-Max (6.2%)	1.4	0.22	9.8e-10	8.7	0.68	1e-05
Roadmap-H3K27ac-blood-Avg (3.2%)	1.5	0.32	2e-06	9.5	0.77	4.9e-06
Roadmap-H3K27ac-blood-Max (10.2%)	1.3	0.21	1.7e-10	6.4	0.43	9.1e-06
Roadmap-H3K4me1-blood-Avg (4.9%)	1.5	0.33	7.3e-06	7.1	0.54	6.4e-06
Roadmap-H3K4me1-blood-Max (11.2%)	1.4	0.24	5.7e-09	5.8	0.42	2.8e-06
Roadmap-H3K4me3-blood-Avg (1.8%)	1.3	0.3	1.5e-05	13	1.1	1e-05
Roadmap-H3K4me3-blood-Max (3.6%)	1.4	0.23	3.6e-09	11	0.86	2.9e-06
ChromHMM-DNase-blood-Avg (0.4%)	-0.017	0.11	0.88	2.9	0.91	0.0015
ChromHMM-DNase-blood-Max (2.2%)	0.37	0.099	0.00019	5.2	0.59	3e-07
ChromHMM-Active_Enhancer_1-blood-Avg (0.3%)	1	0.25	3.4e-05	12	2.2	2.1e-05
ChromHMM-Active_Enhancer_1-blood-Max (2.6%)	1	0.17	7.7e-09	9.3	1.1	1.7e-07
ChromHMM-Active_Enhancer_2-blood-Avg (0.4%)	1.1	0.26	2.1e-05	12	1.8	6.4e-07
ChromHMM-Active_Enhancer_2-blood-Max (3.5%)	1.2	0.22	3.3e-08	9	1.1	3.3e-08
ChromHMM-Enhancer_acetylation-blood-Avg (0.2%)	0.24	0.13	0.059	5	0.9	4.9e-06
ChromHMM-Enhancer_acetylation-blood-Max (2.8%)	0.54	0.14	8.3e-05	5.6	0.72	9.5e-06
ChromHMM-Active_Enhancer_Flanking-blood-Avg (0.4%)	0.96	0.24	4.6e-05	10	1.6	1.9e-06
ChromHMM-Active_Enhancer_Flanking-blood-Max (3.3%)	1.1	0.22	1.1e-06	8.2	1.1	2.3e-07
ChromHMM-Weak_Enhancer_1-blood-Avg (0.3%)	0.43	0.11	5.3e-05	8.8	1	7.4e-08
ChromHMM-Weak_Enhancer_1-blood-Max (2.2%)	0.78	0.14	1.4e-08	8.6	0.89	9.7e-06
ChromHMM-Weak_Enhancer_2-blood-Avg (1.1%)	0.81	0.19	1.3e-05	6.6	0.88	9e-07
ChromHMM-Weak_Enhancer_2-blood-Max (6.5%)	1	0.19	4.1e-08	5.8	0.62	1.6e-07
ChromHMM-Heterochromatin-blood-Avg (1.2%)	-0.13	0.031	3.2e-05	0.12	0.16	0.00025
ChromHMM-Heterochromatin-blood-Max (3.2%)	-0.097	0.031	0.002	0.01	0.13	0.00057
ChromHMM-Promoter_Bivalent-blood-Avg (0.3%)	-0.34	0.12	0.0053	2.1	1.5	0.08
ChromHMM-Promoter_Bivalent-blood-Max (0.8%)	-0.078	0.13	0.54	4.7	0.96	1.3e-05
ChromHMM-Promoter_Downstream_1-blood-Avg (0.4%)	0.66	0.18	0.00034	19	2.1	1.4e-05
ChromHMM-Promoter_Downstream_1-blood-Max (0.9%)	1.1	0.18	3.2e-10	19	1.5	8.7e-06
ChromHMM-Promoter_Downstream_2-blood-Avg (0.1%)	0.51	0.13	0.0001	14	2.2	7.7e-08
ChromHMM-Promoter_Downstream_2-blood-Max (0.7%)	0.65	0.11	1.4e-08	12	1.5	3.8e-11
ChromHMM-Promoter_Poised-blood-Avg (0.2%)	-0.12	0.12	0.32	2.8	1.4	0.0029
ChromHMM-Promoter_Poised-blood-Max (1.6%)	0.065	0.075	0.38	3.6	0.53	2.5e-05
ChromHMM-Promoter_Upstream-blood-Avg (0.5%)	-0.15	0.12	0.22	8.7	1.2	6.9e-08
ChromHMM-Promoter_Upstream-blood-Max (1.3%)	0.59	0.13	1e-05	12	0.86	2.1e-05
ChromHMM-Quiescent-blood-Avg (80.0%)	-0.21	0.082	0.012	0.52	0.022	4.9e-05
ChromHMM-Quiescent-blood-Max (86.0%)	-0.092	0.049	0.06	0.64	0.017	6e-05
ChromHMM-Repressed_Polycomb-blood-Avg (1.4%)	-0.17	0.047	0.00037	1.1	0.36	0.089
ChromHMM-Repressed_Polycomb-blood-Max (0.04%)	-0.12	0.04	0.0038	1.3	0.22	0.0036
ChromHMM-TSS-blood-Avg (0.1%)	-0.35	0.15	0.022	8.9	3.9	0.0041
ChromHMM-TSS-blood-Max (0.3%)	-0.34	0.12	0.0051	7.5	2	0.00049
ChromHMM-Transcription_Enhancer_3'-blood-Avg (0.2%)	0.29	0.11	0.0067	7.7	1.4	1.4e-06
ChromHMM-Transcription_Enhancer_3'-blood-Max (1.1%)	0.45	0.11	2.9e-05	7.6	0.95	4.2e-06
ChromHMM-Transcription_Enhancer_5'-blood-Avg (0.5%)	0.28	0.11	0.0085	6.2	0.96	2.6e-10
ChromHMM-Transcription_Enhancer_5'-blood-Max (2.5%)	0.45	0.11	5.4e-05	5.8	0.68	1.4e-06
ChromHMM-Transcription_Weak_Enhancer-blood-Avg (0.6%)	-0.035	0.1	0.72	3.1	0.87	6.9e-07
ChromHMM-Transcription_Weak_Enhancer-blood-Max (2.6%)	0.19	0.09	0.035	3.9	0.64	6.8e-10
ChromHMM-Transcriptional_Regulator-blood-Avg (0.4%)	0.76	0.11	2.8e-12	14	1.3	1.3e-10
ChromHMM-Transcriptional_Regulator-blood-Max (1.7%)	0.74	0.16	2.6e-06	9.6	1.1	2.4e-11

Supplementary Table 41. **S-LDSC results for blood-specific variant-level joint model**. Standardized Effect sizes ($\tau^{\star}$) and Enrichment (E) of the significant blood-specific variant-level DeepSEAV, BasenjiV, Roadmap and ChromHMM annotations from Table 39 and Table 40, conditioned on each other and the non-tissue-specific variant-level joint model (baseline-LD and annotations from Supplementary Figure 6). Results are meta-analyzed across 11 blood-related traits.

Annotation	$\tau^{\star}$	se($\tau^{\star}$)	p($\tau^{\star}$)	E	se (E)	p(E)
ChromHMM-Active_Enhancer_2-blood-Max (3.5%)	0.73	0.18	6.9e-05	7.5	0.89	1.7e-06
ChromHMM-Promoter_Downstream_1-blood-Max (1.1%)	1	0.21	1.4e-06	15	1.5	3.5e-05
ChromHMM-Transcriptional_Regulator-blood-Avg (0.4%)	0.84	0.13	1.6e-11	12	1.1	7e-09
ChromHMM-Repressed_Polycomb-blood-Avg (1.4%)	-0.11	0.032	5.8e-04	1.5	0.35	0.0026
Roadmap-H3K4me1-blood-Max (11.2%)	0.68	0.18	0.00023	5.4	0.39	6.9e-06
Roadmap-H3K4me3-blood-Avg (1.8%)	-0.77	0.19	7.4e-05	9.4	0.71	3.8e-05

Supplementary Table 42. The 2 surviving blood-specific Roadmap annotations were sufficient to eliminate 3 of the 4 blood-specific variant-level BasenjiV annotations that were significant in marginal analysis. Joint S-LDSC model fit of the blood-specific variant-level BasenjiV annotations from Table 39 conditioned on the non-tissue-specific variant-level joint model plus the two Roadmap blood annotations from Table 41. Results are meta-analyzed across 11 blood-related traits.

Annotation	$\tau^{\star}$	se($\tau^{\star}$)	p($\tau^{\star}$)	E	se (E)	p(E)
BasenjiV-H3K27ac-blood-Avg (2.5%)	0.16	0.12	0.17	4.4	0.23	2e-05
BasenjiV-H3K4me1-blood-Avg (6.2%)	0.34	0.094	0.00031	3.1	0.12	2.5e-05
BasenjiV-H3K4me1-blood-Max (13.7%)	0.18	0.11	0.096	2.5	0.086	3.9e-05
BasenjiV-H3K4me3-blood-Max (5.5%)	-0.052	0.1	0.61	4.1	0.19	3.1e-05

Supplementary Table 43. **AUROC of various sets of annotations incorporating blood-specific variant-level deep learning annotations in predicting 8,741 fine-mapped autoimmune diseease SNPs**.We report the AUROC for a gradient boosting model trained using each respective set of annotations, either without including baseline-LD model annotations; including baseline-LD model annotations; including annotations from the non-tissue-specific variant-level joint model (baseline-LD model + 7 annotations; baseline-LD+7); or including annotations from the blood-specific joint model (non-tissue-specific joint variant-level joint model + 6 blood annotations; baseline-LD+7+6Blood).

Feature	finemapped SNPs
DeepSEAV-blood-Avg/Max	0.621
BasenjiV-blood-Avg/Max	0.664
DeepSEAV-blood-All	0.631
BasenjiV-blood-All	0.671
baseline-LD	0.841
baseline-LD + DeepSEAV-blood-Avg/Max	0.846
baseline-LD + BasenjiV-blood-Avg/Max	0.852
baseline-LD + DeepSEAV-blood-All	0.849
baseline-LD + BasenjiV-blood-All	0.854
baseline-LD+7	0.845
baseline-LD+7 + DeepSEAV-blood-Avg/Max	0.849
baseline-LD+7 + BasenjiV-blood-Avg/Max	0.853
baseline-LD+7 + DeepSEAV-blood-All	0.848
baseline-LD+7 + BasenjiV-blood-All	0.853
baseline-LD+7+6Blood	0.851
baseline-LD+7+6Blood + DeepSEAV-blood-Avg/Max	0.853
baseline-LD+7+6Blood + BasenjiV-blood-Avg/Max	0.854
baseline-LD+7+6Blood + DeepSEAV-blood-All	0.853
baseline-LD+7+6Blood + BasenjiV-blood-All	0.853

Supplementary Table 44. **Enrichment of fine-mapped autoimmune disease SNPs for blood-specific variant-level annotations**. We report the enrichment in blood-specific variant-level deep learning annotations (along with Jackknife standard error) for 8741 fine-mapped SNPs in immune-related traits[17].

Feature	finemapped SNPs
BasenjiV-DNase-blood-Avg	2.02 (0.03)
BasenjiV-DNase-blood-Max	1.80 (0.03)
BasenjiV-H3K27ac-blood-Avg	2.14 (0.02)
BasenjiV-H3K27ac-blood-Max	1.85 (0.02)
BasenjiV-H3K4me1-blood-Avg	2.04 (0.03)
BasenjiV-H3K4me1-blood-Max	1.81 (0.02)
BasenjiV-H3K4me3-blood-Avg	2.17 (0.02)
BasenjiV-H3K4me3-blood-Max	1.95 (0.03)
DeepSEAV-DNase-blood-Avg	1.88 (0.03)
DeepSEAV-DNase-blood-Max	1.68 (0.02)
DeepSEAV-H3K27ac-blood-Avg	1.81 (0.02)
DeepSEAV-H3K27ac-blood-Max	1.67 (0.02)
DeepSEAV-H3K4me1-blood-Avg	1.70 (0.01)
DeepSEAV-H3K4me1-blood-Max	1.55 (0.02)
DeepSEAV-H3K4me3-blood-Avg	1.95 (0.02)
DeepSEAV-H3K4me3-blood-Max	1.76 (0.02)

Supplementary Table 45. **S-LDSC results for marginal analysis of brain-specific variant-level DeepSEAV and BasenjiV annotations**. Standardized Effect sizes ($\tau^\star$) and Enrichment (E) of 8 brain-specific variant-level DeepSEAV and 8 brain-specific variant-level BasenjiV annotations corresponding to 4 chromatin marks, DNase-1 Hypersensitivity Sites (DHS), H3K27ac, H3K4me1 and H3K4me3, when conditioned on the non-tissue-specific variant-level joint model (baseline-LD and annotations from Supplementary Figure 6). Results are meta-analyzed across 8 brain-related traits..

Annotation	$\tau^\star$	se($\tau^\star$)	p($\tau^\star$)	E	se (E)	p(E)
DeepSEAV-DNase-brain-Avg (2.9%)	0.5	0.081	8.6e-10	2.9	0.23	5e-05
DeepSEAV-DNase-brain-Max (4.5%)	0.41	0.067	1.7e-09	2.5	0.19	3.4e-05
DeepSEAV-H3K27ac-brain-Avg (3.2%)	0.6	0.066	5.4e-20	2.3	0.072	1.1e-08
DeepSEAV-H3K27ac-brain-Max (7.6%)	0.55	0.062	1.5e-18	2.1	0.076	8.8e-09
DeepSEAV-H3K4me1-brain-Avg (4.7%)	0.37	0.06	5.8e-10	2.1	0.06	3.7e-11
DeepSEAV-H3K4me1-brain-Max (10.5%)	0.36	0.053	5.3e-12	1.9	0.07	2.6e-11
DeepSEAV-H3K4me3-brain-Avg (1.9%)	0.77	0.095	4.6e-16	4.4	0.26	1.7e-08
DeepSEAV-H3K4me3-brain-Max (3.4%)	0.77	0.085	1e-19	3.8	0.19	2.2e-08
BasenjiV-DNase-brain-Avg (2.9%)	0.47	0.078	1.3e-09	2.7	0.16	8.8e-08
BasenjiV-DNase-brain-Max (4.5%)	0.49	0.076	9.3e-11	2.8	0.18	1.2e-07
BasenjiV-H3K27ac-brain-Avg (3.2%)	0.34	0.049	3.1e-12	2.3	0.079	1.6e-08
BasenjiV-H3K27ac-brain-Max (7.6%)	0.4	0.058	3.6e-12	2.3	0.12	1.7e-06
BasenjiV-H3K4me1-brain-Avg (4.7%)	0.37	0.059	4.1e-10	2.1	0.059	7.6e-11
BasenjiV-H3K4me1-brain-Max (10.5%)	0.33	0.09	0.00022	2	0.062	2.6e-10
BasenjiV-H3K4me3-brain-Avg (1.9%)	0.55	0.074	1.1e-13	3.6	0.18	5.2e-07
BasenjiV-H3K4me3-brain-Max (3.4%)	0.77	0.089	8.2e-18	4	0.3	8.8e-06

Supplementary Table 46. S-LDSC for marginal analysis of brain-specific Roadmap and ChromHMM annotations. Standardized Effect sizes ($\tau^\star$) and Enrichment (E) of 8 brain-specific Roadmap and 40 brain-specific ChromHMM annotations, when conditioned on the non-tissue-specific variant-level joint model annotations (baseline-LD and annotations from Supplementary Figure 6). Results are meta-analyzed across 8 brain-related traits.

Annotation	$\tau^\star$	se($\tau^\star$)	p($\tau^\star$)	E	se (E)	p(E)
Roadmap-DNase-brain-Avg (2.4%)	0.55	0.084	5.5e-11	4.9	0.41	5.4e-06
Roadmap-DNase-brain-Max (5.1%)	0.39	0.079	7.1e-07	3.5	0.21	2.4e-06
Roadmap-H3K27ac-brain-Avg (3.2%)	0.34	0.09	0.00019	3.5	0.15	1.1e-10
Roadmap-H3K27ac-brain-Max (8.2%)	0.37	0.095	0.00012	2.9	0.11	7.6e-11
Roadmap-H3K4me1-brain-Avg (4.4%)	0.48	0.12	5e-05	3.2	0.12	4.4e-11
Roadmap-H3K4me1-brain-Max (11.2%)	0.46	0.12	0.00016	2.7	0.09	3.6e-11
Roadmap-H3K4me3-brain-Avg (1.5%)	0.86	0.15	7.7e-09	6.8	0.37	1.1e-12
Roadmap-H3K4me3-brain-Max (2.9%)	0.72	0.12	1.8e-09	5.7	0.28	4e-12
ChromHMM-DNase-brain-Avg (0.8%)	-0.12	0.096	0.21	0.86	0.8	0.45
ChromHMM-DNase-brain-Max (3.0%)	0.062	0.072	0.39	1.8	0.5	0.0031
ChromHMM-Active-Enhancer-1-brain-Avg (0.4%)	0.24	0.086	0.0061	3.6	0.5	0.06
ChromHMM-Active-Enhancer-1-brain-Max (1.5%)	0.32	0.1	0.002	3.5	0.45	0.056
ChromHMM-Active_Enhancer_2-brain-Avg (0.4%)	0.39	0.083	3.4e-06	4.8	0.48	0.00043
ChromHMM-Active_Enhancer_2-brain-Max (2.2%)	0.36	0.082	8e-06	4	0.3	1e-05
ChromHMM-Enhancer_acetylation-brain-Avg (0.3%)	0.13	0.054	0.02	2.3	0.29	0.077
ChromHMM-Enhancer_acetylation-brain-Max (2.2%)	0.19	0.068	0.0047	2.5	0.29	0.016
ChromHMM-Active_Enhancer_Flanking-brain-Avg (0.8%)	0.13	0.057	0.024	2.5	0.21	0.0031
ChromHMM-Active_Enhancer_Flanking-brain-Max (3.1%)	0.16	0.062	0.011	2.3	0.17	0.002
ChromHMM-Weak_Enhancer_1-brain-Avg (0.4%)	0.19	0.073	0.0087	3.9	0.56	0.03
ChromHMM-Weak_Enhancer_1-brain-Max (1.7%)	0.29	0.091	0.0012	4.1	0.51	0.014
ChromHMM-Weak_Enhancer_2-brain-Avg (1.3%)	0.25	0.074	0.00065	2.8	0.27	3.5e-10
ChromHMM-Weak_Enhancer_2-brain-Max (5.6%)	0.27	0.085	0.0015	2.5	0.23	3.6e-10
ChromHMM-Heterochromatin-brain-Avg (1.2%)	-0.00042	0.021	0.98	0.37	0.12	0.016
ChromHMM-Heterochromatin-brain-Max (2.3%)	0.0051	0.021	0.81	0.42	0.1	0.0058
ChromHMM-Promoter_Bivalent-brain-Avg (0.2%)	0.15	0.06	0.01	7.4	0.97	0.00052
ChromHMM-Promoter_Bivalent-brain-Max (0.6%)	0.29	0.058	3e-07	7.2	0.57	2.7e-05
ChromHMM-Promoter_Downstream_1-brain-Avg (0.4%)	0.29	0.072	7e-05	7.7	0.9	1.5e-05
ChromHMM-Promoter_Downstream_1-brain-Max (0.6%)	0.47	0.077	1.2e-09	9.1	0.83	1.9e-08
ChromHMM-Promoter_Downstream_2-brain-Avg (0.2%)	0.12	0.069	0.075	4.4	1.2	0.095
ChromHMM-Promoter_Downstream_2-brain-Max (0.4%)	0.22	0.068	0.0011	5.5	0.98	0.00026
ChromHMM-Promoter_Poised-brain-Avg (0.2%)	0.23	0.083	0.0064	4.3	0.81	0.059
ChromHMM-Promoter_Poised-brain-Max (1.1%)	0.24	0.1	0.018	3.7	0.75	0.11
ChromHMMM-Promoter_Upstream-brain-Avg (0.5%)	0.46	0.11	2.7e-05	9.6	0.82	1e-10
ChromHMM-Promoter_Upstream-brain-Max (1.0%)	0.5	0.11	5.9e-06	8.1	0.6	1.3e-11
ChromHMM-Quiescent-brain-Avg (77.6%)	-0.22	0.054	5.1e-05	0.81	0.013	8.1e-07
ChromHMM-Quiescent-brain-Max (86.0%)	-0.088	0.029	0.0025	0.87	0.0098	3.4e-06
ChromHMM-Repressed_Polycomb-brain-Avg (1.4%)	-0.069	0.025	0.005	1	0.16	0.31
ChromHMM-Repressed_Polycomb-brain-Max (4.3%)	-0.046	0.024	0.054	1.2	0.094	0.012
ChromHMM-TSS-brain-Avg (0.2%)	0.32	0.092	0.00048	7.9	1.6	0.00061
ChromHMM-TSS-brain-Max (0.4%)	0.44	0.097	5e-06	9	1.3	0.0017
ChromHMM-Transcription_Enhancer_3'-brain-Avg (0.3%)	0.12	0.038	0.0027	3.6	0.39	0.039
ChromHMM-Transcription_Enhancer_3'-brain-Max (1.0%)	0.092	0.039	0.017	2.9	0.31	0.0022
ChromHMM-Transcription_Enhancer_5'-brain-Avg (0.5%)	0.027	0.032	0.4	2.8	0.24	1.7e-08
ChromHMM-Transcription_Enhancer_5'-brain-Max (1.6%)	0.083	0.034	0.016	2.8	0.2	5.4e-10
ChromHMM-Transcription_Weak_Enhancer-brain-Avg (0.6%)	0.21	0.049	2.8e-05	3.3	0.33	0.00026
ChromHMM-Transcription_Weak_Enhancer-brain-Max (1.8%)	0.22	0.05	1.7e-05	3	0.25	2.2e-11
ChromHMM-Transcriptional_Regulator-brain-Avg (0.3%)	0.11	0.045	0.019	5	0.52	1.8e-06
ChromHMM-Transcriptional_Regulator-brain-Max (0.9%)	0.056	0.047	0.24	3.7	0.35	3.3e-06

Supplementary Table 47. **S-LDSC results for brain-specific variant-level joint model**. Standardized Effect sizes ($\tau^{\star}$) and Enrichment (E) of the significant brain-specific variant-level DeepSEAV, BasenjiV, Roadmap and ChromHMM annotations from Table 45 and Table 46, conditioned on each other and the non-tissue-specific variant-level joint model annotations (baseline-LD and annotations from Supplementary Figure 6). Results are meta-analyzed across 8 brain-related traits.

Annotation	$\tau^{\star}$	se($\tau^{\star}$)	p($\tau^{\star}$)	E	se (E)	p(E)
BasenjiV-H3K27ac-brain-Max (7.7%)	0.27	0.059	5.4e-06	2.3	0.077	1.3e-08
ChromHMM-Promoter_Bivalent-brain-Max (0.6%)	0.21	0.056	0.00014	6.9	0.56	3.3e-06
ChromHMM-Transcriptional-Weak-Enhancer-brain-Max (1.8%)	0.26	0.06	1.2e-05	3	0.25	4.8e-16
ChromHMM-Weak_Enhancer_2-brain-Avg (1.3%)	0.27	0.078	0.00067	2.7	0.25	3.4e-12
DeepSEAV-H3K4me3-brain-Max (3.4%)	0.35	0.09	0.0001	3.6	0.2	5.8e-08
Roadmap-H3K4me3-brain-Max (2.9%)	0.4	0.091	1.3e-05	5.2	0.28	1.5e-10

Supplementary Table 48. The surviving brain-specific Roadmap annotations do not eliminate any brain specific variant-level DeepSEAV and brain-specific variant-level BasenjiV annotations that were significant in marginal analysis. Standardized Effect sizes ($\tau^{\star}$) and Enrichment (E) of brain-specific DeepSEAV and brain-specific BasenjiV annotations, conditioned on the non-tissue-specific variant-level joint model annotations and 1 significant Roadmap brain annotation (Roadmap-H3K4me3-brain-Max) that is significant in the joint model in Table 47. Results are meta-analyzed across 8 brain-related traits.

Annotation	$\tau^{\star}$	se($\tau^{\star}$)	p($\tau^{\star}$)	E	se (E)	p(E)
DeepSEAV-DNase-brain-Avg (2.9%)	0.31	0.081	0.00011	2.8	0.24	3.8e-08
DeepSEAV-DNase-brain-Max (4.5%)	0.25	0.069	0.00022	2.5	0.2	2.3e-08
DeepSEAV-H3K27ac-brain-Avg (3.2%)	0.47	0.068	2.4e-12	2.4	0.072	9e-12
DeepSEAV-H3K27ac-brain-Max (7.7%)	0.43	0.064	1.7e-11	2.2	0.072	5.6e-12
DeepSEAV-H3K4me1-brain-Avg (4.7%)	0.3	0.054	1.6e-08	2.1	0.057	4.1e-13
DeepSEAV-H3K4me1-brain-Max (10.5%)	0.29	0.053	4.9e-08	1.9	0.068	5e-13
DeepSEAV-H3K4me3-brain-Avg (1.9%)	0.5	0.092	5.7e-08	4.4	0.26	1.3e-11
DeepSEAV-H3K4me3-brain-Max (3.4%)	0.54	0.087	6.4e-10	3.8	0.19	6.7e-12
BasenjiV-DNase-brain-Avg (2.9%)	0.34	0.064	8.7e-08	2.8	0.17	1.4e-10
BasenjiV-DNase-brain-Max (4.5%)	0.36	0.068	1.4e-07	2.7	0.15	1.5e-10
BasenjiV-H3K27ac-brain-Avg (3.2%)	0.25	0.055	4e-06	2.4	0.11	1.3e-10
BasenjiV-H3K27ac-brain-Max (7.7%)	0.34	0.056	1.1e-09	2.3	0.073	1.3e-10
BasenjiV-H3K4me1-brain-Avg (4.7%)	0.3	0.053	2.2e-08	2.1	0.059	7e-12
BasenjiV-H3K4me1-brain-Max (10.5%)	0.29	0.087	0.00077	2.1	0.061	1.6e-12
BasenjiV-H3K4me3-brain-Avg (1.9%)	0.31	0.081	0.00014	4.1	0.28	4.8e-11
BasenjiV-H3K4me3-brain-Max (3.4%)	0.61	0.08	2.3e-14	3.8	0.17	3.5e-11

Supplementary Table 49. The surviving brain-specific ChromHMM annotations do not eliminate any brain specific variant-level DeepSEAV and brain-specific variant-level BasenjiV annotations that were significant in marginal analysis. Standardized Effect sizes ($\tau^\star$) and Enrichment (E) of brain-specific variant-level DeepSEAV and brain-specific variant-level BasenjiV annotations, conditioned on the non-tissue-specific variant-level joint model annotations and 3 significant ChromHMM brain annotations that are significant in the joint model in Table 47. Results are meta-analyzed across 8 brain-related traits.

Annotation	$\tau^\star$	se($\tau^\star$)	p($\tau^\star$)	E	se (E)	p(E)
DeepSEAV-DNase-brain-Avg (2.9%)	0.44	0.079	2e-08	2.8	0.23	3.5e-06
DeepSEAV-DNase-brain-Max (4.5%)	0.36	0.067	5.9e-08	2.5	0.2	1.3e-06
DeepSEAV-H3K27ac-brain-Avg (3.2%)	0.55	0.065	2.6e-17	2.2	0.085	7.1e-08
DeepSEAV-H3K27ac-brain-Max (7.6%)	0.5	0.062	5.3e-16	2.1	0.086	5.4e-08
DeepSEAV-H3K4me1-brain-Avg (4.7%)	0.28	0.053	9.9e-08	1.9	0.07	4.4e-08
DeepSEAV-H3K4me1-brain-Max (10.5%)	0.28	0.052	5.5e-08	1.8	0.08	2.3e-08
DeepSEAV-H3K4me3-brain-Avg (1.9%)	0.73	0.094	8.4e-15	4.2	0.26	5.4e-08
DeepSEAV-H3K4me3-brain-Max (3.4%)	0.72	0.086	3.9e-17	3.6	0.2	6.3e-08
BasenjiV-DNase-brain-Avg (2.9%)	0.4	0.071	1.7e-08	2.7	0.18	7.2e-08
BasenjiV-DNase-brain-Max (4.5%)	0.42	0.073	5.4e-09	2.7	0.16	5.1e-08
BasenjiV-H3K27ac-brain-Avg (3.2%)	0.30	0.05	1.6e-09	2.2	0.13	1.1e-07
BasenjiV-H3K27ac-brain-Max (7.6%)	0.36	0.057	2.3e-10	2.2	0.084	5.7e-08
BasenjiV-H3K4me1-brain-Avg (4.7%)	0.31	0.057	6.1e-08	2	0.072	6.9e-08
BasenjiV-H3K4me1-brain-Max (10.5%)	0.30	0.089	0.00061	2	0.054	4e-09
BasenjiV-H3K4me3-brain-Avg (1.9%)	0.52	0.073	1e-12	3.8	0.31	2.4e-07
BasenjiV-H3K4me3-brain-Max (3.4%)	0.68	0.096	1.5e-12	3.5	0.2	1.4e-07

Supplementary Table 50. **Weighted k-mer enrichments for significant variant level brain-specific variant-level DeepSEAV and BasenjiV annotations**. We report the weighted k-mer enrichment and enrichment p-value for the top significant enriched k-mers ($1 \leq k \leq 5$) for H3K4me3-DeepSEAV-brain-Max and H3K27ac-BasenjiV-brain-Max which were jointly significant in the brain-specific variant-level joint model. The Bonferonni corrcection threshold is $0.05/(4 * 682)$ where 682 is the number of k-mers analyzed for the 2 significant variant-level annotations analyzed here and the 2 significant allelic-effect annotations analyzed in Table 11.

H3K4me3-DeepSEAV-brain-Max		
kmer	enrichment	pvalue
CGCGC	8.8	9.6e-55
CGGCG	8.7	2.4e-53
CGCCG	8.5	1.2e-50
CGCG	5.9	5.1e-23
CGCGG	5.7	2.1e-21
CCGCG	5.7	5.0e-21
CGCGA	5.0	7.5e-16
AGCGC	4.8	1.0e-14
CCCGC	4.4	3.9e-12
CGCCC	4.3	1.1e-11
CCGCC	4.1	2.1e-10
ACGCG	4.1	2.2e-10
CCGGC	4.0	1.2e-09
CGGC	4.0	1.4e-09
CGGCC	4.0	1.4e-09
CGCGT	3.9	2.4e-09
CCGC	3.8	6.3e-09
CGCC	3.8	7.4e-09
CGGGC	3.8	9.7e-09
CGAGC	3.7	2.6e-08
CGCAG	3.6	6.2e-08
CGC	3.6	9.3e-08
CTGCG	3.6	1.5e-07
CGCTC	3.4	6.2e-07
CTCGC	3.4	1.1e-06

H3K27ac-BasenjiV-brain-Max		
kmer	enrichment	pvalue
CGGCG	2.8	3.3e-72
CGCGC	2.8	6.4e-70
CGCCG	2.7	1.1e-66
CGCG	2.1	6.7e-27
CGCGG	2	7.4e-22
CCGCG	1.9	2.2e-21
CGCGA	1.9	9.8e-20
AGCGC	1.9	1.2e-18
ACGCG	1.7	2.1e-12
CCCGC	1.7	8.9e-12
CGCCC	1.7	2.3e-11
CGCGT	1.7	3.1e-11
CGAGC	1.6	1.1e-10
CGGC	1.6	2.8e-10
CGCAG	1.6	3.8e-10
CTGCG	1.6	4.4e-10
CCGCC	1.6	1.5e-09
CCGGC	1.6	4.3e-09
CGGCC	1.6	4.7e-09
CCGC	1.6	1.5e-08
CGCC	1.6	1.7e-08
CGC	1.5	2.3e-08
CCGCT	1.5	3.5e-08
CGCTC	1.5	3.4e-08
AGCGG	1.5	4.7e-08

Supplementary Figures

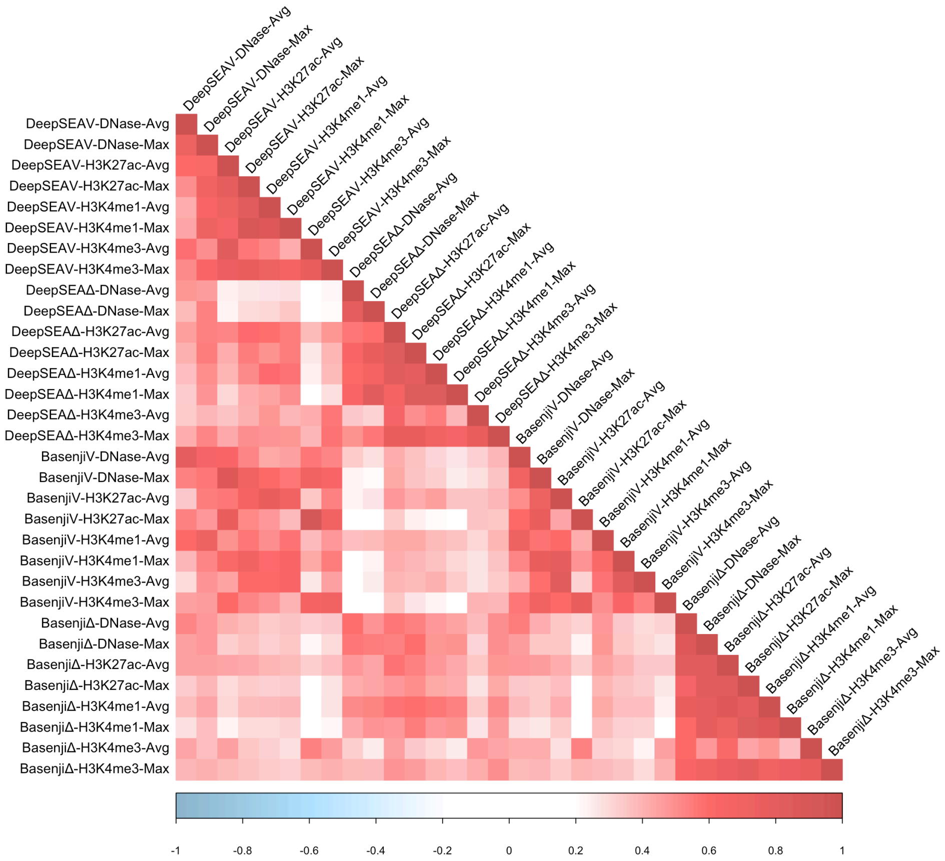

Supplementary Figure 1. **Correlations between deep learning annotations**. We report correlations between each pair of variant-level (DeepSEA and Basenji) and/or allelic-effect (DeepSEAΔ and BasenjiΔ) annotations. When comparing pairs of annotations that differed only in their aggregation strategy (Avg/Max), chromatin mark (DNase/H3K27ac/H3K4me1/H3K4me3), model (DeepSEA/Basenji) or type (variant-level/allelic-effect), respectively, we observed large correlations across aggregation strategies (average $r = 0.71$), chromatin marks (average $r = 0.58$), models (average $r = 0.54$) and types (average $r = 0.48$).

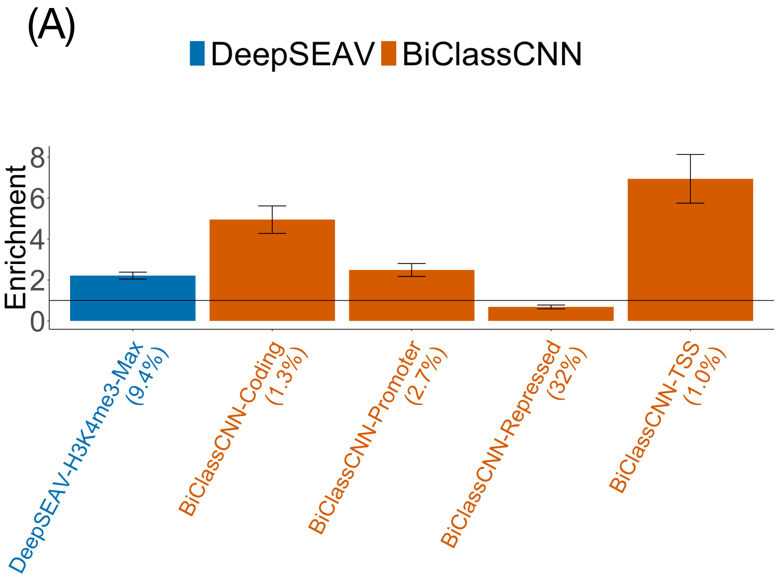

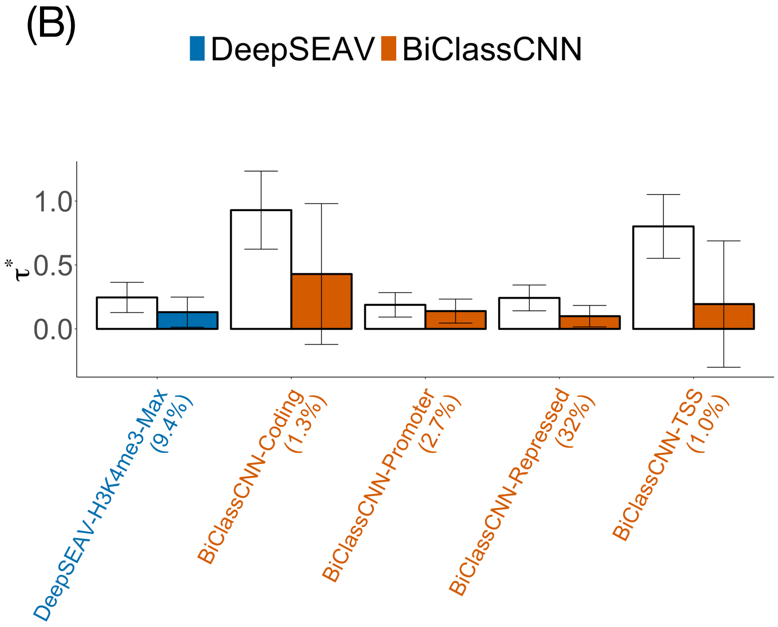

Supplementary Figure 2. Disease informativeness of non-tissue-specific variant-level deep learning annotations: (A) Heritability enrichment, conditional on the baseline-LD model. Horizontal line denotes no enrichment. (B) Standardized effect size ($\tau^{\star}$) conditional on either the baseline-LD model (marginal analyses: left column, white) or the baseline-LD model plus 1 Roadmap, 1 ChromHMM, 3 LocalGCcontent and 2 pLI annotations (non-tissue-specific variant-level joint model: right column, shaded). Results are meta-analyzed across 41 traits. Results are displayed only for annotations with significant $\tau^{\star}$ in marginal analyses after correcting for 106 (variant-level + allelic-effect) non-tissue-specific annotations tested (p-value $P < 0.05/106$ for a two-sided test). None of these annotations were significant after conditioning on the non-tissue-specific variant-level joint model (right column). Error bars denote 95% confidence intervals. Numerical results are reported in Supplementary Table 29, Supplementary Table 30 and Supplementary Table 34.

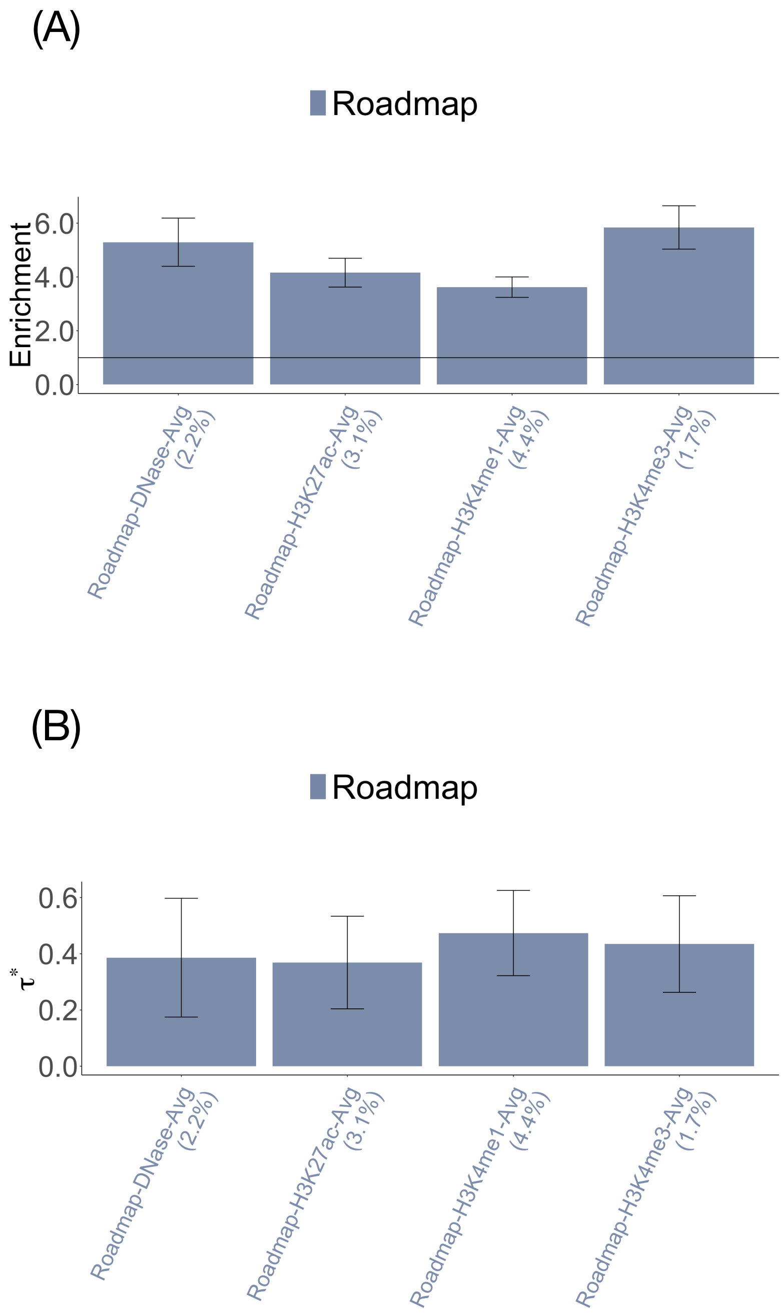

Supplementary Figure 3. Disease informativeness of non-tissue-specific Roadmap annotations in marginal analysis. (A) Heritability enrichment, conditioned on the baseline-LD model. Horizontal line denotes no enrichment. (B) Standardized effect size ($\tau^{\star}$) conditioned on the baseline-LD model. Results are meta-analyzed across 41 traits. Results are displayed only for annotations with significant $\tau^{\star}$ in marginal analyses after correcting for 106 (variant-level + allelic-effect) non-tissue-specific annotations tested (p-value $P < 0.05/106$ for a two-sided test). Error bars denote 95% confidence intervals. Numerical results are reported in Supplementary Table 32.

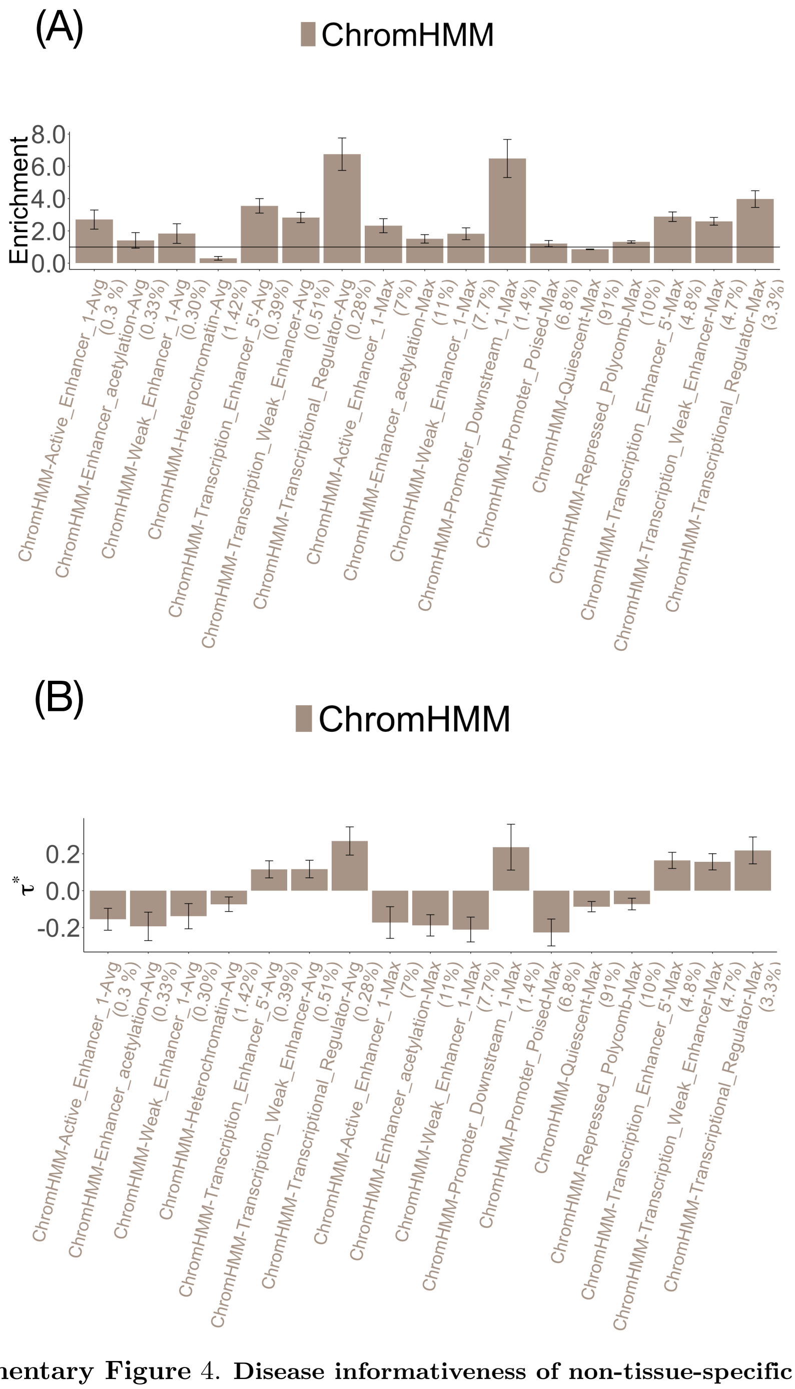

Supplementary Figure 4. **Disease informativeness of non-tissue-specific ChromHMM annotations in marginal analysis**. (A) Heritability enrichment, conditioned on the baseline-LD model. Horizontal line denotes no enrichment. (B) Standardized effect size ($\tau^{\star}$) conditioned on the baseline-LD model. Results are meta-analyzed across 41 traits. Results are displayed only for annotations with significant $\tau^{\star}$ in marginal analyses after correcting for 106 (variant-level + allelic-effect) non-tissue-specific annotations tested (p-value $P < 0.05/106$ for a two-sided test). Error bars denote 95% confidence intervals. Numerical results are reported in Supplementary Table 32.

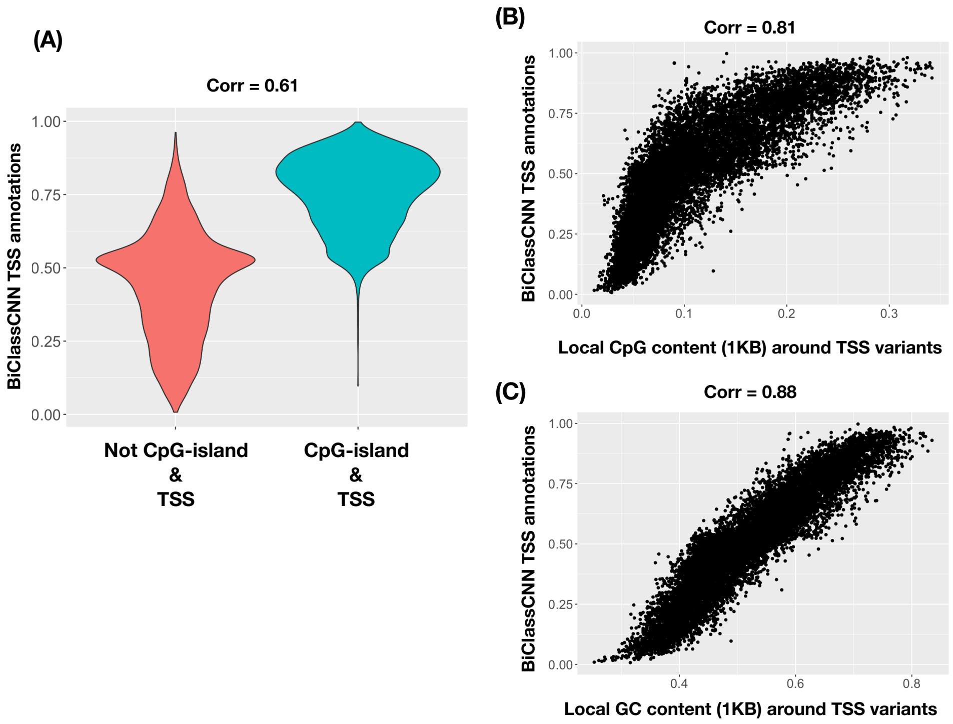

Supplementary Figure 5. **BiClassCNN-TSS is highly correlated with CpG-island, local CpG-content(±1kb) and local GC-content(±1kb) annotations**. (A) Violin plot of the BiClassCNN TSS annotation for variants that are TSS and fall in a CpG island and variants that are TSS but do not fall in a CpG island. (B) Scatter plot of the BiClassCNN TSS annotation with the local GC content (1KB) around the variant for all TSS variants. (C) Scatter plot of the BiClassCNN TSS annotation with the local CpG content (1KB) around the variant for all TSS variants. Here, by TSS variants, we imply variants that are annotated to fall in a TSS region as predicted by Segway[39,40]. The BiClassCNN TSS annotations showed strong correlation with all three features - CpG-island, Local GC and Local CpG content at the TSS variants with the correlation with the Local GC content at TSS variants being the strongest.

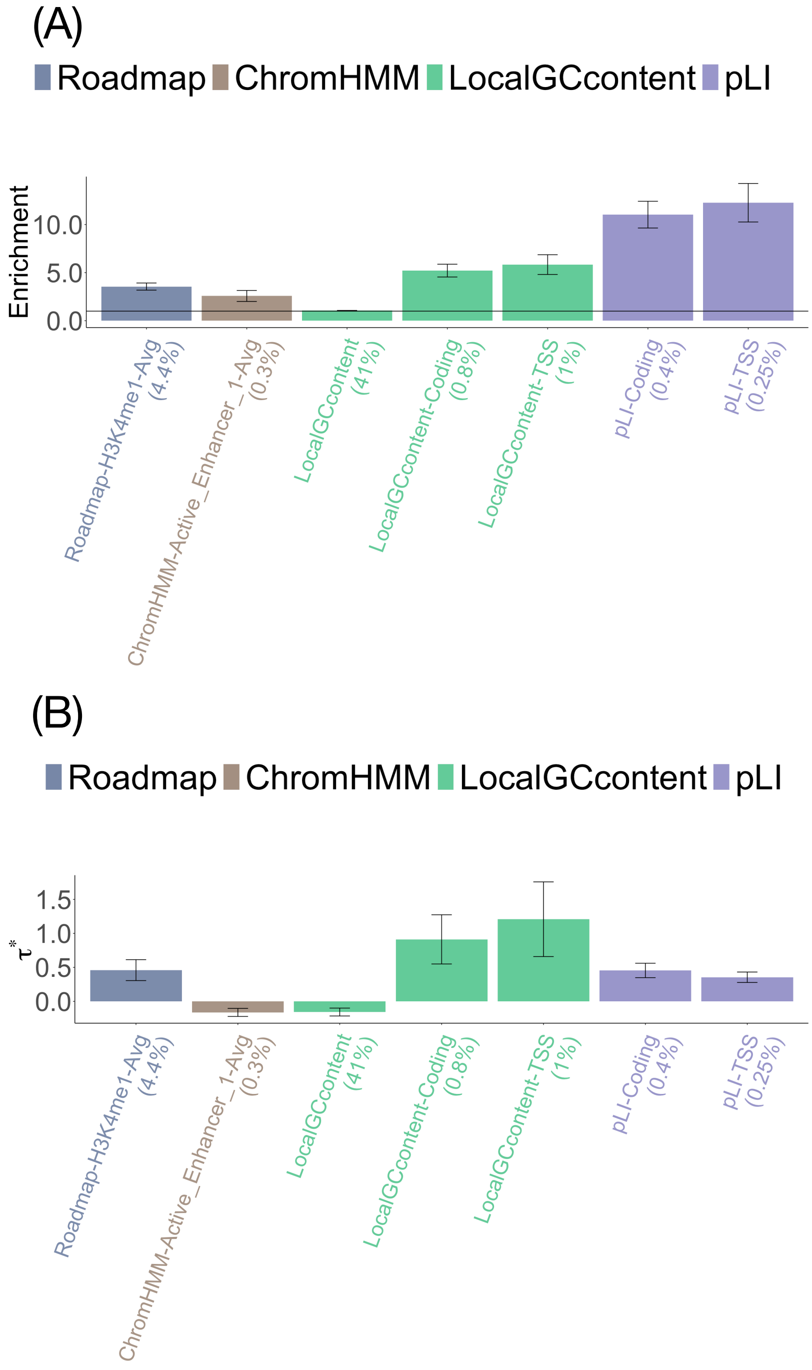

Supplementary Figure 6. Disease informativeness of non-tissue-specific Roadmap, ChromHMM, LocalGCcontent and pLI annotations in non-tissue-specific variant-level joint model. (A) Heritability enrichment, conditioned on the baseline-LD model. Horizontal line denotes no enrichment. (B) Standardized effect size ($\tau^\star$) conditioned on each other as well as the baseline-LD model.Results are meta-analyzed across 41 traits. Results are displayed only for annotations with significant $\tau^\star$ in marginal analyses after correcting for 106 (variant-level + allelic-effect) non-tissue-specific annotations tested (p-value $P < 0.05/106$ for a two-sided test). Error bars denote 95% confidence intervals. Numerical results are reported in Supplementary Table 34.

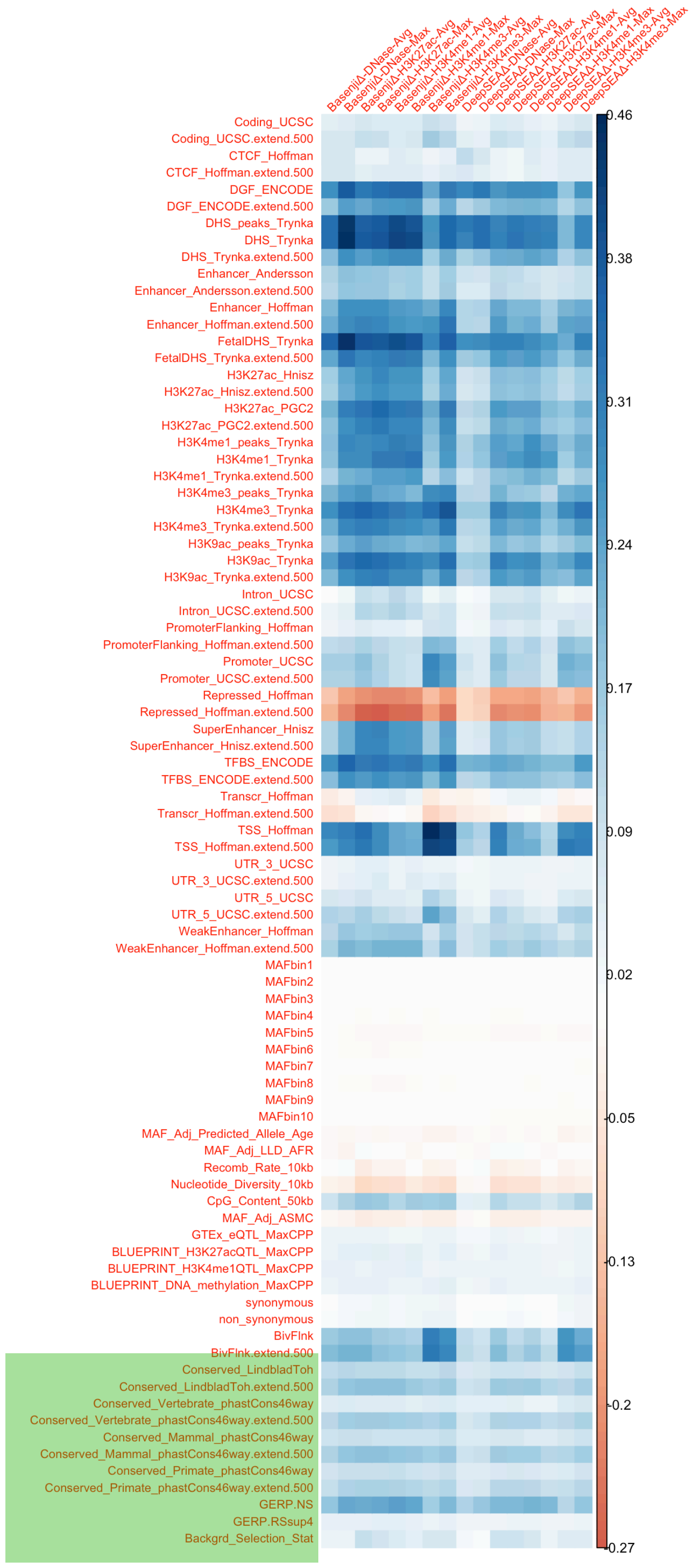

Supplementary Figure 7. Correlations between non-tissue-specific deep learning annotations and the baselineLD v2.1 annotations. We report correlations for 16 non-tissue-specific allelic-effect deep learning annotations and the 85 baseline-LD annotations (excluding the annotation with all 1s). The 11 conservation related annotations are reported at the bottom of the table and shaded green.

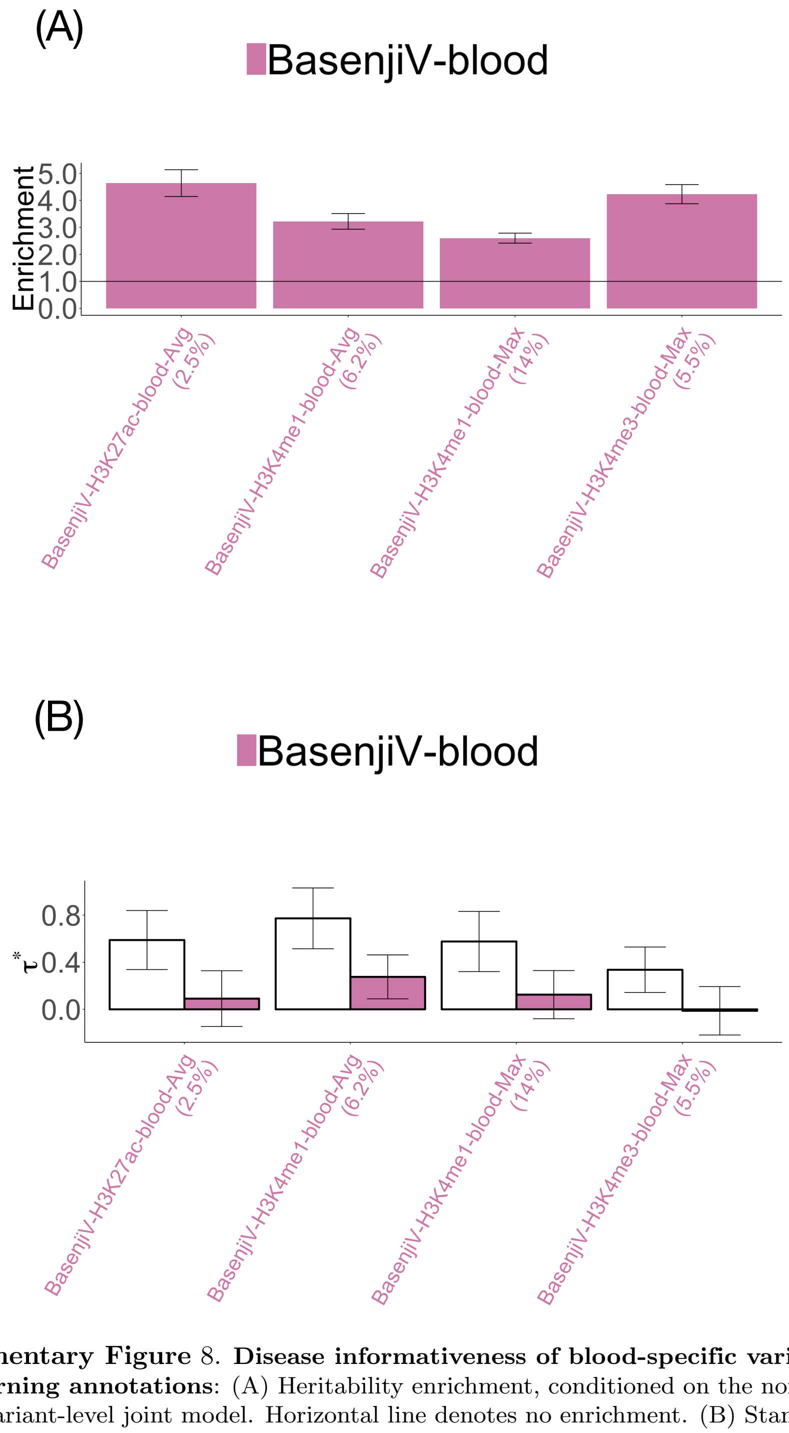

Supplementary Figure 8. **Disease informativeness of blood-specific variant-level deep learning annotations**: (A) Heritability enrichment, conditioned on the non-tissue-specific variant-level joint model. Horizontal line denotes no enrichment. (B) Standardized effect size $\tau^{\star}$ conditioned on either the non-tissue-specific variant-level joint model (marginal analysis: left column, white) or the non-tissue-specific variant-level joint model plus 2 Roadmap and 4 ChromHMM blood-specific annotations (blood-specific variant-level joint model: right column, dark shading). Results are meta-analyzed across 11 blood-related traits. Results are displayed only for annotations with significant $\tau^{\star}$ in marginal analyses after correcting for 80 (variant-level + allelic-effect) blood-specific annotations tested (p-value $P < 0.05/80$ for a two-sided test). None of these annotations were significant after conditioning on the blood-specific variant-level joint model. Error bars denote 95% confidence intervals. Numerical results are reported in Supplementary Table 39 and Supplementary Table 41.

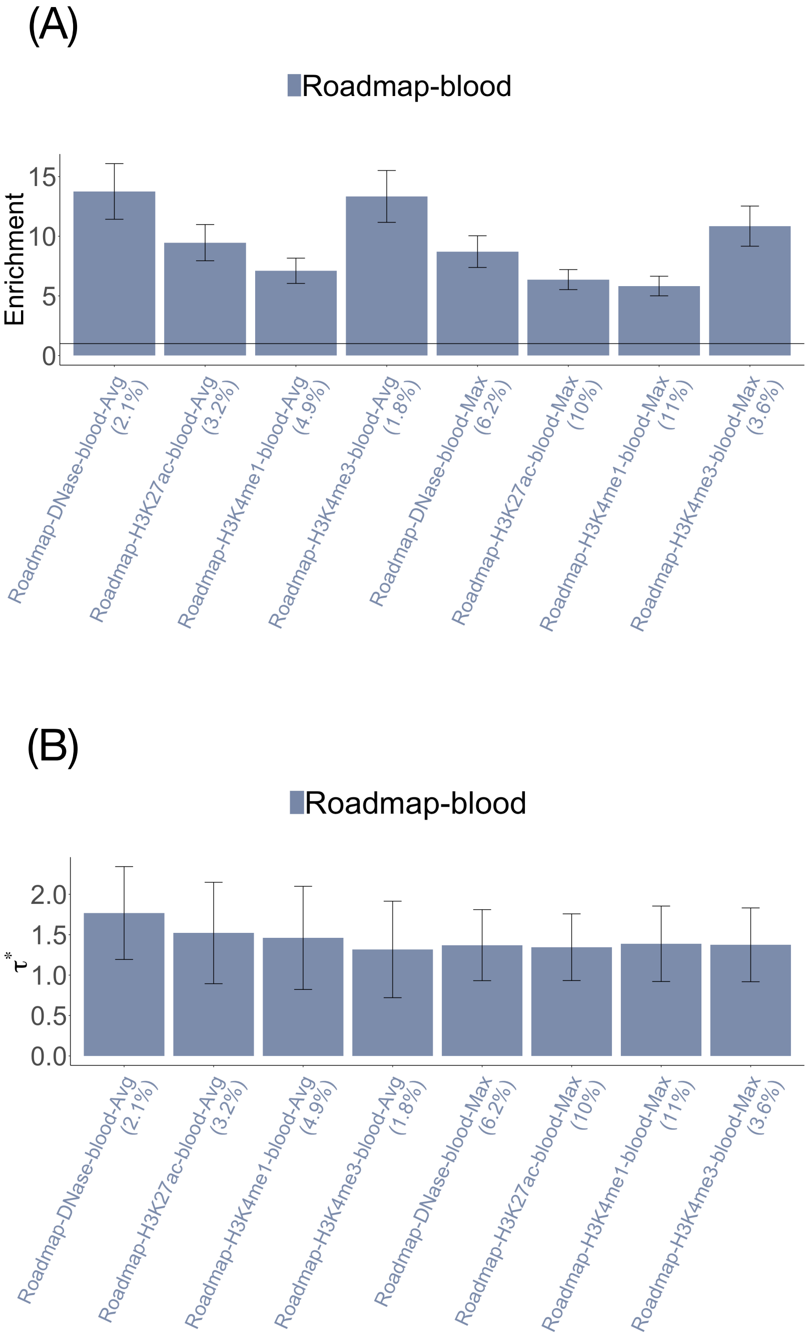

Supplementary Figure 9. Disease informativeness of blood-specific Roadmap annotations in marginal analysis conditional on the non-tissue-specific variant-level joint model. (A) Heritability enrichment, conditioned on the non-tissue-specific variant-level joint model (original baseline-LD annotations and the annotations from Supplementary Figure 6). Horizontal line denotes no enrichment. (B) Standardized effect size ($\tau^\star$) conditioned on the non-tissue-specific variant-level joint model. Results are meta-analyzed across 11 blood-related traits. Results are displayed only for annotations with significant $\tau^\star$ in marginal analyses after correcting for 80 (variant-level + allelic-effect) blood-specific annotations tested (p-value $P < 0.05/80$ for a two-sided test). Error bars denote 95% confidence intervals. Numerical results are reported in Supplementary Table 40.

(A)

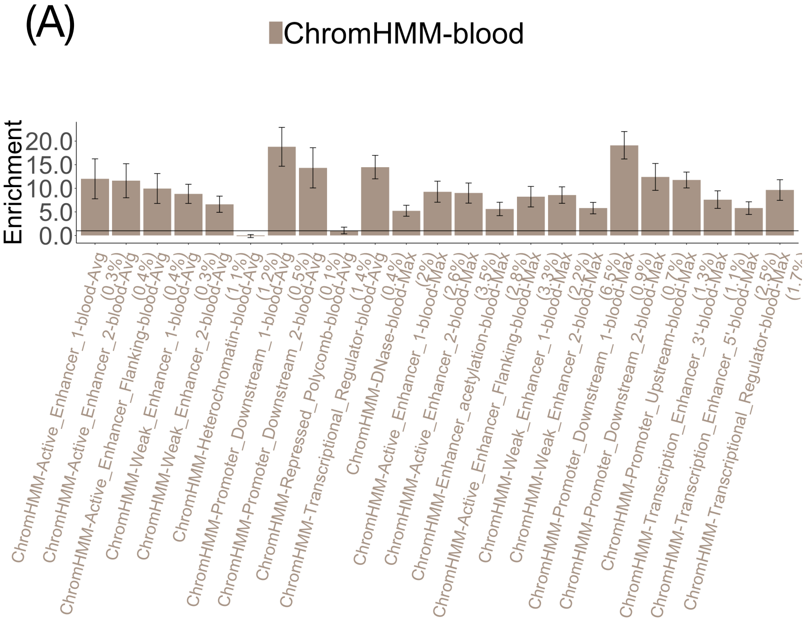

(B)

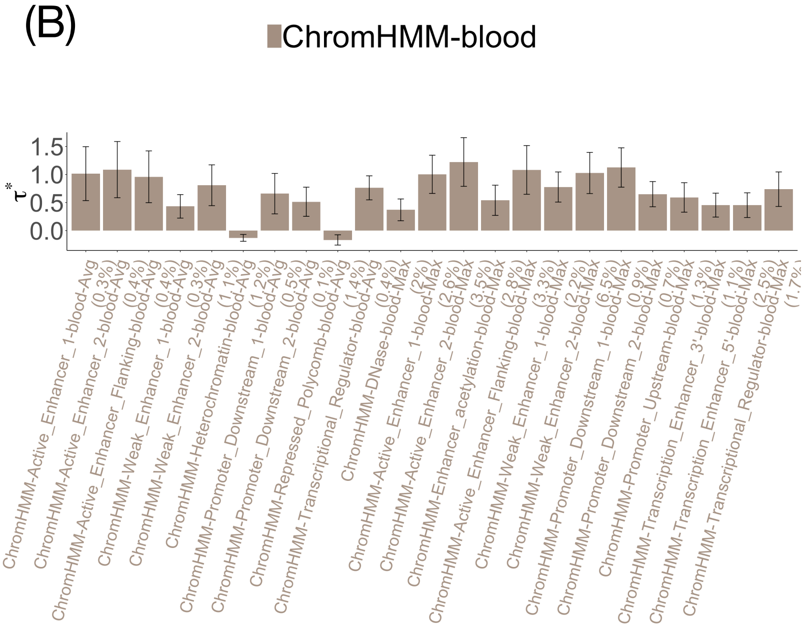

Supplementary Figure 10. **Disease informativeness of blood-specific ChromHMM annotations in marginal analysis conditional on the non-tissue-specific variant-level joint model**. (A) Heritability enrichment, conditioned on the non-tissue-specific variant-level joint model (original baseline-LD annotations and the annotations from Supplementary Figure 6). Horizontal line denotes no enrichment. (B) Standardized effect size ($\tau^\star$)conditioned on the non-tissue-specific variant-level joint model. Results are meta-analyzed across 11 blood-related traits. Results are displayed only for annotations with significant $\tau^\star$ in marginal analyses after correcting for 80 (variant-level + allelic-effect) blood-specific annotations tested (p-value $P < 0.05/80$ for a two-sided test). Error bars denote 95% confidence intervals. Numerical results are reported in Supplementary Table 40.

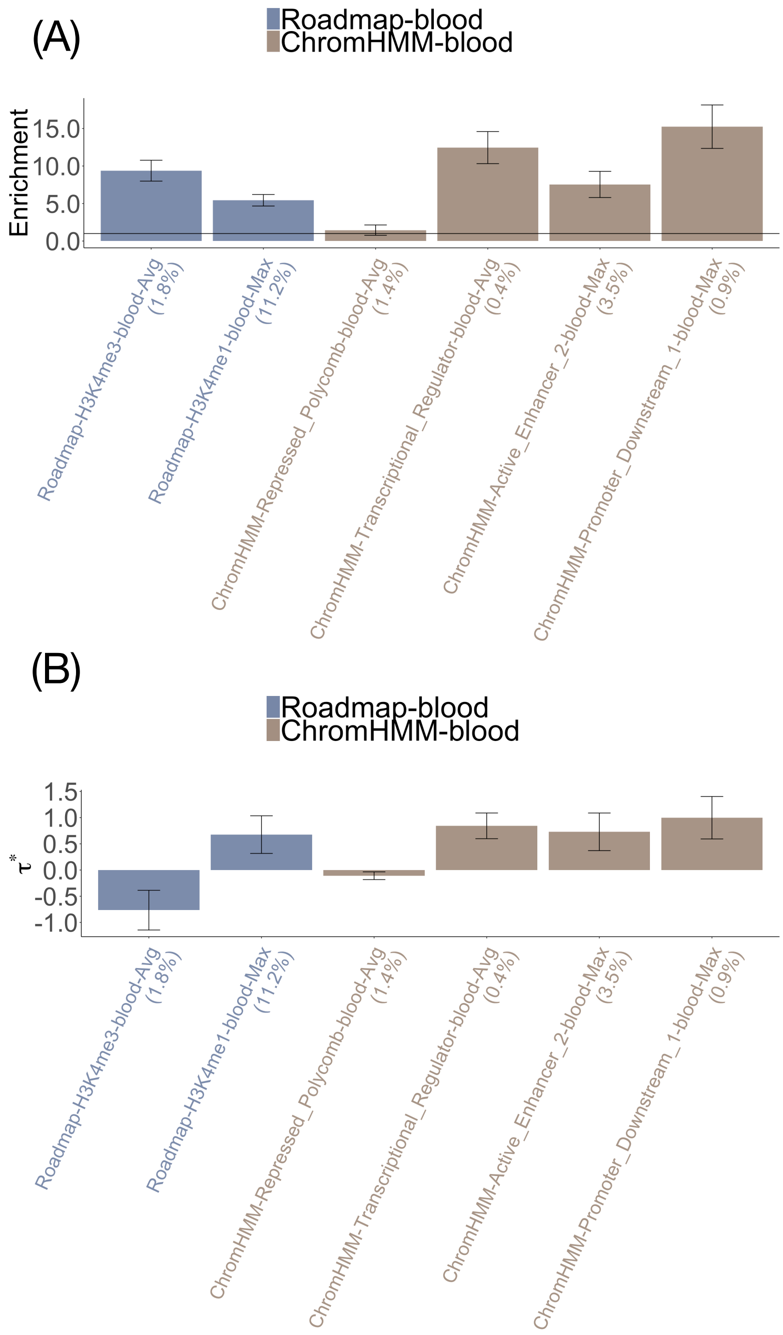

Supplementary Figure 11. **Disease informativeness of blood-specific Roadmap and ChromHMM annotations in blood-specific final joint model**. (A) Heritability enrichment, conditioned on each other and non-tissue-specific variant-level joint model (original baseline-LD annotations and the annotations from Supplementary Figure 6). Horizontal line denotes no enrichment. (B) Standardized effect size ($\tau^{\star}$) conditioned on each other and the non-tissue-specific variant-level joint model. Results are meta-analyzed across 11 blood-related traits. Results are displayed only for annotations with significant $\tau^{\star}$ in marginal analyses after correcting for 80 (variant-level + allelic-effect) blood-specific annotations tested (p-value $P < 0.05/80$ for a two-sided test). Error bars denote 95% confidence intervals. Numerical results are reported in Supplementary Table 41.

(A)

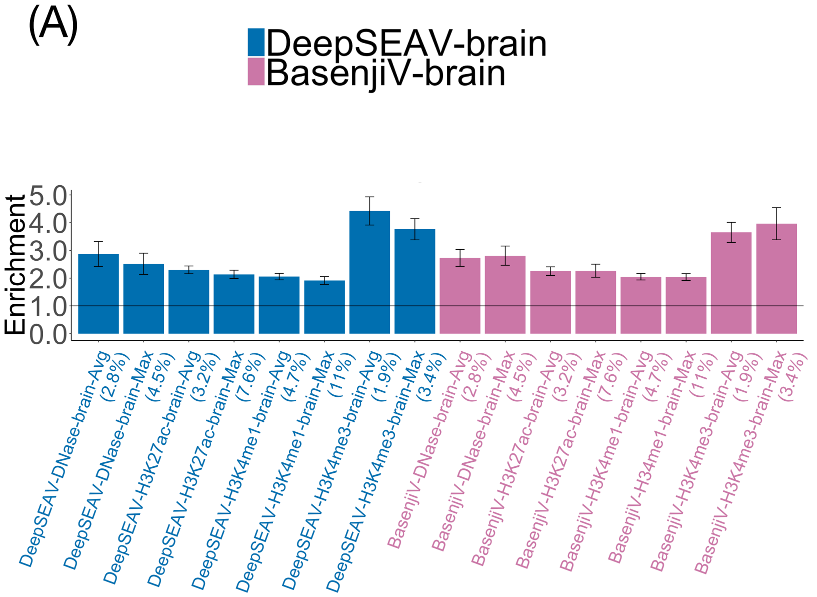

(B)

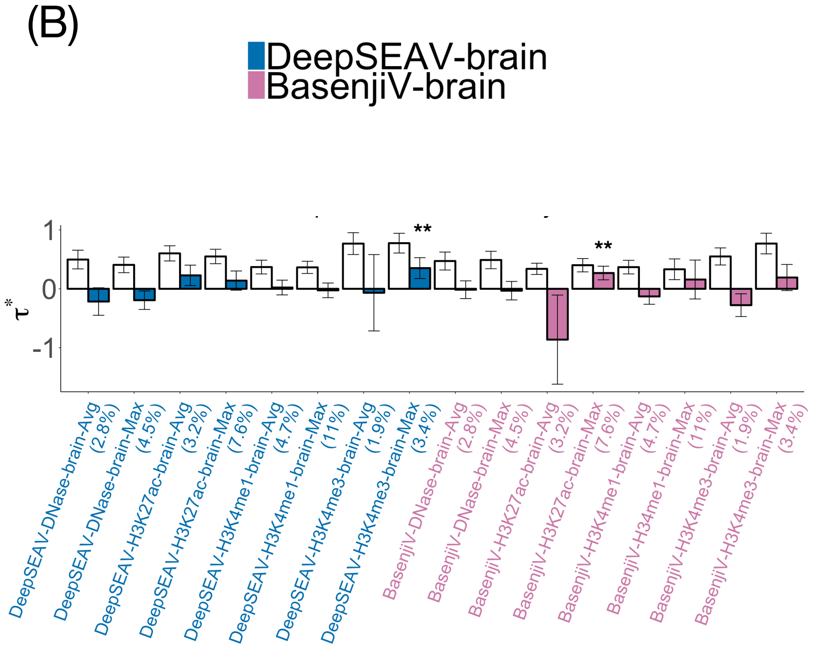

Supplementary Figure 12. **Disease informativeness of brain-specific variant-level deep learning annotations**: (A) Heritability enrichment, conditioned on the non-tissue-specific variant-level joint model. Horizontal line denotes no enrichment. (B) Standardized effect size $\tau^\star$ conditioned on either the non-tissue-specific variant-level joint model (marginal analysis: left column, white) or the non-tissue-specific variant-level joint model plus 1 DeepSEA, 1 Basenji, 1 Roadmap and 3 ChromHMM brain-specific annotations (brain-specific variant-level joint model: right column, dark shading). Results are meta-analyzed across 8 brain-related traits. Results are displayed only for annotations with significant $\tau^\star$ in marginal analyses after correcting for 80 (variant-level + allelic-effect) brain-specific annotations tested ($P < 0.05/80$). For brain-specific variant-level joint model, ** denotes $P < 0.05/80$ for a two-sided test. Error bars denote 95% confidence intervals. Numerical results are reported in Supplementary Table 45 and Supplementary Table 47.

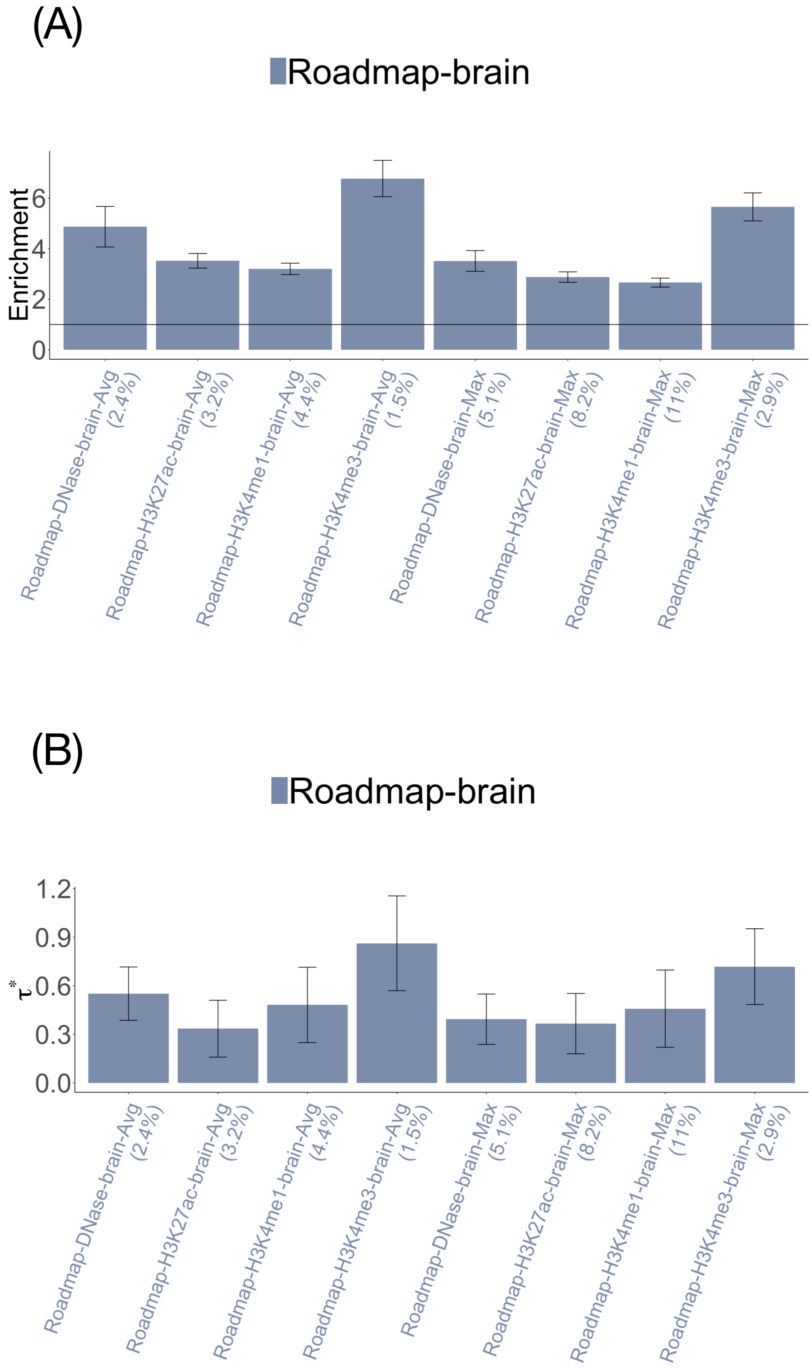

Supplementary Figure 13. **Disease informativeness of brain-specific Roadmap annotations in marginal analysis conditional on the non-tissue-specific variant-level joint model**. (A) Heritability enrichment, conditioned on the non-tissue-specific variant-level joint model (original baseline-LD annotations and the annotations from Supplementary Figure 6). Horizontal line denotes no enrichment. (B) Standardized effect size ($\tau^\star$) conditioned on the non-tissue-specific variant-level joint model. Results are meta-analyzed across 8 brain-related traits. Results are displayed only for annotations with significant $\tau^\star$ in marginal analyses after correcting for 80 (variant-level + allelic-effect) blood-specific annotations tested (p-value $P < 0.05/80$ for a two-sided test). Error bars denote 95% confidence intervals. Numerical results are reported in Supplementary Table 46.

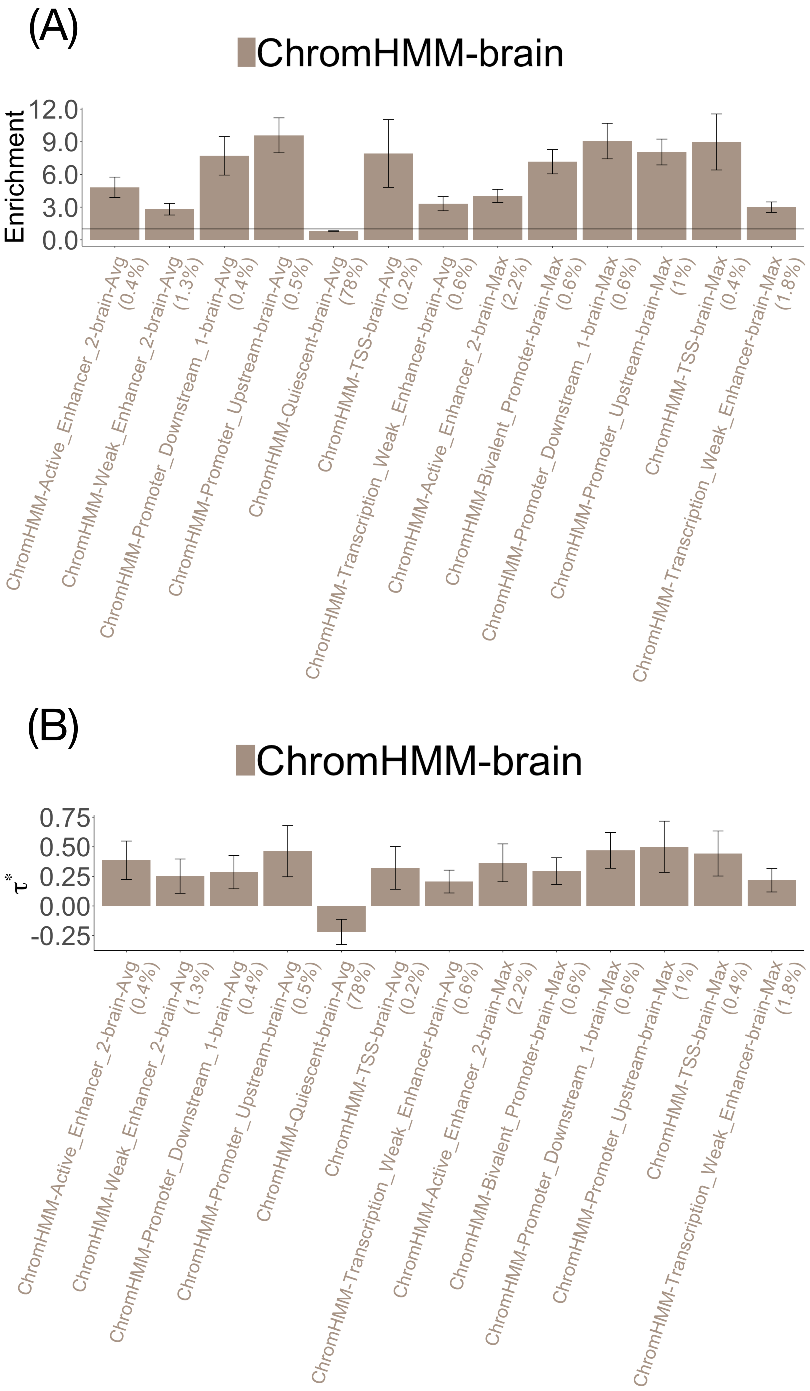

Supplementary Figure 14. **Disease informativeness of brain-specific ChromHMM annotations in marginal analysis conditional on the non-tissue-specific variant-level joint model**. (A) Heritability enrichment, conditioned on the non-tissue-specific variant-level joint model (original baseline-LD annotations and the annotations from Supplementary Figure 6). Horizontal line denotes no enrichment. (B) Standardized effect size ($\tau^\star$) conditioned on the non-tissue-specific variant-level joint model. Results are meta-analyzed across 8 brain-related traits. Results are displayed only for annotations with significant $\tau^\star$ in marginal analyses after correcting for 80 (variant-level + allelic-effect) blood-specific annotations tested (p-value $P < 0.05/80$ for a two-sided test). Error bars denote 95% confidence intervals. Numerical results are reported in Supplementary Table 46.

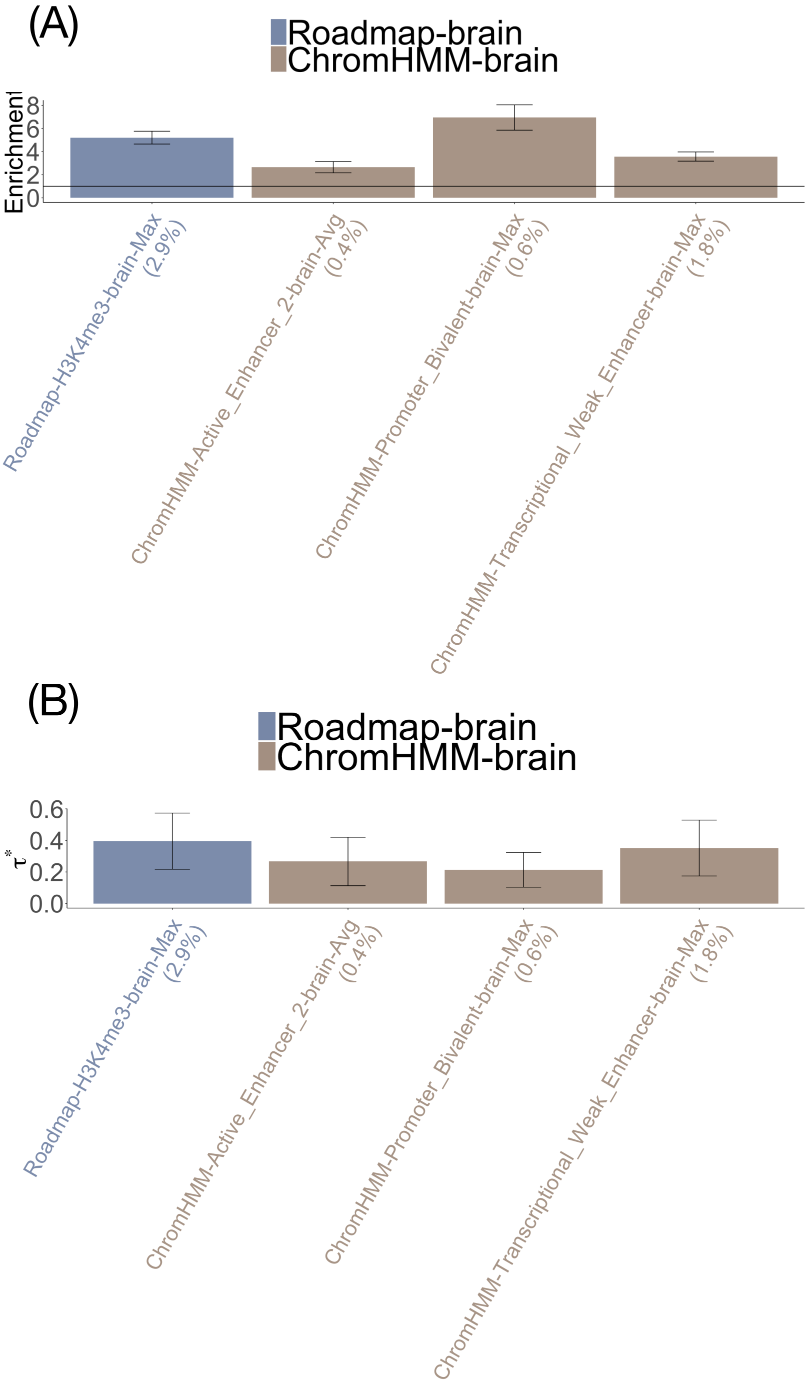

Supplementary Figure 15. **Disease informativeness of brain-specific Roadmap and ChromHMM annotations in brain-specific final joint model**. (A) Heritability enrichment, conditioned on the non-tissue-specific variant-level joint model (original baseline-LD annotations and the annotations from Supplementary Figure 6), and all brain-specific variant-level DeepSEA and Basenji, and Roadmap and ChromHMM annotations. Horizontal line denotes no enrichment. (B) Standardized effect size ($\tau^\star$) conditioned on the non-tissue-specific variant-level joint model and all brain-specific variant-level DeepSEA and Basenji, and Roadmap and ChromHMM annotations. Results are meta-analyzed across 8 brain-related traits. Results are displayed only for annotations with significant $\tau^\star$ in marginal analyses after correcting for 80 (variant-level + allelic-effect) blood-specific annotations tested (p-value $P < 0.05/80$ for a two-sided test). Error bars denote 95% confidence intervals. Numerical results are reported in Supplementary Table 47.

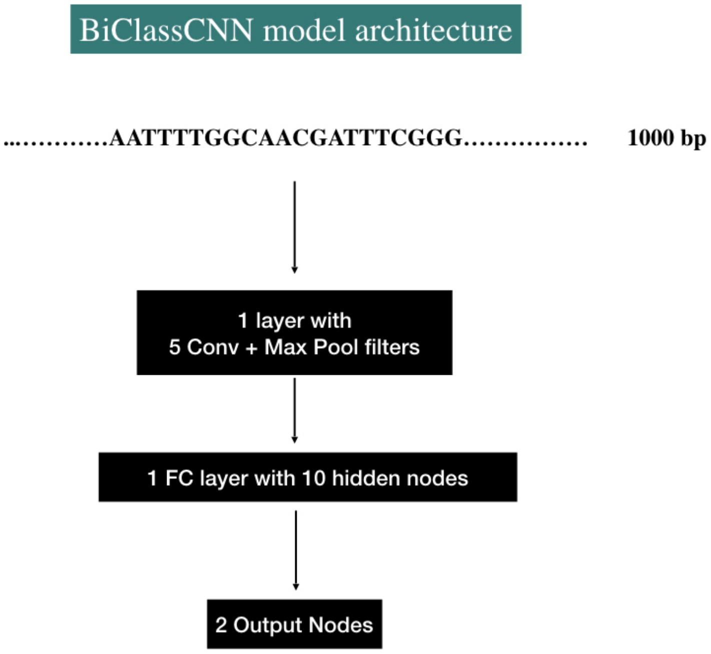

Supplementary Figure 16. **The Neural net architecture for BiClassCNN**. The architecture of the Covolutional Neural Net (CNN) model used for BiClassCNN training. It comprioses of two layers - 1 layer being Convolutional+MaxPool with 5 filters and 1 Fully Connected hidden layer with 10 nodes leading to the final output layer with 2 nodes distinguishing between the positive and negative sets. We chose the convolutional filter to be of size 4 and stride length 1 and the MaxPool filter to pool every 4 bases at a time. RELU activation was used.

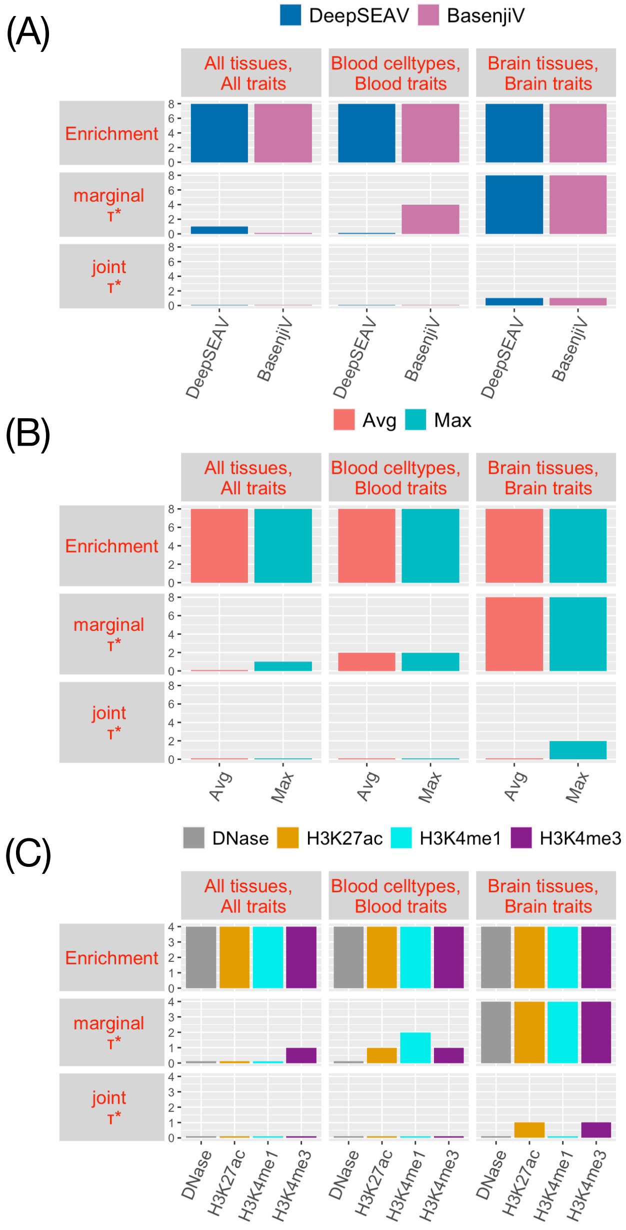

Supplementary Figure 17. **Number of significantly disease informative variant-level deep learning annotations**: Number of significant non-tissue-specific, blood-specific and brain-specific variant-level annotations across (A) different deep learning models (DeepSEA and Basenji), (B) different aggregation strategies (Average and Maximum) and (C) different types of epigenomic marks, in terms of marginal enrichment in heritability, conditional $\tau^{\star}$ and joint $\tau^{\star}$ for all traits, blood traits and brain traits respectively. For all traits analysis, the analysis is conditioned on the baseline-LD model. For blood traits analysis, the analysis is conditioned on non-tissue-specific variant-level joint model. For brain traits analysis, the analysis is conditioned on non-tissue-specific variant-level joint model. Numerical results are reported in Supplementary Table 28 (numerical summary of results), Supplementary Table 29 (enrichment and marginal $\tau^{\star}$ for all tissues, all traits analysis), Supplementary Table 34 (joint $\tau^{\star}$ of all tissues, all traits analysis), Supplementary Table 39 (enrichment and marginal $\tau^{\star}$ for blood cell types, blood traits analysis), Supplementary Table 41 (joint $\tau^{\star}$ of blood cell types, blood traits analysis), Supplementary Table 45 (enrichment and marginal $\tau^{\star}$ for brain tissues, brain traits analysis) and Supplementary Table 47 (joint $\tau^{\star}$ of brain tissues, brain traits analysis).

Supplementary References

1. Kundaje, A. *et al.* Integrative analysis of 111 reference human epigenomes. *Nature* **518**, 317–330 (2015).

2. Ernst, J. & Kellis, M. Large-scale imputation of epigenomic datasets for systematic annotation of diverse human tissues. *Nature biotechnology* **33**, 364–376 (2015).

3. Ernst, J. & Kellis, M. ChromHMM: automating chromatin-state discovery and characterization. *Nature methods* **9**, 215–216 (2012).

4. Ernst, J. & Kellis, M. Chromatin-state discovery and genome annotation with ChromHMM. *Nature protocols* **12**, 2478–2492 (2017).

5. Gazal, S. *et al.* Linkage disequilibrium–dependent architecture of human complex traits shows action of negative selection. *Nature genetics* **49 (10)**, 1421–1427 (2017).

6. Zhou, J. *et al.* Deep learning sequence-based ab initio prediction of variant effects on expression and disease risk. *Nature genetics* **50**, 1171–1179 (2018).

7. Kelley, D. *et al.* Sequential regulatory activity prediction across chromosomes with convolutional neural networks. *Genome research* **28**, 739–750 (2018).

8. Lek, M. *et al.* Analysis of protein-coding genetic variation in 60,706 humans. *Nature* **536**, 285–291 (2016).

9. Adzhubei, I. *et al.* A method and server for predicting damaging missense mutations. *Nature methods* **7**, 248–249 (2010).

10. Adzhubei, I., Jordan, D. & Sunyaev, S. Predicting functional effect of human missense mutations using PolyPhen-2. *Current protocols in human genetics* **76**, 7–20 (2013).

11. Kumar, P., Henikoff, S. & Ng, P. Predicting the effects of coding non-synonymous variants on protein function using the SIFT algorithm. *Nature protocols* **4**, 1073–1081 (2009).

12. MacArthur, J. *et al.* The new NHGRI-EBI Catalog of published genome-wide association studies (GWAS Catalog). *Nucleic acids research* **45(D1)**, D896–D901 (2017).

13. Zhou, J. & Troyanskaya, O. Predicting effects of noncoding variants with deep learning–based sequence model. *Nature methods* **12**, 931–934 (2015).

14. Hormozdiari, F. *et al.* Leveraging molecular quantitative trait loci to understand the genetic architecture of diseases and complex traits. *Nature genetics* **50(7)**, 1041–1047 (2018).

15. Hormozdiari, F. *et al.* Functional disease architectures reveal unique biological role of transposable elements. *Nature Communications* **10(1)**, 4054 (2019).

16. Finucane, H. *et al.* Partitioning heritability by functional annotation using genome-wide association summary statistics. *Nature genetics* **47**, 1228–1235 (2015).

17. Farh, K. *et al.* Genetic and epigenetic fine mapping of causal autoimmune disease variants. *Nature* **518**, 337–343 (2015).

18. Bycroft, C. *et al.* The uk biobank resource with deep phenotyping and genomic data. *Nature* **562(7726)**, 203–209 (2018).

19. Jostins, L. *et al.* Host-microbe interactions have shaped the genetic architecture of inflammatory bowel disease. *Nature* **491**, 119–124 (2012).

20. Okada, Y. *et al.* Genetics of rheumatoid arthritis contributes to biology and drug discovery. *Nature* **506**, 376–381 (2014).

21. Bentham, J. *et al.* Genetic association analyses implicate aberrant regulation of innate and adaptive immunity genes in the pathogenesis of systemic lupus erythematosus. *Nature genetics* **47(12)**, 1457–1464 (2015).

22. Dubois, P. *et al.* Multiple common variants for celiac disease influencing immune gene expression. *Nature genetics* **42(4)**, 295–302 (2010).

23. Speliotes, E. *et al.* Association analyses of 249,796 individuals reveal 18 new loci associated with body mass index. *Nature genetics* **42**, 937–948 (2010).

24. Okbay, A. *et al.* Genome-wide association study identifies 74 loci associated with educational attainment. *Nature* **533**, 539–542 (2016).

25. Ripke, S. *et al.* Biological insights from 108 schizophrenia-associated genetic loci. *Nature* **511(7510)**, 421–427 (2014).

26. Tobacco & Genetics Consortium, . Genome-wide meta-analyses identify multiple loci associated with smoking behavior. *Nature genetics* **42**, 441–447 (2010).

27. Group., P. G. C. B. D. W. Large-scale genome-wide association analysis of bipolar disorder identifies a new susceptibility locus near odz4. *Nature genetics* **43**, 977–983 (2011).

28. Barban, N. *et al.* Genome-wide analysis identifies 12 loci influencing human reproductive behavior. *Nature genetics* **48(12)**, 1462–1472 (2016).

29. Boraska, V. *et al.* A genome-wide association study of anorexia nervosa. *Molecular psychiatry* **19(10)**, 1085–1094 (2014).

30. of the Psychiatric Genomics Consortium., C.-D. G. Identification of risk loci with shared effects on five major psychiatric disorders: a genome-wide analysis. *The Lancet* **381(9875)**, 1371–1379 (2013).

31. Schunkert, H. *et al.* Large-scale association analysis identifies 13 new susceptibility loci for coronary artery disease. *Nature genetics* **43**, 333–338 (2011).

32. Teslovich, T. *et al.* Biological, clinical and population relevance of 95 loci for blood lipids. *Nature* **466**, 707–713 (2010).

33. Lango, A. *et al.* Hundreds of variants clustered in genomic loci and biological pathways affect human height. *Nature* **467**, 832–838 (2010).

34. Morris, A. *et al.* Large-scale association analysis provides insights into the genetic architecture and pathophysiology of type 2 diabetes. *Nature genetics* **44**, 981–990 (2012).

35. Lindblad-Toh, K. *et al.* A high-resolution map of human evolutionary constraint using 29 mammals. *Nature* **478**, 476–482 (2011).

36. Siepel, A. *et al.* Evolutionarily conserved elements in vertebrate, insect, worm, and yeast genomes. *Genome research* **15**, 1034–1050 (2005).

37. Davydov, E. *et al.* Identifying a high fraction of the human genome to be under selective constraint using GERP++. *PLoS computational biology* **6**, e1001025 (2010).

38. McVicker, G. *et al.* Widespread genomic signatures of natural selection in hominid evolution. *PLoS genetics* **5**, e1000471 (2009).

39. Hoffman, M. *et al.* A method to predict the impact of regulatory variants from DNA sequence. *Nucleic acids research* **41**, 827–841 (2012).

40. Hoffman, M. *et al.* Unsupervised pattern discovery in human chromatin structure through genomic segmentation. *Nature methods* **9**, 473–476 (2012).
