# Supplementary material for: Evaluating the informativeness of deep learning annotations for human complex diseases
Source: Nat Commun. 2020 Sep 17;11:4703. doi: 10.1038/s41467-020-18515-4 (PMC7499261; doi:10.1038/s41467-020-18515-4)
Supplement: Supplementary file 3 — Reporting Summary [file 41467_2020_18515_MOESM3_ESM.pdf]

nature research

Corresponding author(s): Kushal K Dey

Last updated by author(s): Aug 12, 2020

Reporting Summary

Nature Research wishes to improve the reproducibility of the work that we publish. This form provides structure for consistency and transparency in reporting. For further information on Nature Research policies, see our Editorial Policies and the Editorial Policy Checklist.

Statistics

For all statistical analyses, confirm that the following items are present in the figure legend, table legend, main text, or Methods section.

n/a	Confirmed	
☐	☒	The exact sample size (*n*) for each experimental group/condition, given as a discrete number and unit of measurement
☐	☒	A statement on whether measurements were taken from distinct samples or whether the same sample was measured repeatedly
☐	☒	The statistical test(s) used AND whether they are one- or two-sided<br>*Only common tests should be described solely by name; describe more complex techniques in the Methods section.*
☐	☒	A description of all covariates tested
☐	☒	A description of any assumptions or corrections, such as tests of normality and adjustment for multiple comparisons
☐	☒	A full description of the statistical parameters including central tendency (e.g. means) or other basic estimates (e.g. regression coefficient) AND variation (e.g. standard deviation) or associated estimates of uncertainty (e.g. confidence intervals)
☐	☒	For null hypothesis testing, the test statistic (e.g. *F*, *t*, *r*) with confidence intervals, effect sizes, degrees of freedom and *P* value noted<br>*Give P values as exact values whenever suitable.*
☒	☐	For Bayesian analysis, information on the choice of priors and Markov chain Monte Carlo settings
☒	☐	For hierarchical and complex designs, identification of the appropriate level for tests and full reporting of outcomes
☐	☒	Estimates of effect sizes (e.g. Cohen's *d*, Pearson's *r*), indicating how they were calculated

Our web collection on statistics for biologists contains articles on many of the points above.

Software and code

Policy information about availability of computer code

Data collection	We did not collect data for this study. We analyzed raw genotype-phenotype data from UK Biobank (application 16549). Summary statistics analyzed are available at https://data.broadinstitute.org/alkesgroup/UKBB.
Data analysis	Our ldsc software is available at https://github.com/bulik/ldsc.<br>Our baseline-LD model version 2.1 is available at https://data.broadinstitute.org/alkesgroup/LDSCORE.<br>All deep learning annotations and additional scripts for processing and training BiClassCNN are available at https://data.broadinstitute.org/alkesgroup/LDSCORE/DeepLearning.

For manuscripts utilizing custom algorithms or software that are central to the research but not yet described in published literature, software must be made available to editors and reviewers. We strongly encourage code deposition in a community repository (e.g. GitHub). See the Nature Research guidelines for submitting code & software for further information.

Data

Policy information about availability of data

All manuscripts must include a data availability statement. This statement should provide the following information, where applicable:
- Accession codes, unique identifiers, or web links for publicly available datasets
- A list of figures that have associated raw data
- A description of any restrictions on data availability

The URL section of the paper contains the links of all the publicly available summary statistics analyzed, and all new deep learning based annotations and other competing annotations that we generated. Additionally we add a Data Availability section.

Field-specific reporting

Please select the one below that is the best fit for your research. If you are not sure, read the appropriate sections before making your selection.

- [x] Life sciences
- [ ] Behavioural & social sciences
- [ ] Ecological, evolutionary & environmental sciences

For a reference copy of the document with all sections, see nature.com/documents/nr-reporting-summary-flat.pdf

Life sciences study design

All studies must disclose on these points even when the disclosure is negative.

Sample size	We used the larger sample size that was available. We restricted our analysis to relatively independent traits.
Data exclusions	We excluded the HLA from all analyses and analyzed only autosomes.
Replication	No replication dataset was analyzed as our analyses are already based on a meta-analyses of several independent traits.
Randomization	We performed no randomization and analyzed all individuals for the GWAS studies investigated.
Blinding	We did not collect data for this study and used publicly available GWAS data and UK Biobank data.

Reporting for specific materials, systems and methods

We require information from authors about some types of materials, experimental systems and methods used in many studies. Here, indicate whether each material, system or method listed is relevant to your study. If you are not sure if a list item applies to your research, read the appropriate section before selecting a response.

Materials & experimental systems

n/a	Involved in the study	
x		Antibodies
x		Eukaryotic cell lines
x		Palaeontology and archaeology
x		Animals and other organisms
x		Human research participants
x		Clinical data
x		Dual use research of concern

Methods

n/a	Involved in the study	
x		ChIP-seq
x		Flow cytometry
x		MRI-based neuroimaging
