# Supplementary material for: Evaluating the informativeness of deep learning annotations for human complex diseases
Source: Nat Commun. 2020 Sep 17;11:4703. doi: 10.1038/s41467-020-18515-4 (PMC7499261; doi:10.1038/s41467-020-18515-4)
Supplement: Supplementary file 2 — Peer Review File [file 41467_2020_18515_MOESM2_ESM.pdf]

Editorial Note: This manuscript has been previously reviewed at another journal that is not operating a transparent peer review scheme. This document only contains reviewer comments and rebuttal letters for versions considered at Nature Communications.

REVIEWERS' COMMENTS:

Reviewer #1 (Remarks to the Author):

I've read over the other review, authors response and the updated manuscript. It's now much easier to follow and the conclusions themselves are valuable (as they were in the original submission, just hidden!). I'm happy to recommend acceptance at Nat Comms.

REVIEWERS' COMMENTS:

Reviewer #1 (Remarks to the Author):

I've read over the other review, authors response and the updated
manuscript. It's now much easier to follow and the conclusions
themselves are valuable (as they were in the original submission, just hidden!). I'm happy to
recommend acceptance at Nat Comms.

We thank the reviewer for reviewing our paper and for recommending
acceptance.
